# Picrotoxane Sesquiterpene Glycosides and a Coumarin Derivative from *Coriaria* *nepalensis* and Their Neurotrophic Activity

**DOI:** 10.3390/molecules21101344

**Published:** 2016-10-12

**Authors:** Yuan-Yuan Wang, Jun-Mian Tian, Cheng-Chen Zhang, Bo Luo, Jin-Ming Gao

**Affiliations:** Shaanxi Key Laboratory of Natural Products & Chemical Biology, College of Science, Northwest A&F University, Yangling 712100, Shaanxi, China; 15829461284@163.com (Y.-Y.W.); tianjunmian@sohu.com (J.-M.T.); 08cxzk3zc@163.com (C.-C.Z.); luobo2011@163.com (B.L.)

**Keywords:** *Coriaria nepalensis*, *Coriariaceae*, natural products, picrotoxane, sesquiterpene, neurite outgrowth-potentiating activity, neurotrophic activity

## Abstract

Two picrotoxane sesquiterpene lactone glycosides, nepalactones A (**1**) and B (**2**), and one new coumarin, nepalarin (**3**), were isolated from the root barks of the poisonous plant *Coriaria* *nepalensis*. Their structures were elucidated via HRESIMS and 1D and 2D NMR spectroscopic analyses, and further verified via transformation methods. In addition, compounds **1**–**3** and five semisynthetic congeners (**1a**–**e**) were assayed for the activity to induce neurite outgrowth in rat pheochromocytoma (PC12) cells. As a result, nepalactone A derivative **1c** and nepalarin (**3**) significantly enhanced nerve growth factor (NGF)-mediated neurite outgrowth in PC12 cells.

## 1. Introduction

**Figure 1:**
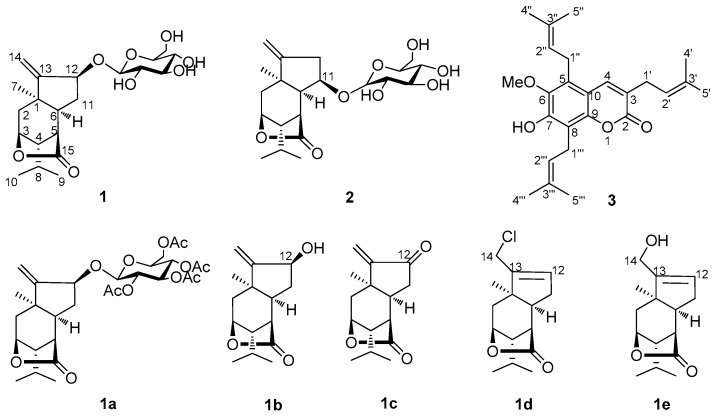
Chemical structures of compounds **1**–**3** and **1a**–**e**.

*Coriaria nepalensis* Wall (*Coriaria sinica* Maxim) (Coriariaceae) is a Chinese medicine herb mainly distributed in the southwest of China. Traditionally, this herb is used to treat numbness, toothache, traumatic injury, and acute conjunctivitis [1]. Sesquiterpene lactones are the characteristic bioactive constituents from family Coriariaceae [2,3,4,5,6,7,8,9]. A series of sesquiterpenes [6,7,8,9] and prenyled coumarins [10,11] have been previously identified from *C. nepalensis*. Among these sesquiterpene lactones, coriamytin and tutin have been suggested as main bioactive substances for the treatment of schizophrenia [8,12], and as insecticidal agents [9]. In our recent effort to search for neurotrophic natural products [13,14,15,16], we employed rat dopaminergic PC12 cells as an in vitro cell model to screen various natural compounds for the effect on nerve growth factor (NGF)-induced neurite outgrowth. The active compounds are expected to be potentially useful for the treatment of Alzheimer’s disease and other neurological disorders [17,18]. Here, two new picrotoxane-type sesquiterpene glycosides, nepalactones A (**1**) and B (**2**), and one new polyprenyled coumarin, nepalarin (**3**) (Figure 1), were isolated from the root barks of this plant. We describe herein the isolation and structure elucidation of the three new compounds as well as the assay for the ability to stimulate NGF-mediated neurite outgrowth from PC12 cells.

## 2. Results and Discussion

**Figure 2:**
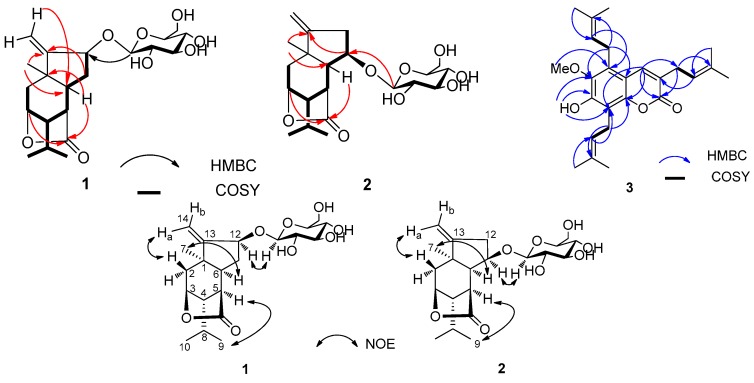
Selected 2D NMR correlations for compounds **1**–**3**.

Compound **1** was isolated as a white, amorphous powder. The molecular formula was established as C_21_H_32_O_8_ via HRESIMS (*m/z* [M + Na]^+^ 435.1991; calcd. 435.1995) in combination with the ^13^C-NMR spectrum, requiring six degrees of unsaturation. Its IR spectrum indicated the presence of hydroxy (3563 cm^−1^) and γ-lactone carbonyl (1765 cm^−1^) groups. The ^13^C and DEPT NMR spectra of **1** (Table 1) exhibited 21 carbon signals corresponding to three methyl groups, four methylene groups (including one olefinic carbon at δ 111.5 and one oxygenated carbon at δ 61.6), 11 methine groups (seven oxygenated carbons including five carbons of sugar), and three quaternary carbons (one lactone carbonyl at δ 180.6 and one olefinic carbon at δ 158.2). The ^1^H and ^13^C-NMR spectra of **1** displayed signals for tertiary methyl (δ 1.25, s, 3H), isopropyl (δ 0.99, 1.01, d, each 3H, and δ 1.83, m, 1H), and exocyclic olefinic bond (δ_H_ 5.21, s, 1H, 5.42, s, 1H) groups. A series of sugar signals were also detected in the ^1^H-NMR spectrum (Table 1) between δ_H_ 3.22–3.87, with an anomeric proton at δ_H_ 4.35 (d, 1H, *J*= 7.5 Hz, H-1′). The ^13^C-NMR spectrum indicated the presence of a glucose unit due to a set of carbon signals at δ_C_ 101.5, 73.5, 76.2, 70.2, 76.6, and 61.6. These data suggested the presence of a glucopyranosyl moiety in **1**. The glucose in nepalactone A derivative **1a** appeared to be tetraacetylated, based on NMR spectra (Table 1) and HRESIMS (*m*/*z* 603.2416 [M + Na]^+^). Furthermore, the ^1^H and ^13^C-NMR data of **1** were similar to those of the known picrotoxane sesquiterpenoid, corialactone D (**1b**), which has been previously identified from the same plant [8], except for the presence of the glucose unit at C-12 in **1**. The attachment of the glucose moiety to C-12 of the aglycone **1b** was deduced from the HMBC correlations from H-1′ (δ_H_ 4.35) to C-12 (δ_C_ 79.4). These spectroscopic data suggested that **1** is a glucoside of corialactone D. The ^1^H-^1^H COSY and HSQC correlations indicated the presence of two substructures–CH_2_(2)–CH(3)–CH(4)–CH(5)–CH(6)–CH(11)–CH(12)–, and –CH(4)–CH(8)[CH_3_(9)]–CH_3_(10)–units, whose connectivity was defined by the key HMBC correlations (Figure 2).

The relative configuration of the aglycone (**1b**) of **1** was defined by a NOESY experiment (Figure 2). The NOE correlations of H_3_-7/H-6, H_3_-9/H-5, and H-12/H-1′ indicated that CH_3_-7, H-5, H-6, H-12, and the isopropyl group were at the same orientation (α-configuration). Additionally, enzymatic hydrolysis of **1** afforded the known intact aglycone **1b**, which was identified from ^1^H NMR, and its absolute configuration (1*R*,3*S*,4*S*,5*R*,6*S*,12*S*) was previously determined from CD spectrum [8]. In addition, the coupling constant (*J* = 7.5 Hz) of the anomeric proton (δ_H_ 4.35, H-1′) suggested a *β*-configuration for the glucose unit. Indeed, compound **1** was hydrolyzable via *β*-glucocidase. GC analysis suggested that acid hydrolyzate of **1** contained the glucose moiety with the d-configuration. Hence, the structure of **1** was determined unambiguously as shown, and named nepalactone A.

**Scheme 1:**
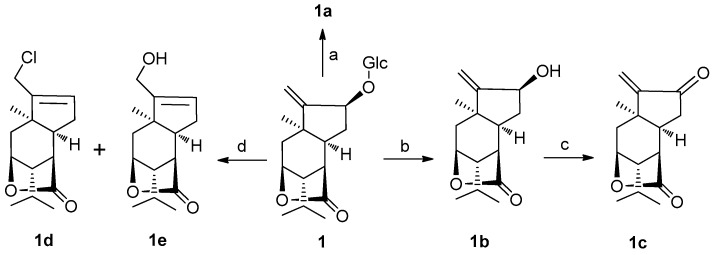
Conversion of **1** into picrotoxane sesquiterpenes **1a**–**e**. Reagents and conditions: (**a**) acetic anhydride, pyridine, r.t.; (**b**) citrate buffer, *β*-glucocidase, rt; (**c**) Dess–Martin periodinane (DMP), DCM, r.t., 40 min; (**d**) 4 N HCl-dioxane (1:1), 100 °C, 2 h.

In addition, in order to enhance molecular diversity of sesquiterpene compounds, additional three derivatives of **1**. The conversion of the aglycone **1b** using Dess-Martin periodinane (DMP) in CH_2_Cl_2_ at room temperature afforded **1c**; and treatment of **1** with 4 N HCl in dioxane at 100 °C yielded two compounds **1d** and **1e** (Scheme 1). These three new congeners were characterized by an application of spectroscopic data and HRESIMS.

Compound **2** was obtained as white powder with a molecular formula C_21_H_32_O_8_, as established via HRESIMS (*m*/*z* [M + Na]^+^ 435.1987; calcd. 435.2417). Its molecular formula was identical to that of **1**, indicating that **2** was a positional isomer of **1**. The similar IR spectrum of **2** compared to **1** indicated the presence of similar functional groups. The ^1^H and ^13^C-NMR spectral data (Table 1) of **2** were highly similar to those of nepalactone A (**1**). Compound **1** significantly differed from **2** by the signals for the glucopyranosyl group, attached at C-12 for **1** while at C-11 (δ_C_ 81.9) for **2**. The HMBC correlations from the anomeric proton H-1′ at δ_H_ 4.33 (d, *J* = 7.5 Hz) to C-11 (δ_C_ 81.9) implied that the glucose group is attached to C-11 (Figure 2). The stereochemistry of **2** was established from a NOESY experiment (Figure 2). The most significant NOE correlations observed were between H-6/H_3_-7, H-5/H_3_-9, and H-1′/H-11, indicating that H-5, H-6, H-6, the methyl group at C-1, and the isopropyl group at C-4 were all on the same α-face of the molecule. The NMR data of **2** were fully assigned by analysis of the 2D-NMR data including its HSQC, HMBC, and ^1^H-^1^H COSY spectra. Thus, the structure of **2** was established as shown for nepalactone B.

Compound **3**, yellowish crystalline needles, was assigned the molecular formula C_2__5_H_3__2_O_4_ from [M − H]^−^ at *m/z* 395.2226 in HRESIMS, requiring 10 degrees of unsaturation. The IR spectrum displayed the presence of hydroxy (3319 cm^−1^), C=O (1691 cm^−1^) groups and a benzene ring (1627, 1583, and 1458 cm^−1^). The ^13^C-NMR and DEPT data showed 25 carbon signals for seven methyls, three methylenes, four methines, and 11 quaternary carbons. The ^13^C-NMR data suggested the presence of three isopentenyl groups (δ_C_ 119.8, δ_H_ 5.34, δ_C_ 135.2; and δ_C_ 121.1, δ_H_ 5.33, δ_C_ 133.1; and δ_C_ 122.6, δ_H_ 5.11, δ_C_ 132.8), one methoxy group (δ_C_ 62.2). Besides three isopentenyl and one methoxy substituent, the NMR characteristic signals and UV spectra were indicative of a coumarin skeleton [11]. One downfield singlet (δ_H_ 7.51, s) in the ^1^H-NMR spectrum of **3** suggested that **3** was a pentasubstituted coumarin. Furthermore, the ^1^H and ^13^C-NMR data of **3** were similar to those of the known coumarin, 7-hydroxy-6-methoxy-3,8-bis(3-methyl-2-butenyl)coumarin, which was previously identified from the same plant [11], except for the presence of an additional isopentenyl unit [δ_H_ 5.11 (1H, m, H-2′′), 3.56 (2H, d, *J* = 6.7 Hz, H-1′′), 1.86 (s, H-5′′), 1.75 (s, H-4′′); δ_C_ 132.8 (C-3′′), 122.6 (C-2′′), 25.6 (C-4′′), 24.7 (C-1′′), 18.1 (C-5′′)] at C-5 in 3. The location of the isopentenyl unit at C-5 was supported by the HMBC correlations of H-2′′ to C-5, and H-1′′ to C-6 and C-10. Full analyses of the ^1^H and ^13^C-NMR spectral data of **3**, supported by the ^1^H-^1^H COSY, DEPT, HSQC, and HMBC experiments (Figure 2), permitted the assignment of all proton and carbon resonances. Consequently, the structure of **3** was established as 7-hydroxy-6-methoxy-3,5,8-tri(3-methyl-2-butenyl) coumarin and named nepalin C. To our knowledge, this is a rare coumarin with three isopentenyl units in natural source.

### NeuriteOutgrowth-Promoting Activity

Compounds (**1**–**3**, and **1a**–**1e**) were evaluated for the effect on NGF-induced neurite outgrowth in rat pheochromocytoma PC12 cells according to our previously reported procedures [17,18].

**Figure 3:**
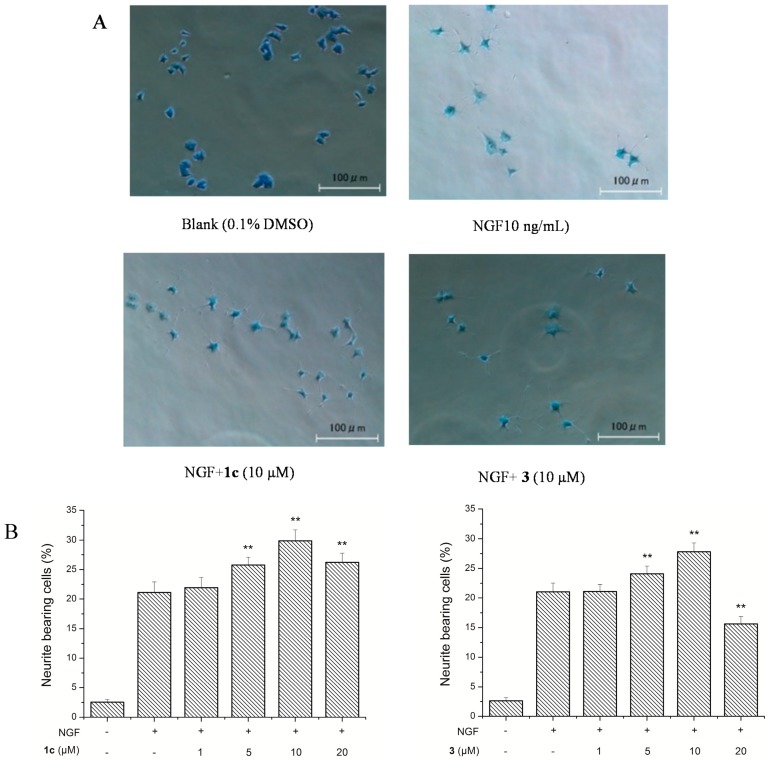
Neurite outgrowth-promoting activity of **1c** and **3** in PC12 cells. (**A**) Morphology of PC12 cells; (**B**) Quantitative analysis of neurite outgrowth promoted by **1c** and **4**. Compounds **1c** and **3** (1, 5, 10, and 20 μM) + NGF (10 ng/mL). Data are expressed as the means ± SD (*n* = 3) (** *p* < 0.01 vs. NGF; Dunnett’s *t* test). In control experiments, the percentages of neurite-bearing cells were 21.11% ± 1.62% following incubation with 10 ng/mL of NGF after 48 h.

All these compounds at the concentrations of 1, 5, 10, and 20 μM failed to induce neurite outgrowth from PC12 cells in the absence of NGF. However, in the presence of 2 ng/mL NGF, compounds **2**, **3**, and **1c**–**1e** at a concentration of 10 μM markedly increased the NGF-induced neurite outgrowth based on the numbers of neurite-bearing cells by 21.67% ± 2.64% to 24.40% ± 2.04%, greater than those (17.60% ± 0.24%) of control NGF (2 ng/mL). In particular, compounds **1c** and **3** were selected for the assay of neurotrophic activity. As shown in Figure 3, compounds **1c** and **3** at concentrations ranging from 1 to 10 μM increased the numbers of neurite-bearing cells in a concentration dependent manner. Compared with 21.11% ± 1.62% for 10 ng/mL NGF alone, compounds **1c** and **3** at a concentration of 10 μM increased the numbers of neurite-bearing cells by 29.89% ± 1.86% and 27.81% ± 1.48%, respectively. Nevertheless, compounds **1c** and **3** at 20 μM showed somewhat toxicity to PC12 cells. These results suggested that the attachment of the sugar unit to the picrotoxane skeleton might reduce the neurotrophic activity.

## 3. Experimental Section

### 3.1. General Procedures

Optical rotations were recorded on an Autopol III automatic polarimeter. UV spectra were obtained using a Thermo Scientific Evolution 300 UV-VIS Spectrophotometer (Thermo Fisher Scientific Inc., Waltham, MA, USA). ^1^H and ^13^C-NMR spectra were recorded on 400 MHz and 500 MHz NMR instruments. Chemical shifts were reported using the solvent residual peak as the internal standard. ESI-MS spectra were performed on a Thermo Fisher LTQ Fleet instrument spectrometer (Thermo Fisher Scientific Inc.). HRESIMS data were obtained on an API QSTAR time-offlight spectrometer. Column chromatography (CC) was performed on silica gel (90–150 μm) (Qingdao Marine Chemical Inc., Qingdao, China), Sephadex LH-20 (40–70 μm), and Lichroprep RP-18 gel (40–63 μm; Merck, Darmstadt, Germany). GF254 plates were used for thin-layer chromatography (TLC). Preparative TLC (PTLC) was carried out on silica gel 60 GF254; semi-preparative HPLC was performed via a Thermo BDS Hypersil column (250 mm× 4.6 mm and 250 mm× 10 mm, 5 μm).

### 3.2. Plant Materials

The dried root barks of *Coriaria nepalensis* were collected from the Qinling mountains, in the Shaanxi province of China in November 2013. The plant was authenticated by Professor Wu. A voucher specimen (0023466) was deposited at the Herbarium of College of Life Sciences, Northwest A&F University, P. R. China.

### 3.3. Extraction, Isolation, and Purification

The dried and powdered root-bark of *C. nepalensis* (4.2 kg) were extracted by 95% EtOH at room temperature for three times. After removal of the solvent, the residue (1.9 kg) obtained was suspended in water and then extracted successively with petroleum ether (PE), EtOAc, and *n*-BuOH. The EtOAc portion (911 g) was chromatographed on silica gel over CC eluting with PE/EtOAc mixtures of increasing polarity (9:1, 8:2, 7:3, 6:4, 4:6) to yield five fractions (Frs. 1–5). Fr. 2 was separated by repeated CC over silica gel (EtOAc/MeOH 9:1 to 6:4) to yield five fractions (2-1–2-5). Fraction 2-2 (2 g) was submitted to a RP-18 column (MeOH/H_2_O, 10:90 to 100:0) to yield two further fractions (2-2-1 and 2-2-2), and Fraction 2-2-1 was separated by Sephadex LH-20 (CHCl_3_/MeOH 1:1) and silica gel CC (CHCl_3_/MeOH 8:2) to yield **1** (216 mg). Fraction 2-3 was separated by silica gel CC (PE/acetone 20:1) to afford four fractions (2-3-1–2-3-4). Fraction 2-3-3 was separated by CC on LH-20 (CHCl_3_/MeOH 1:1), RP-18 CC (70% MeOH), and then silica gel with (PE/acetone, 50:1 to 40:1) to afford **3** (1.23 g). The *n*-butanol fraction (242 g) was separated by silica gel CC using EtOAc-MeOH (30:1–15:1) as eluent to afford seven fractions (Frs. A–G). Fr. C was separated by CC over silica gel (CHCl_3_/MeOH, 12:1 to 5:1), Sephadex LH-20 (MeOH), and then RP-18 to yield **1** (500 mg). Purification of Fr. C-3 by LH-20 CC (MeOH) followed by HPLC (40% EtOH, flow rate = 2 mL/min) provided **2** (2 mg, *t*_R_ = 17.8 min).

*Nepalactone A* (**1**). White, amorphous powder; ${\left[\alpha \right]}_{D}^{20}$−26.3 (*c* 0.24, MeOH); IR (KBr)ν_max_ 3402, 1765, 1076, 1025 cm^−1^; ^1^H and ^13^C-NMR data, see Table 1, Figures S1–S9; ESIMS *m/z* 435.17 [M + Na]^+^, 846.62 [2M + Na]^+^; HRESIMS *m/z* 435.1991 [M + Na]^+^ (calcd. for C_21_H_32_O_8_Na, 435.1995).

*Nepalactone B* (**2**). White powder; ${\left[\alpha \right]}_{D}^{20}$ + 6.9 (*c* 0.24, MeOH); IR (KBr) ν_max_ 3390, 1769, 1076, 1028 cm^−1^; ^1^H and ^13^C-NMR data, see Table 1, Figures S19–27; ESIMS *m/z* 823.15 [2M − H]^−^; HRESIMS *m/z* 435.1987 [M + Na]^+^ (calcd. for C_21_H_32_O_8_Na, 435. 1995).

*Nepalarin* (**3**). Pale yellow needles; UV(MeOH): λ_max_ (logε) nm: 221 (4.28), 336 (4.22); IR (KBr) ν_max_ 3355, 1731, cm^−1^; IR (KBr) ν_max_ 3319, 1692, 1583, 1458, 1394, 1306, 1067, 1041, 1015 cm^−1^; ^1^H-NMR (500 MHz, CDCl_3_) δ 7.51 (1H, s, H-4), 5.34 (1H, m, H-2′), 5.33 (1H, m, H-2′′′), 5.11 (1H, m, H-2′′), 3.84 (3H, s, 6-OCH_3_), 3.58 (2H , d, *J* = 7.5 Hz, H-1′′′), 3.56 (2H, d, *J* = 6.7 Hz, H-1′′), 3.26 (2H, d, *J* = 7.0 Hz, H-1′), 1.89 (3H, s, H-4′′′), 1.86 (3H, s, H-5′′), 1.83 (3H, s, H-4′), 1.75(3H, s, H-4′′), 1.72 (3H, s, H-5′′′), 1.71 (3H, s, H-5′); ^13^C-NMR (125 MHz, CDCl_3_) δ 162.3 (C-2), 149.5 (C-7), 149.3 (C-9), 142.0 (C-6), 135.9 (C-4), 135.2 (C-3′), 133.1 (C-3′′′), 132.8 (C-3′′), 128.2 (C-10), 124.6 (C-3), 122.6 (C-2′′), 121.1 (C-2′′′), 119.8 (C-2′), 114.3 (C-8), 111.8 (C-5), 62.2 (6-OCH_3_), 28.5 (C-1′), 25.9 (C-4′), 25.8 (C-5′′′), 25.6 (C-4′′), 24.7 (C-1′′), 22.4 (C-1′′′), 18.1 (C-5′′), 18.0 (C-4′′′), 17.8 (C-5′); ESI-MS *m/z* 419.49 [M + Na]^+^, 815.18 [2M + Na]^+^, 341.19 [M − C_4_H_7_]^+^; ESI-MS *m/z* 395.41 [M − H]^−^; HRESIMS *m/z* 395.2226 [M − H]^−^ (calcd. for C_25_H_31_O_4_, 395.2222).

### 3.4. Nepalactones A Tetraacetate ***1a***

To a solution of **1** (3.2 mg, 0.03 mmol) in pyridine (1 mL), acetic anhydride (1 mL) was added, and the mixture stood at RT for 24 h. The reaction mixture was concentrated to dryness, and the residue were purified on silica gel CC using EtOAc, affording tetraacetate **1a** (ca. 3 mg) as an off-white gum. ${\left[\alpha \right]}_{D}^{20}$−20.9 (*c* 0.2, MeOH); ^1^H and ^13^C-NMR data, see Table 1, Figures S10–18; HRESIMS *m*/*z* [M + Na]^+^ 603.2416 (calcd. for C_29_H_40_O_12_Na, 603.2417).

### 3.5. Acid Hydrolysis of ***1*** and theDetermination of Sugar

Compound **1** (2 mg) was hydrolyzed with 2N HCl-dioxane (1:1, 1 mL) at 100 °C for 2 h. The reaction mixture was partitioned between chloroform and H_2_O three times. The aqueous layer was neutralized with 2 M Ag_2_CO_3_ and evaporated in vacuo. The residue was dissolved in pyridine (0.5 mL), to which l-cysteine methyl ester hydrochloride in pyridine (0.1 M, 0.5 mL) was added. After reacting at 60 °C for 1 h, trimethylsilylimidazole (0.05 mL) was added to the reaction mixture and kept at 4 °C for another 8 h. The mixture was analyzed via GC–MS. By comparison of the retention time of the authentic sample, the monosaccharide of **1** was determined to be d-glucose (*t*_R_ 11.85 min) (*t*_R_ 12.15 min of l-glucose).

### 3.6 Enzymatic Hydrolysis of ***1***

To a solution of **1** (20 mg) in a mixture of a citrate buffer (pH = 5, 1 mL) and water (1 mL), *β*-gluosidase (1.3 mg, Sigma-Aldrich, St. Louis, MO, USA) was added. After the reaction mixture was incubated at 30 °C for 5 days, it was extracted with EtOAc, dried over anhydrous sodium sulfate, concentrated, and purified via column chromatography using MeOH/CHCl_3_ (1:15) to yield aglycon **1b** as a white solid (8.2 mg, 68%), which was identified as a sesquiterpenoid, corialactone D, via comparison with the ^1^H-NMR spectrum. ^1^H-NMR (500 MHz, CDCl_3_) δ 1.73 (d, *J* = 15.0 Hz, H-2α), 2.46 (dd, *J* = 3.5, 15.0 Hz, H-2β), 4.68 (m, H-3), 2.02 (m, H-4), 2.36 (m, H-5), 2.36 (m, H-6), 1.30 (s, 3H, H-7), 1.83 (m, 1H, H-8), 1.02 (d, *J* = 6.9 Hz, 3H, H-9), 1.00 (d, *J* = 6.9 Hz, 3H, H-10), 2.37 (m, 1H, H-11α), 2.10 (m, 1H, H-11β), 4.86 (m, 1H, H-12), 5.33 (s, 1H, H-14a), 5.18 (s, 1H, H-14b).

### 3.7 Preparation of ***1c***

To a solution of **1b** (8.2 mg, 0.03 mmol) in CH_2_Cl_2_ (1 mL) at room temperature, NaHCO_3_ (13.8 mg, 0.16 mmol, 5 equiv.) and Dess-Martin periodinane (DMP) (20.9 mg, 0.05 mmol, 1.5 equiv.) were added. After 40 min of stirring, the reaction mixture was poured into a mixture of a saturated aqueous solution of Na_2_S_2_O_3_ (5 mL), a saturated aqueous solution of NaHCO_3_ (5 mL), and water (5 mL). The layers were separated, and the aqueous phase was extracted with CH_2_Cl_2_. The combined organic extracts were washed with brine, dried over MgSO_4_, filtered, and concentrated under reduced pressure. The crude product was purified via flash chromatography on silica gel (PE/EtOAc 5/1 to 2/1), affording **1c** (7.8 mg, yield 98%) as a slightly yellow oil; ^1^H-NMR (500 MHz, CDCl_3_) δ 6.06 (s, 1H, H-14a), 5.25 (s, 1H, H-14b), 4.66 (t, *J* = 4.0 Hz, 1H, H-3), 2.65 (dd, *J* = 19.3, 8.8 Hz, 1H, H-11α), 2.46 (d, *J* = 19.3 Hz, 1H, H-11β), 2.38 (m, 1H, H-2β), 2.36 (m, 1H, H-5), 2.34 (m, 1H, H-6), 1.99 (m, 1H, H-4), 1.78 (m, 1H, H-8), 1.77 (m, 1H, H-2α), 1.15 (s, 3H, H-7), 0.96 (d, *J* = 7.0 Hz, 3H, H-10), 0.94 (d, *J* = 7.0 Hz, 3H, H-9); ^13^C-NMR (125 MHz, CDCl_3_) δ 203.7 (C-12), 176.7 (C-15), 151.9 (C-13), 117.5 (C-14), 79.0 (C-3), 51.2 (C-4), 45.0 (C-5), 40.1 (C-1), 39.8 (C-11), 35.5 (C-6), 33.5 (C-2), 32.2 (C-7), 25.3 (C-8), 20.6 (C-10), 19.73 (C-9); ESIMS *m/z* 249.08 [M + H]^+^, 271.15 [M + Na]^+^; HRESIMS *m/z* 271.1307 [M + Na]^+^ (calcd. for C_15_H_20_O_3_Na, 271.1305).

### 3.8 Preparation of ***1d*** and ***1e***

Compound **1** (30 mg) was hydrolyzed with 4 N HCl–dioxane (1:1, 15 mL) at 100 °C for 2 h. The reaction mixture was partitioned between chloroform and H_2_O three times. The organic layer was neutralized with 20 M Ag_2_CO_3_ and filtered. The resulting solution was washed with brine (50 mL), dried over Na_2_SO_4_, and then filtered and evaporated. The residue was subjected to column chromatography on silica gel using petroleum ether and ethyl acetate as eluent to yield **1d** (11 mg, 56%) and **1e** (7 mg, 38%) as a yellowish oil.

**1d**: amorphous powder; IR (KBr)ν_max_ 3402, 1765, 1076, 1025 cm^−1^; ^1^H-NMR (500 MHz, CDCl_3_) δ 5.74 (s, 1H, H-12), 4.64 (s, 1H, H-3), 4.23 (d, *J* = 13.0 Hz, 1H, H-14a), 4.12 (d, *J* = 13.0 Hz, 1H, H-14b), 1.79 (m, 1H, H-8), 1.25 (s, 3H, H-7), 0.99 (d, *J* = 6.5 Hz, 3H, H-10), 0.96 (d, *J* = 6.5 Hz, 3H, H-9); ^13^C-NMR (125 MHz, CDCl_3_) δ 178.1 (C-15), 144.9 (C-13), 130.2 (C-12), 79.6 (C-3), 50.9 (C-4), 46.4 (C-1), 44.7 (C-5), 43.8 (C-6), 40.8 (C-14), 34.0 (C-11), 30.8 (C-2), 29.1 (C-7), 25.8 (C-8), 20.6 (C-10), 19.8 (C-9); HRESIMS *m/z* 291.1128 [M + Na]^+^ (calcd. for C_15_H_21_O_2_ClNa, 291.1127).

**1e**: amorphous powder; IR (KBr)ν_max_ 3402, 1765, 1076, 1025 cm^−1^; ^1^H-NMR (500 MHz, CDCl_3_) δ 5.59 (s, 1H, H-12), 4.65 (s, 1H, H-3), 4.18 (s, 2H, H-14), 1.79 (m, 1H, H-8), 1.15 (s, 3H, H-7), 1.00 (d, *J* = 6.5 Hz, 3H, H-10), 0.97 (d, *J* = 6.5 Hz, 3H, H-9); ^13^C-NMR (125 MHz, CDCl_3_) δ 178.3 (C-15), 149.1 (C-13), 126.4 (C-12), 79.9 (C-3), 59.8 (C-14), 51.0 (C-4), 46.1 (C-1), 44.8 (C-5), 42.7 (C-6), 34.2 (C-11), 31.4 (C-2), 28.4 (C-7), 25.2 (C-8), 20.7 (C-10), 19.8 (C-9); HRESIMS *m/z* 273.1470 [M + Na]^+^ (calcd. for C_15_H_22_O_3_Na, 273.1471).

### 3.9. Neurite Outgrowth-Promoting Assay

Neurite outgrowth-promoting activity of these isolated compounds was assessed as described previously by our group [17,18]. Experiments were repeated at least three times, and data are expressed as mean ± SD (** *p* < 0.01). Statistical analyses were performed using Dunnett’s *t*-test.

## 4. Conclusions

In conclusion, the present study reported the identification of three new compounds, including two new picrotoxane sesquiterpene glycosides nepalactones A (**1**) and B (**2**) and one new coumarin nepalarin (**3**), from the root barks of *C.* *nepalensis*. In addition, five congeners **1a**–**e** were synthesized from nepalactone A. We for the first time discovered that these compounds, especially compound **1c** and **3**, could potentiate NGF-induced neurite outgrowth in PC12 cells.

## Figures and Tables

**Table 1:** ^1^H-NMR (500 MHz) and ^13^C-NMR (125 MHz) data for **1**, **1a**, **1b**, and **2** (δ ppm).

No.	1 ^a^		1a ^b^		2 ^a^	
	δ_H_ (*J* in Hz)	δ_C_	δ_H_ (*J* in Hz)	δ_C_	δ_H_ (*J* in Hz)	δ_C_
1		42.5		46.1		42.5
2α	1.74 (d, 15.5)	34.0	1.63 (d, 15.0)	33.6	1.64 (d, 15.5)	33.3
2β	2.45 (dd, 2.5, 15.5)		2.40 (dd, 3.5, 15.0)		2.46 (dd, 3.5, 15.5)	
3	4.72 (m)	80.2	4.63 (m)	79.6	4.68 (m)	79.7
4	2.02 (m)	51.5	1.96 (m)	51.5	2.02 (m)	51.4
5	2.35 (m)	45.5	2.27 (m)	45.5	2.50 (m)	45.0
6	2.36 (m)	41.7	2.26 (m)	41.3	2.39 (d, 3.0)	49.1
7	1.25 (s)	31.9	1.15 (s)	31.7	1.28 (s)	32.7
8	1.83 (m)	25.0	1.76 (m)	24.8	1.81 (m)	25.3
9	0.99 (d, 6.0)	19.3	0.96 (d, 6.0)	19.6	0.99 (d, 6.5)	19.8
10	1.01 (d, 6.0)	20.0	0.98 (d, 6.0)	20.7	1.03(d, 6.5)	20.9
11α	2.31 (m)	34.2	2.31 (m)	34.7	4.45 (m)	81.9
11β	2.23 (m)		2.02 (m)			
12α	4.90 (t, 7.5)	79.4	4.77 (t, 7.4)	81.7	2.75 (m)	39.4
12β					2.92 (dd, 8.0, 18.5)	
13		158.2		156.4		156.3
14a	5.21 (d, 1.5)	111.5	5.19 (d, 1.5)	112.9	4.91 (d, 8.0)	106.1
14b	5.42 (d, 1.5)		5.44 (d, 1.5)		4.88 (d, 8.0)	
15		180.6		179.0		178.6
1′	4.35 (d, 7.5)	101.5	4.56 (d, 8.0)	100.4	4.33 (d, 7.5)	102.4
2′	3.22 (m)	73.5	4.96 (m)	75.1	3.27 (s)	73.5
3′	3.40 (m)	76.6	5.18 (t, 10.0)	73.2	3.44 (m)	76.4
4′	3.39 (m)	70.2	5.05 (t, 10.0)	68.5	3.44 (m)	69.7
5′	3.29 (m)	76.2	3.68 (m)	71.9	3.29 (m)	75.7
6′a	3.87 (dd, 2.5, 12.0)	61.6	4.26 (dd, 5.0, 12.0)	62.2	3.87 (dd, 3.0,12.0)	61.6
6′b	3.75 (dd, 5.5, 12.0)		4.12 (dd, 2.0, 12.0)		3.78 (dd, 3.0,12.0)	
2′-Ac			2.03 (s)	169.0/20.7		
3′-Ac			1.99 (s)	170.8/20.8		
4′-Ac			2.01 (s)	169.3/20.7		
6′-Ac			2.08 (s)	170.6/20.6		

^a,b^: Data were recorded in MeOH-*d*_4_, and CDCl_3_, respectively.

## Supplementary Materials

Supplementary materials can be accessed at: http://www.mdpi.com/1420-3049/21/10/1344/s1.
