# Supplementary material for: Picrotoxane Sesquiterpene Glycosides and a Coumarin Derivative from Coriaria nepalensis and Their Neurotrophic Activity
Source: Molecules. 2016 Oct 12;21(10):1344. doi: 10.3390/molecules21101344 (PMC6273530; doi:10.3390/molecules21101344)
Supplement: Supplementary file 1 [file molecules-21-01344-s001.pdf]

Supplementary Meterial: Picrotoxane Sesquiterpene Glycosides and Coumarin Derivative from *Coriaria nepalensis* and Their Neurotrophic Activity

Yuan-Yuan Wang, Jun-MianTian, Cheng-Chen Zhang, Bo Luo, and Jin-Ming Gao *

Figure S1. ^{1}H-NMR spectra of compound 1 in CD_3OD 2
Figure S2. ^{13}C-NMR spectra of compound 1 in CD_3OD 4
Figure S3. HSQC spectra of compound **1** in CD_3OD 5
Figure S4. ^{1}H-^{1}H COSY spectra of compound **1** in CD_3OD 6
Figure S5. HMBC spectra of compound **1** in CD_3OD 7
Figure S6. NOESY spectra of compound **1** in CD_3OD 8
Figure S7. HRESIMS spectrum of compound **1** 9
Figure S8. IR spectra of compound **1** 10
Figure S9. UV spectra of compound **1** 11
Figure S10. ^{1}H-NMR spectra of compound **1a** in $CDCl_3$ 12
Figure S11. ^{13}C-NMR spectra of compound **1a** in $CDCl_3$ 13
Figure S12. HSQC spectra of compound **1a** in $CDCl_3$ 13
Figure S13.^{1}H-^{1}H COSY spectra of compound **1a** in $CDCl_3$ 15
Figure S14. HMBC spectra of compound **1a** in $CDCl_3$ 16
Figure S15. NOESY spectra of compound **1a** in $CDCl_3$ 17
Figure S16. HRESIMS spectrum of compound **1a** 18
Figure S17. IR spectra of compound **1a** 19
Figure S18. UV spectra of compound **1a** 20
Figure S19. ^{1}H-NMR spectra of compound **2** in CD_3OD 21
Figure S20. ^{13}C-NMR spectra of compound **2** in CD_3OD 22
Finger S21. HSQC spectra of compound **2** in CD_3OD 23
Finger S22. ^{1}H-^{1}H COSY spectra of compound **2** in CD_3OD 24
Figure S23. HMBC spectra of compound **2** in CD_3OD 25
Figure S24. NOESY spectra of compound **2** in CD_3OD 26

Figure S25. HRESIMS spectrum of compound **2**....27
Figure S26. IR spectra of compound **2** 28
Figure S27. UV spectra of compound **2** 29
Figure S28. ^{1}H-NMR spectra of compound **3** in $CDCl_3$ 30
Figure S29. ^{13}C-NMR spectra of compound **3** in $CDCl_3$ 31
Finger S30. HSQC spectra of compound **3** in $CDCl_3$ 32
Finger S31. ^{1}H-^{1}H COSY spectra of compound **3** in $CDCl_3$....33
Finger S32. HMBC spectra of compound **3** in $CDCl_3$ 34
Figure S33. HRESTMS of compound **3** in CD_3OD 35
Figure S34. IR spectra of compound **3** 36
Figure S35. UV spectra of compound **3** 37
Figure S36. ^{1}H-NMR spectra of compound 1b in $CDCl_3$....38
Figure S37. ^{1}H-NMR spectra of compound **1c** in $CDCl_3$ 39
Figure S38. ^{13}C-NMR spectra of compound **1c** in $CDCl_3$....40
Finger S39. HSQC spectra of compound **1c** in $CDCl_3$....41
Finger S40. ^{1}H-^{1}H COSY spectra of compound **1c** in $CDCl_3$....42
Figure S41. HMBC spectra of compound **1c** in $CDCl_3$....43
Figure S42. NOESY spectra of compound **1c** in $CDCl_3$ 44
Figure S43. HRESIMS spectrum of compound **1c.**....45
Figure S44. ^{1}H-NMR spectra of compound **1d** in $CDCl_3$....46
Figure S45. ^{13}C-NMR spectra of compound **1d** in $CDCl_3$....47
Figure S46. HRESIMS spectrum of compound **1d** 48
Figure S47. ^{1}H-NMR spectra of compound **1e** in $CDCl_3$ 49
Figure S48. ^{13}C-NMR spectra of compound **1e** in $CDCl_3$ 50
Figure S49. HRESIMS spectrum of compound **1e**....51

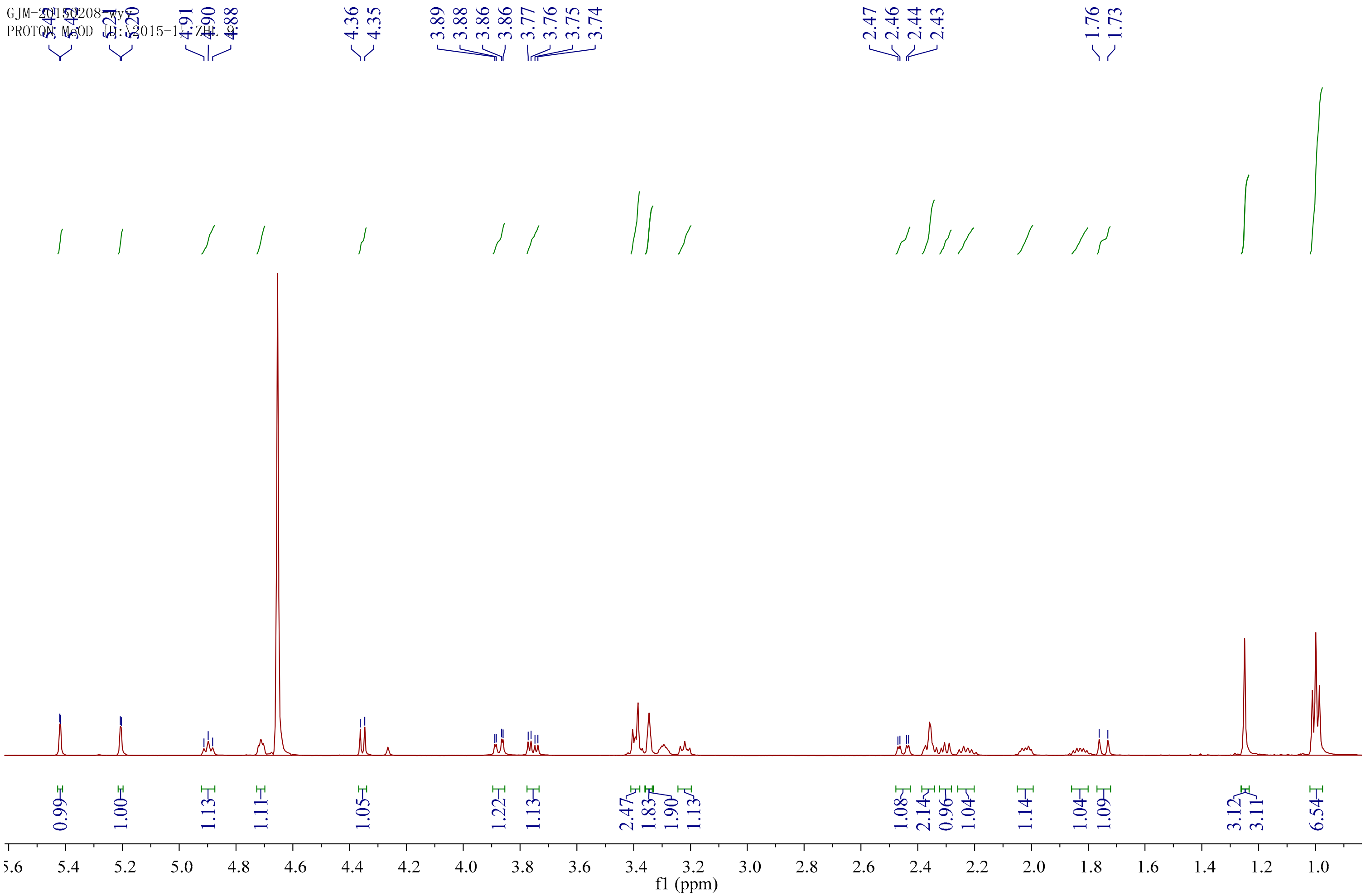

Figure S1. ^{1}H-NMR spectra of compound **1** in CD_3OD.

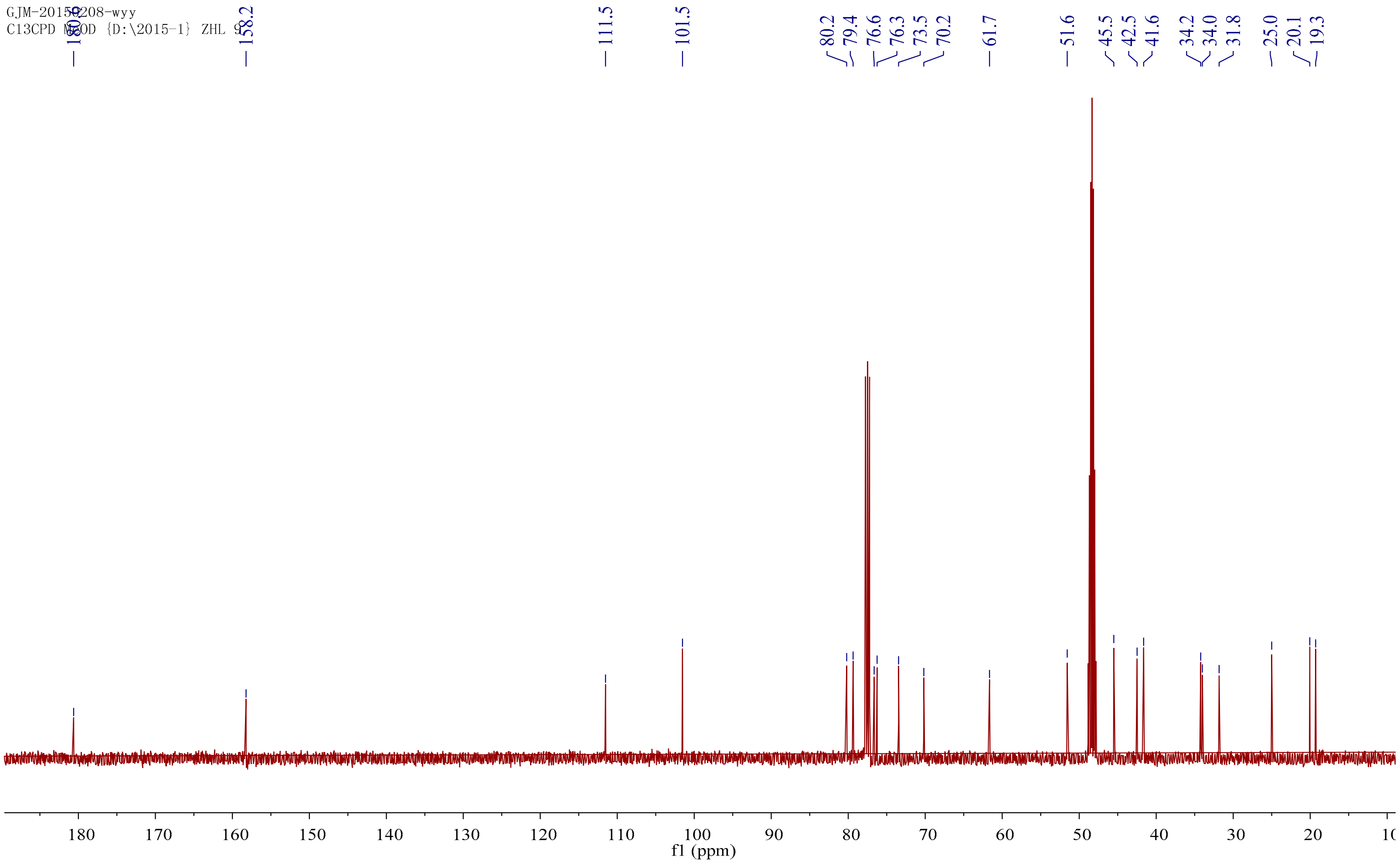

Figure S2. ^{13}C-NMR spectra of compound **1** in CD_3OD.

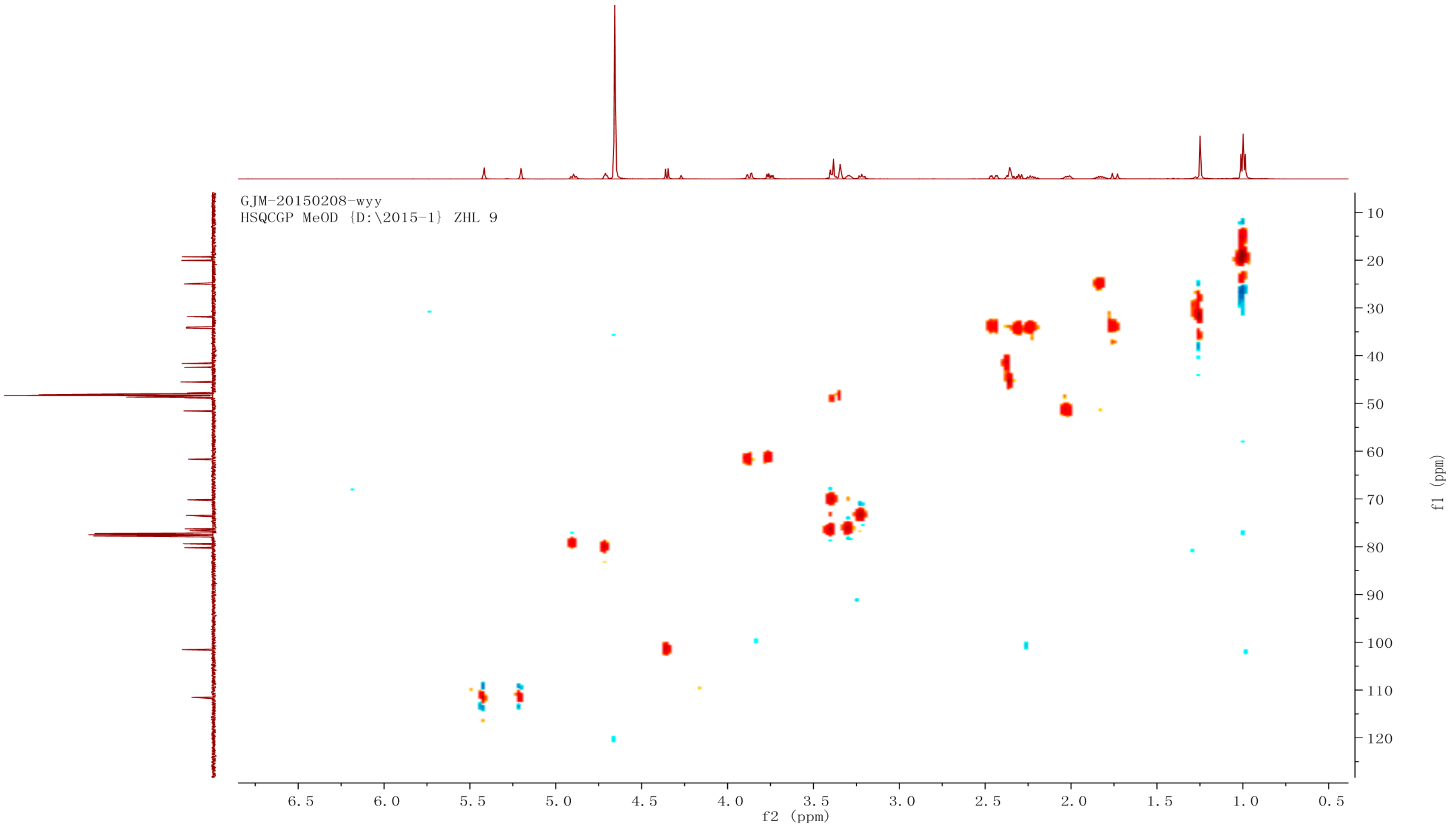

Figure S3. HSQC spectra of compound **1** in CD_3OD.

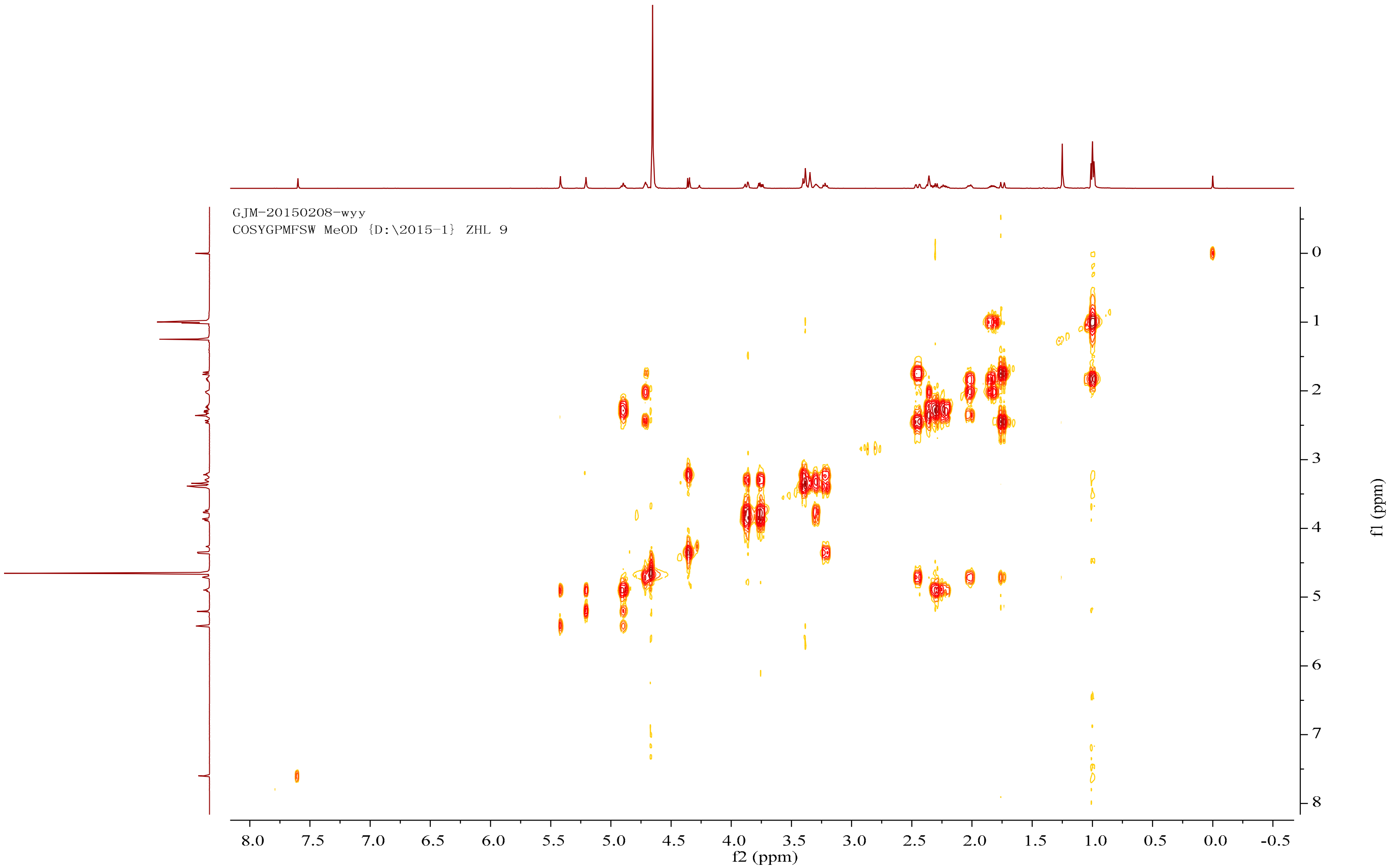

Figure S4. ^{1}H-^{1}H COSY spectra of compound **1** in CD_3OD.

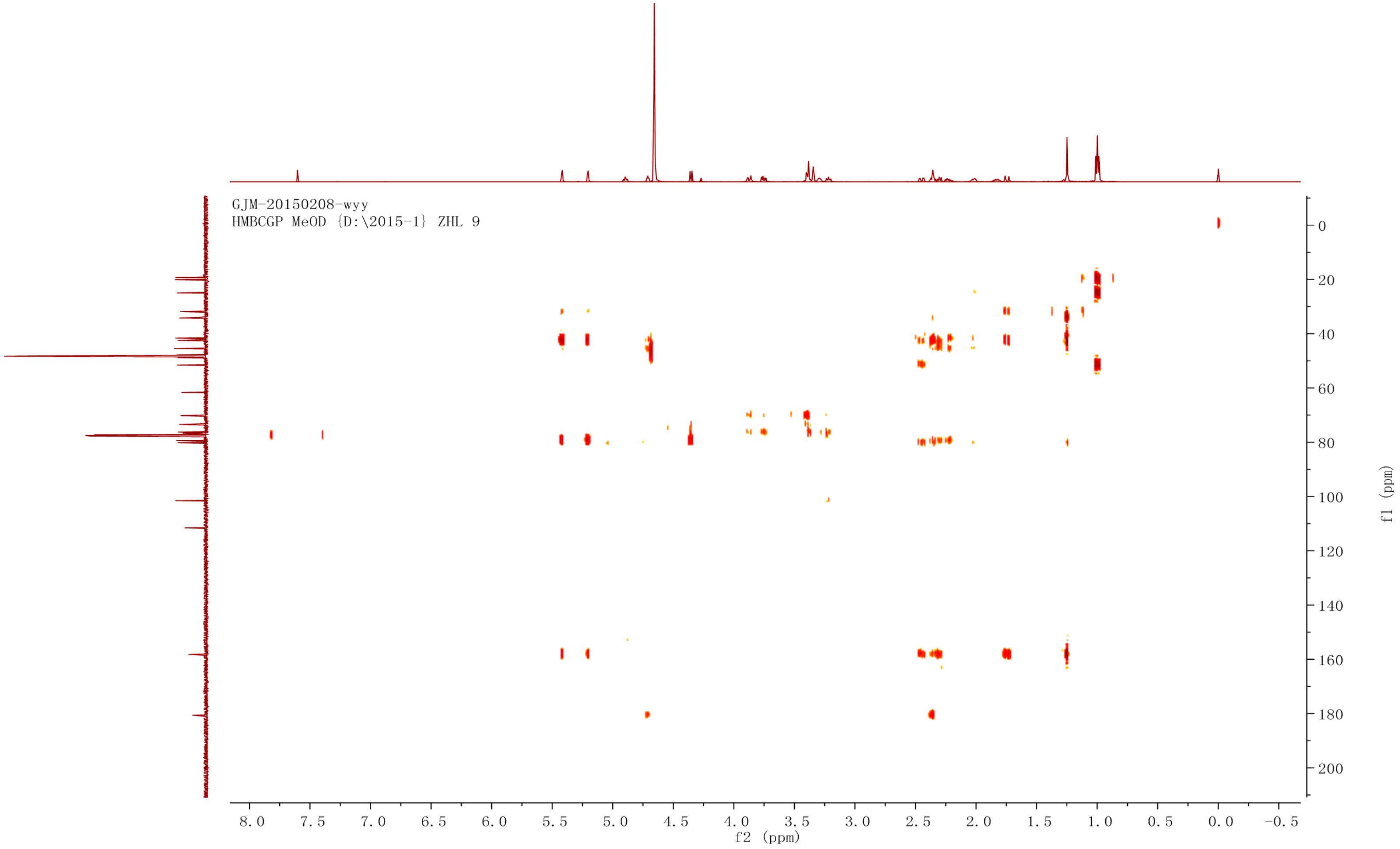

Figure S5. HMBC spectra of compound **1** in CD_3OD.

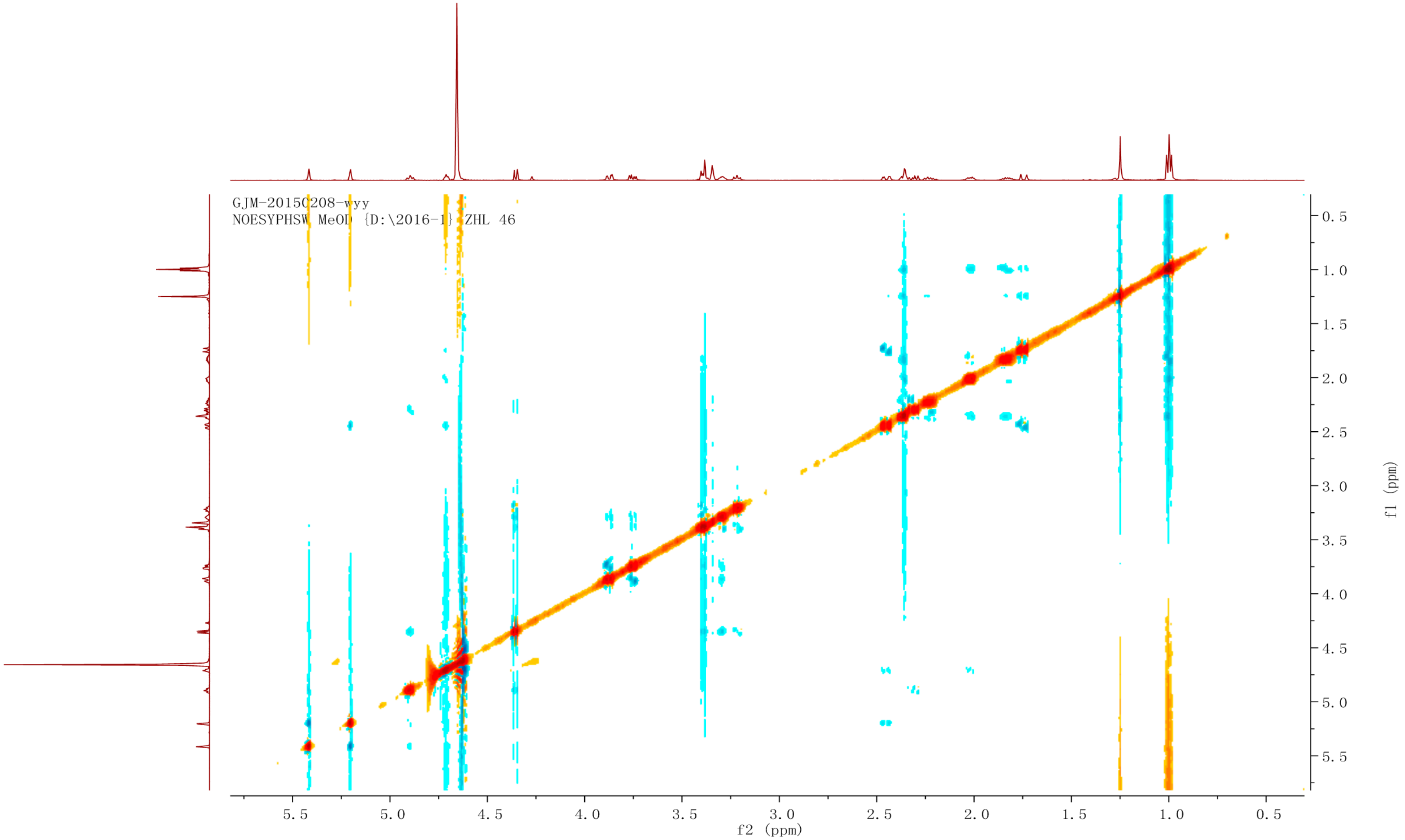

Figure S6. NOESY spectra of compound **1** in CD3OD.

Elemental Composition Report **Page 1**

Single Mass Analysis
Tolerance = 5.0 PPM / DBE: min = -1.5, max = 50.0
Element prediction: Off
Number of isotope peaks used for i-FIT = 3

Monoisotopic Mass, Even Electron Ions
161 formula(e) evaluated with 1 results within limits (up to 50 closest results for each mass)
Elements Used:
C: 5-80 H: 2-120 O: 0-20 Na: 0-1

wyy-1 LCT PXE KE324 14-Oct-2015
13:39:31
wyy-1_1014 27 (0.583) AM2 (Ar,13500.0,0.00,0.70); ABS; Cm (17:28) 1: TOF MS ES+
1.41e+004

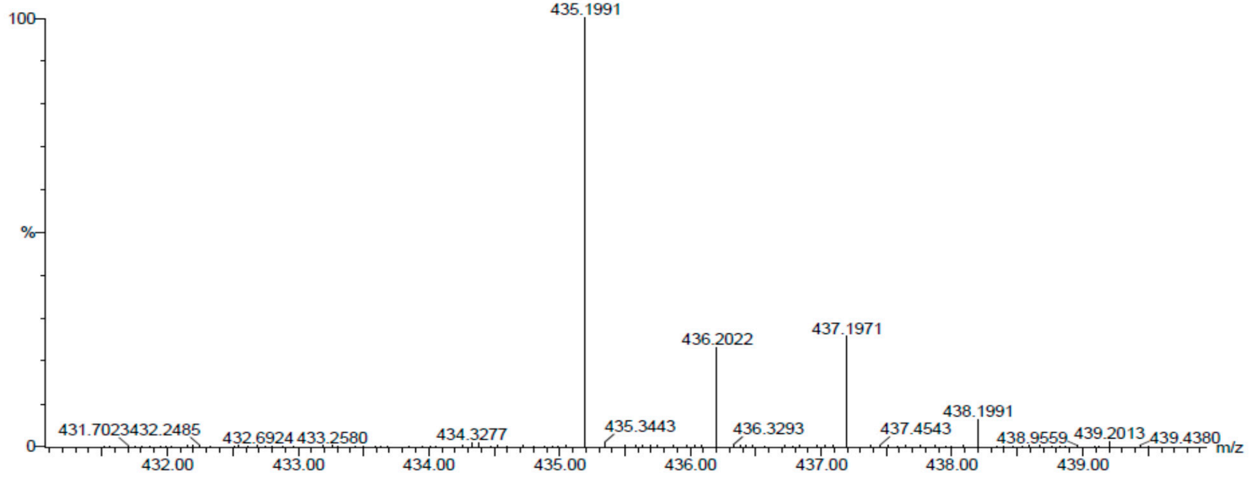

Minimum:				-1.5			
Maximum:		5.0	5.0	50.0			
Mass	Calc. Mass	mDa	PPM	DBE	i-FIT	i-FIT (Norm)	Formula
435.1991	435.1995	-0.4	-0.9	5.5	172.3	0.0	C21 H32 O8 Na

Figure S7. HRESIMS spectrum of compound **1.**

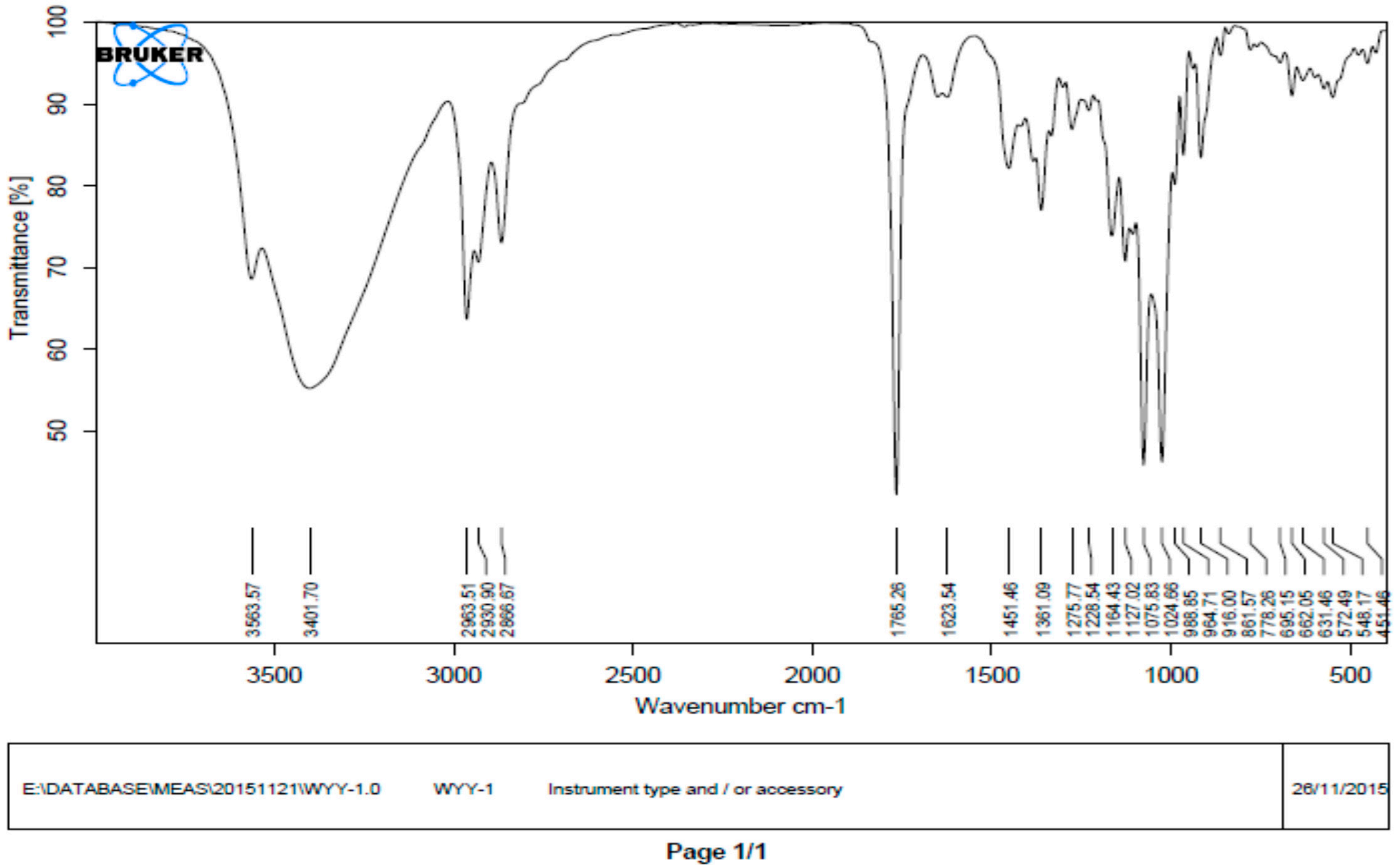

Figure S8. IR spectra of compound **1.**

THERMO ELECTRON ~ VISIONpro SOFTWARE V4.10

Operator Name	(None Entered)	Date of Report	2015-9-16
Department	(None Entered)	Time of Report	22:33:37下午
Organization	(None Entered)		
Information	(None Entered)		

Scan Graph

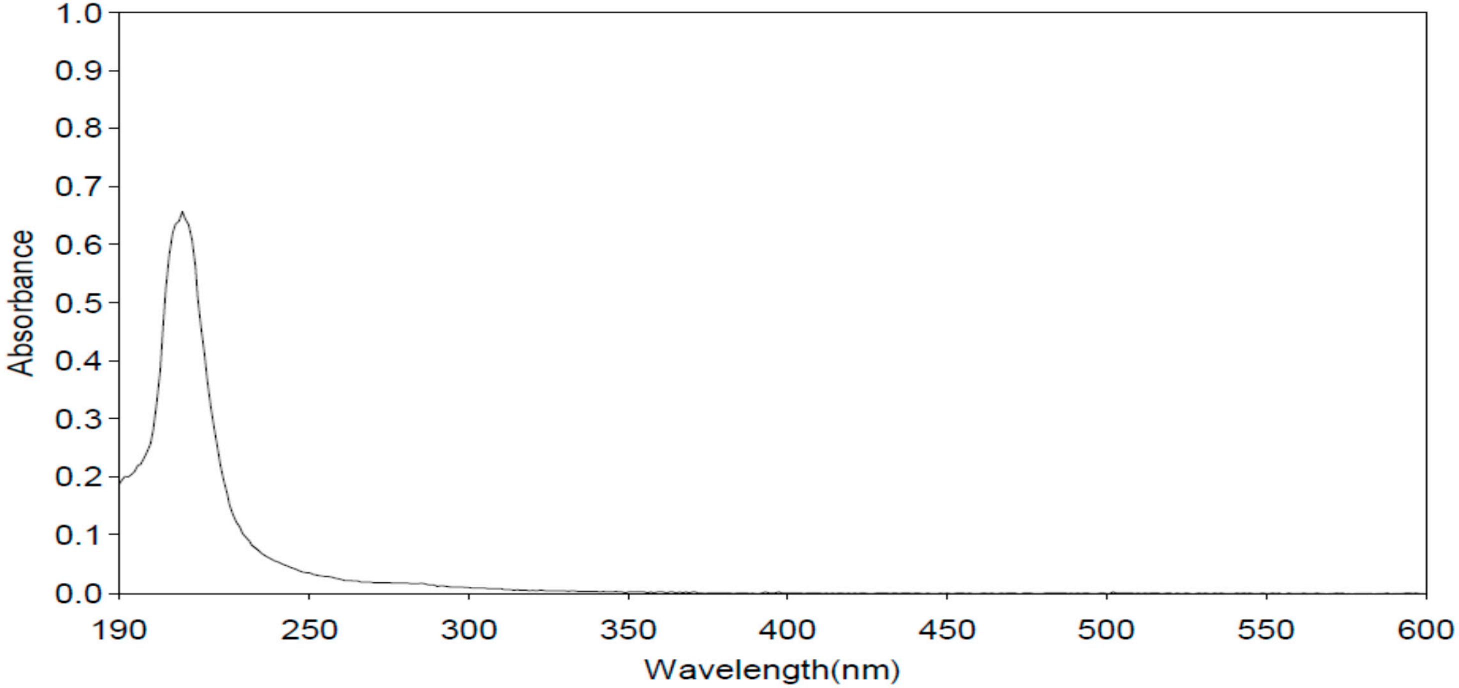

Figure S9. UV spectra of compound **1.**

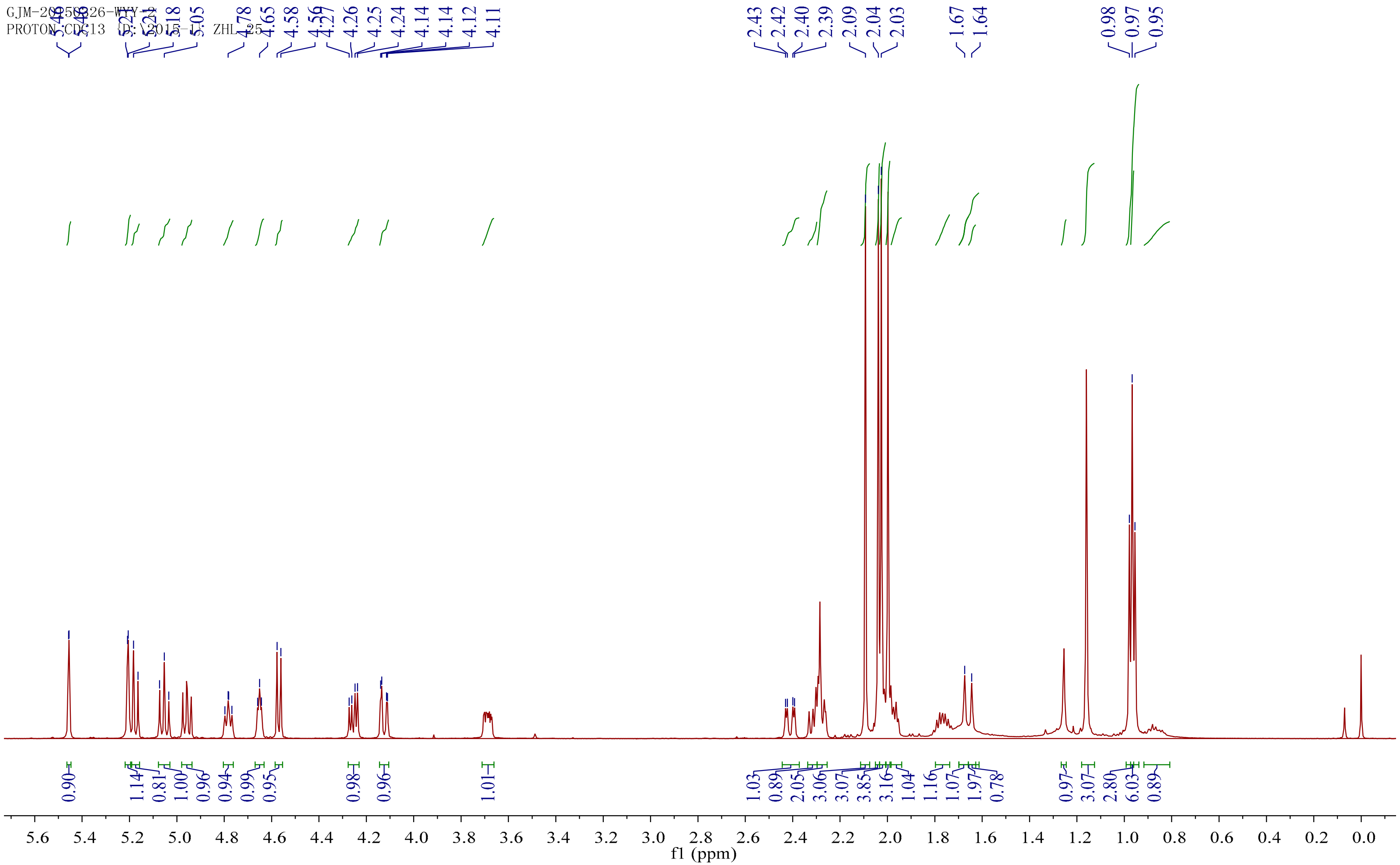

Figure S10. ^{1}H-NMR spectra of compound **1a** in $CDCl_3$.

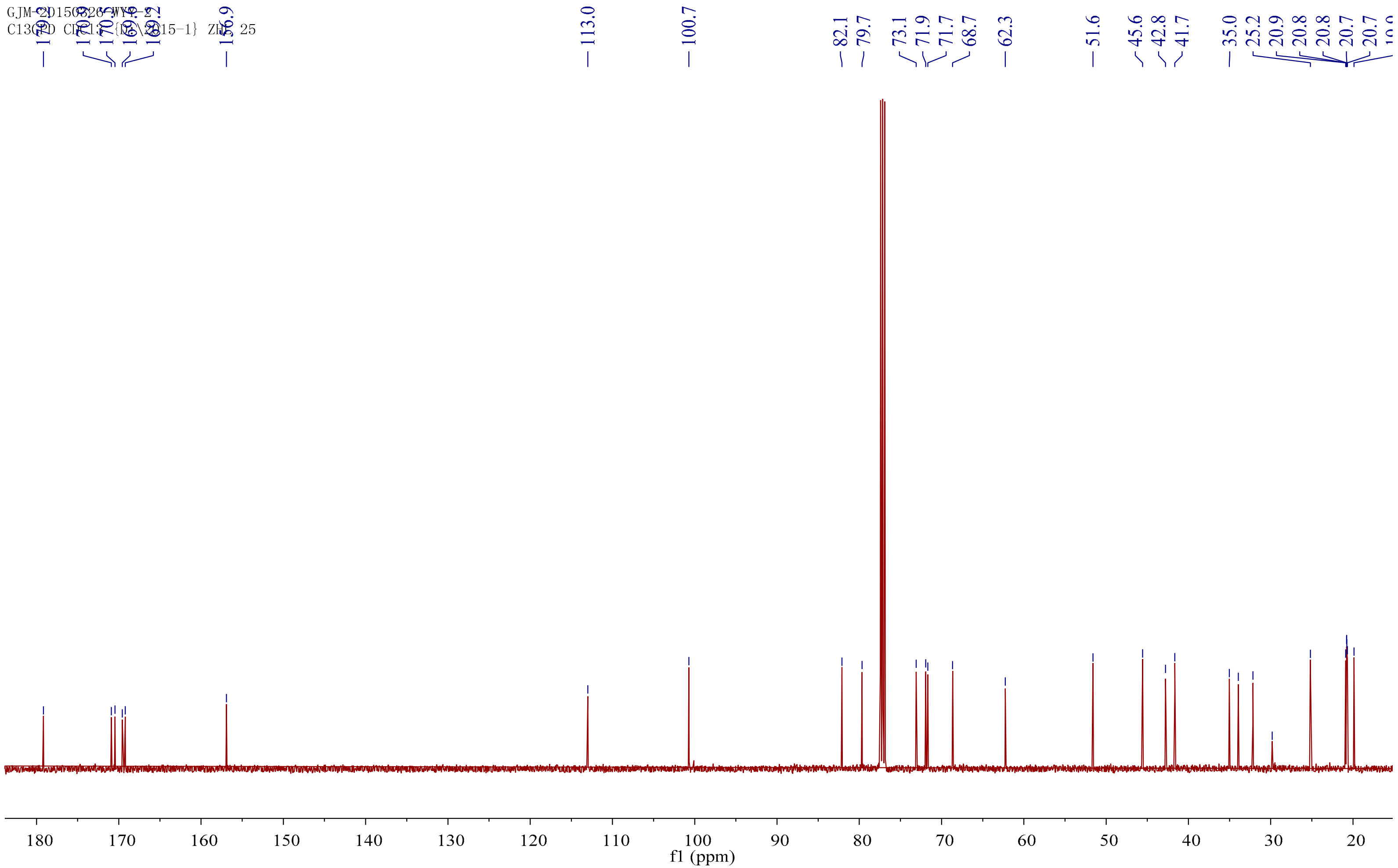

Figure S11. ^{13}C-NMR spectra of compound **1a** in $CDCl_3$.

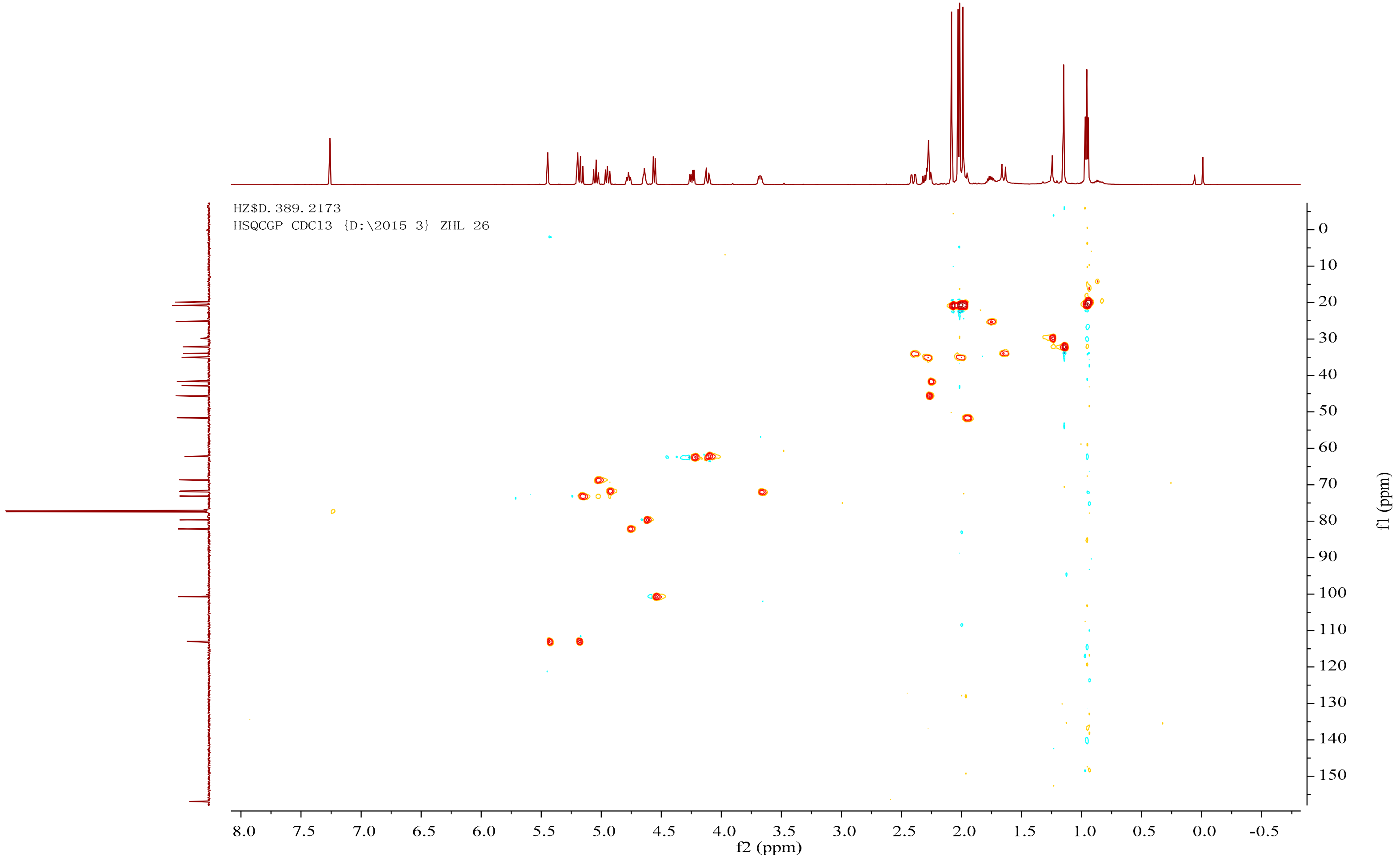

Figure S12. HSQC spectra of compound **1a** in $CDCl_3$.

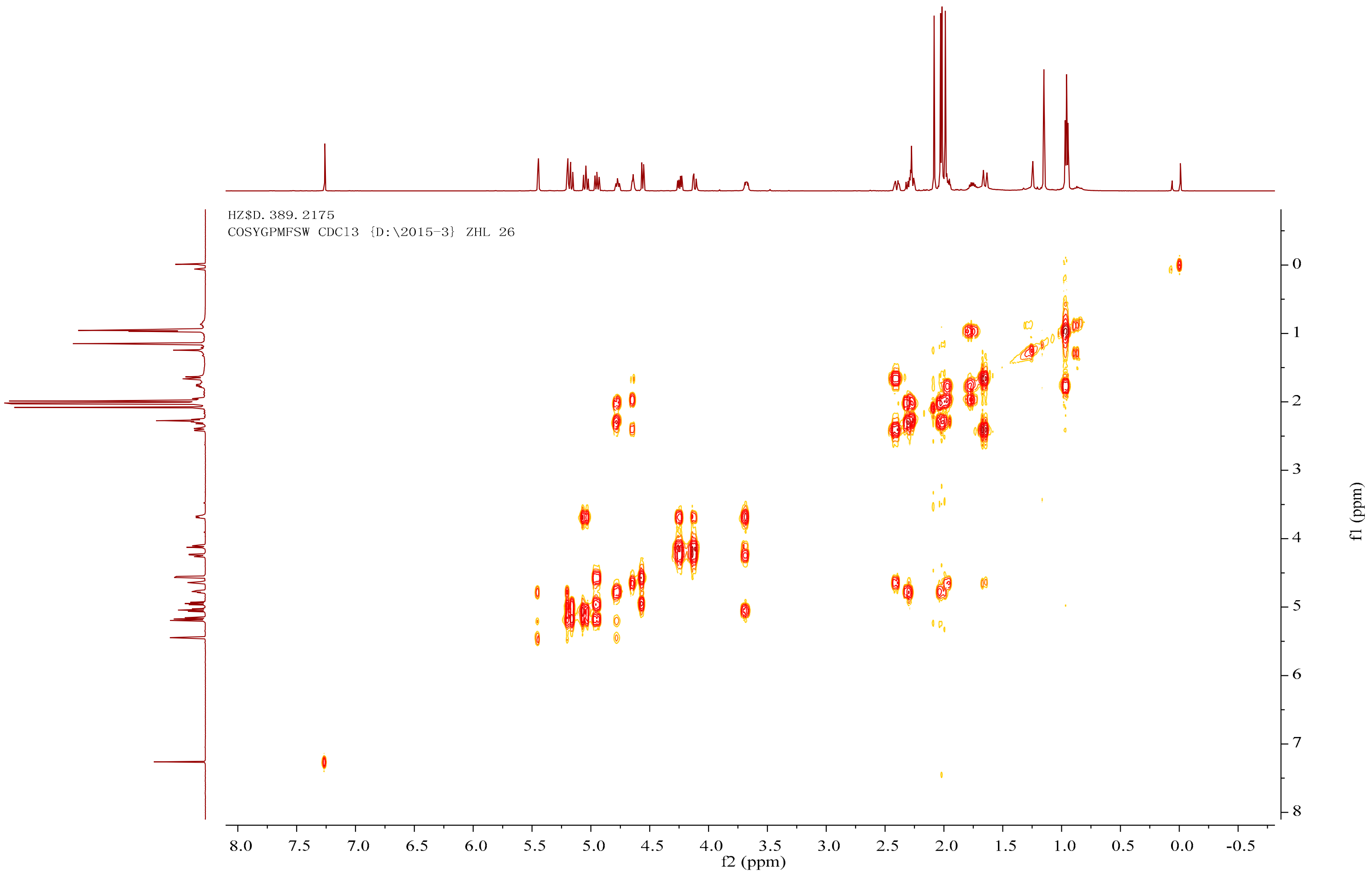

Figure S13.^{1}H-^{1}H COSY spectra of compound **1a** in $CDCl_3$.

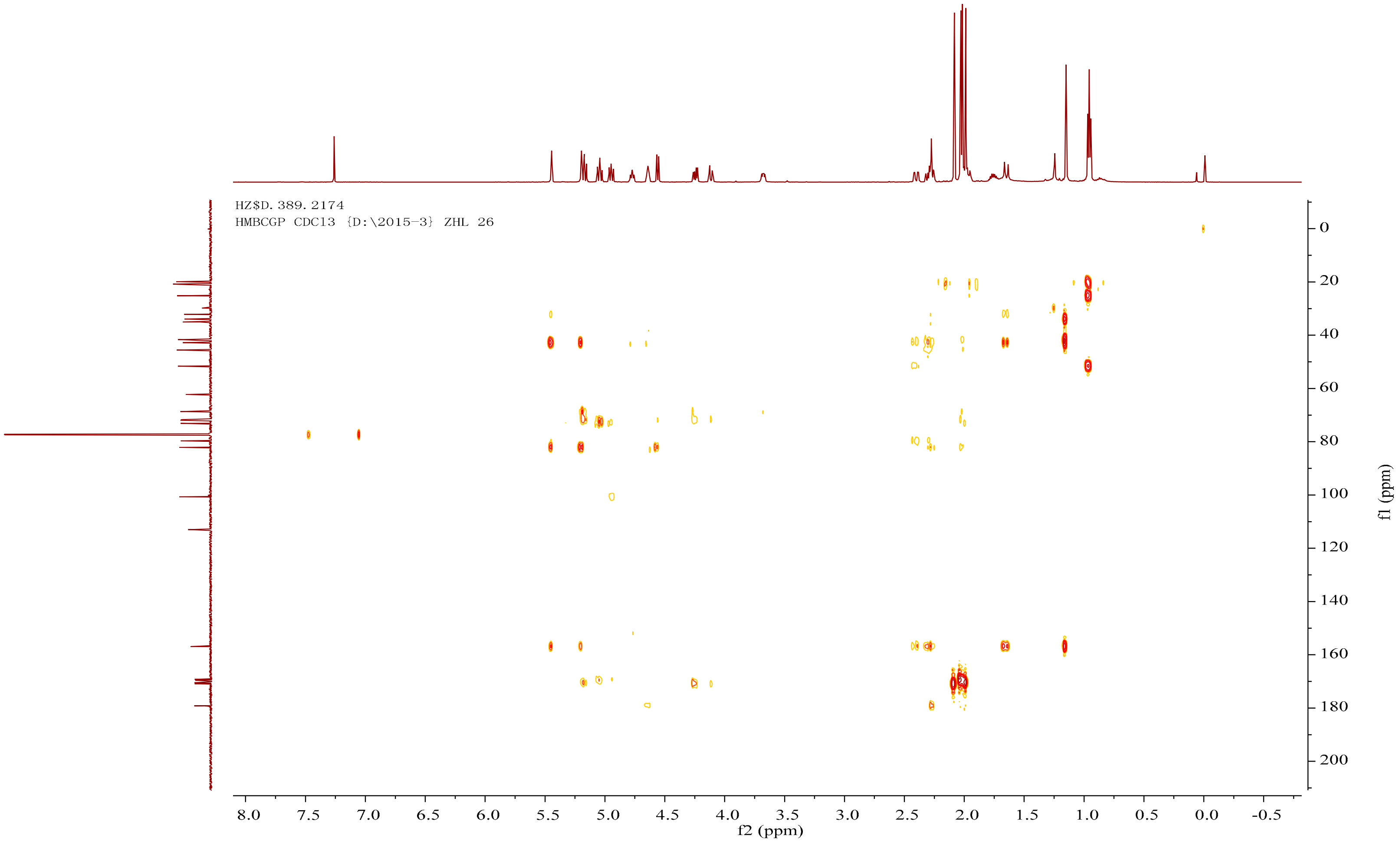

Figure S14. HMBC spectra of compound **1a** in $CDCl_3$.

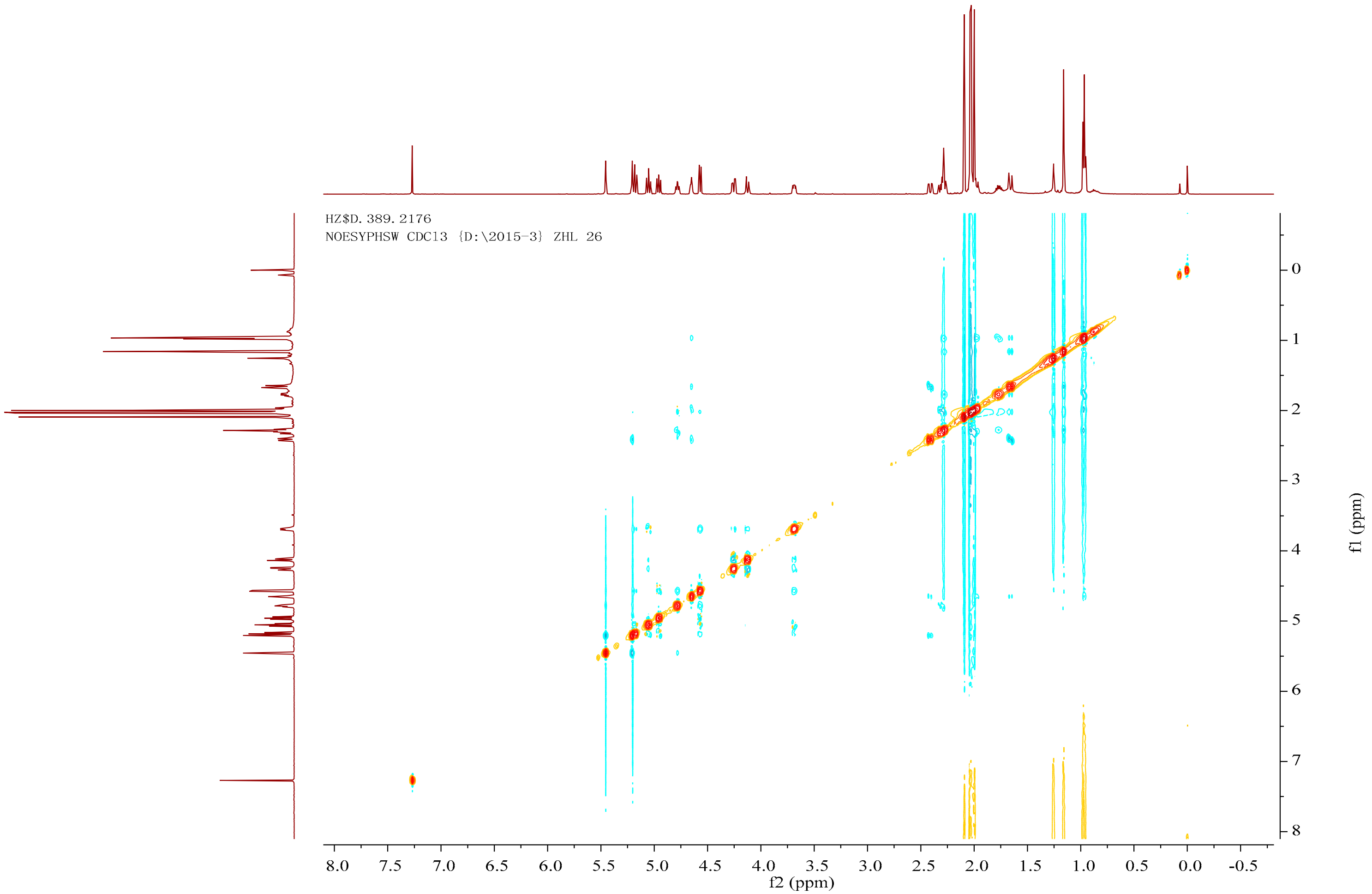

Figure S15. NOESY spectra of compound **1a** in $CDCl_3$.

Elemental Composition Report **Page 1**

Single Mass Analysis
Tolerance = 4.0 PPM / DBE: min = -1.5, max = 50.0
Element prediction: Off
Number of isotope peaks used for i-FIT = 3

Monoisotopic Mass, Even Electron Ions
245 formula(e) evaluated with 1 results within limits (up to 50 closest results for each mass)
Elements Used:
C: 5-80 H: 2-120 O: 0-20 Na: 0-1

wyy-2 LCT PXE KE324 14-Oct-2015 13:45:16
wyy-2_1014 20 (0.424) AM2 (Ar,10000.0,0.00,1.00); ABS; Cm (9:23) 1: TOF MS ES+ 9.22e+003

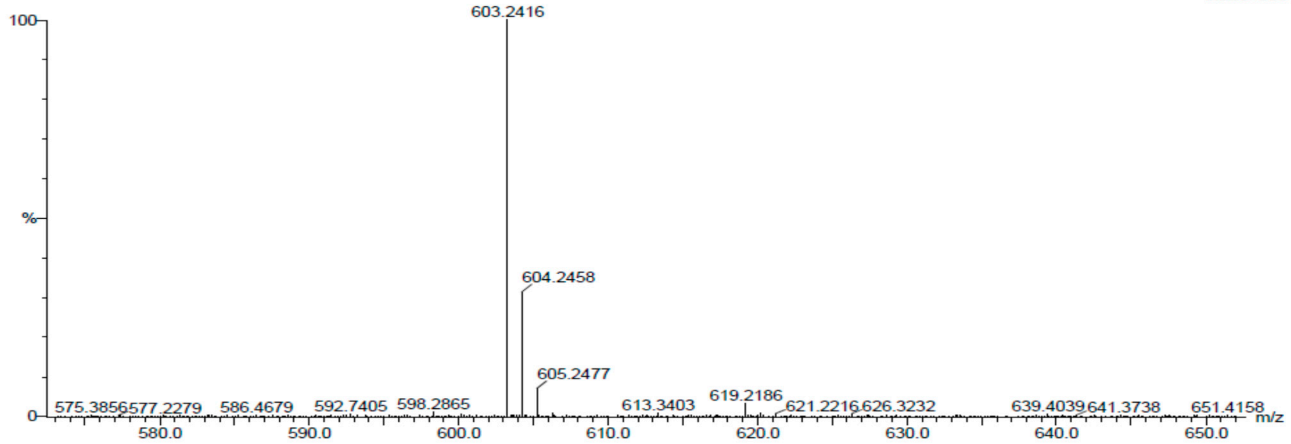

Minimum:				-1.5			
Maximum:		5.0	4.0	50.0			
Mass	Calc. Mass	mDa	PPM	DBE	i-FIT	i-FIT (Norm)	Formula
603.2416	603.2417	-0.1	-0.2	9.5	63.4	0.0	C29 H40 O12 Na

Figure S16. HRESIMS spectrum of compound **1a.**

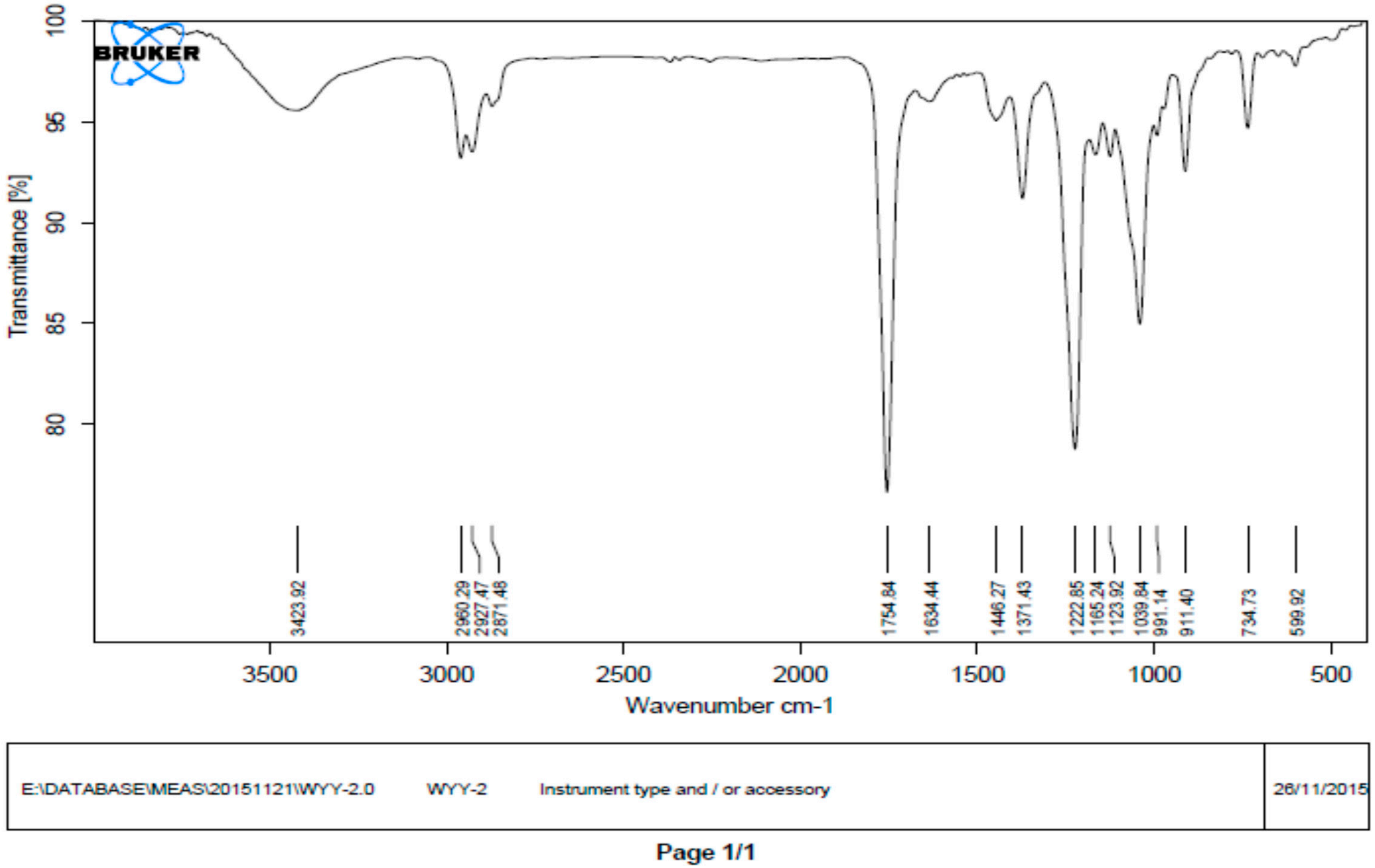

Figure S17. IR spectra of compound **1a.**

THERMO ELECTRON ~ VISIONpro SOFTWARE V4.10

Operator Name	(None Entered)	Date of Report	2015-9-16
Department	(None Entered)	Time of Report	22:39:59下午
Organization	(None Entered)		
Information	(None Entered)		

Scan Graph

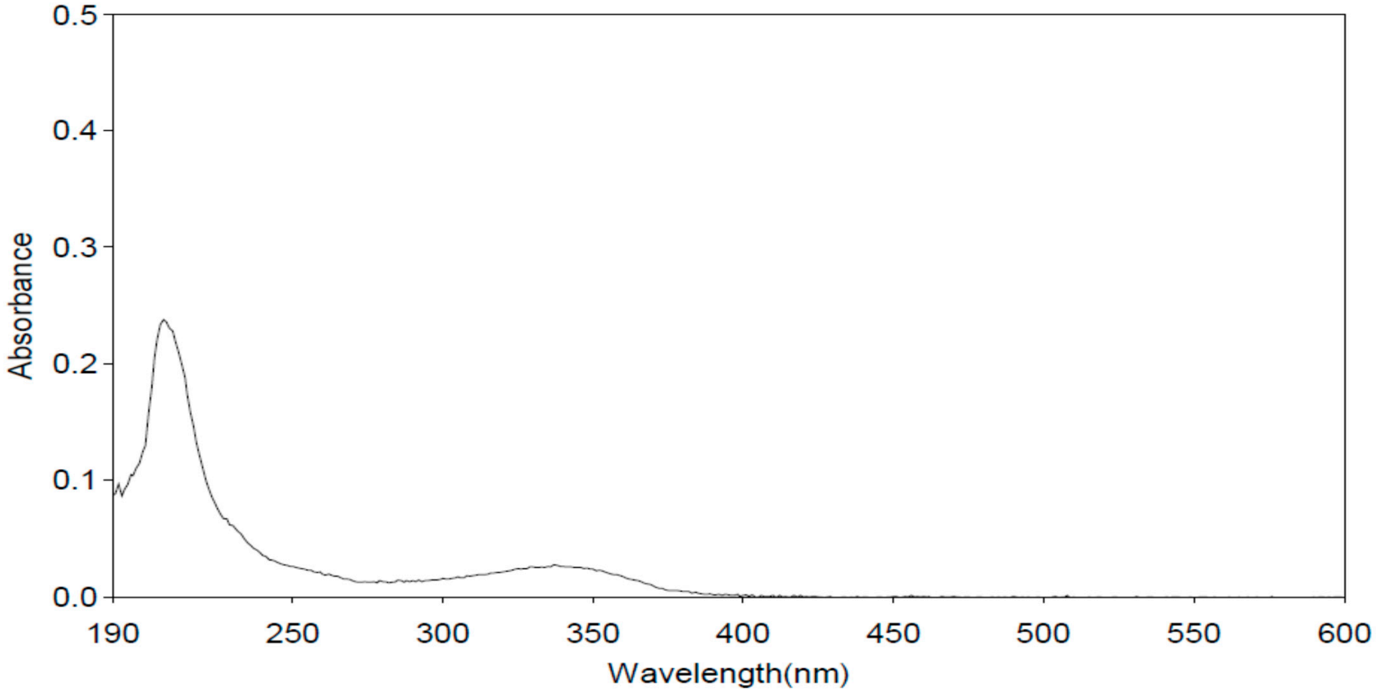

Figure S18. UV spectra of compound **1a.**

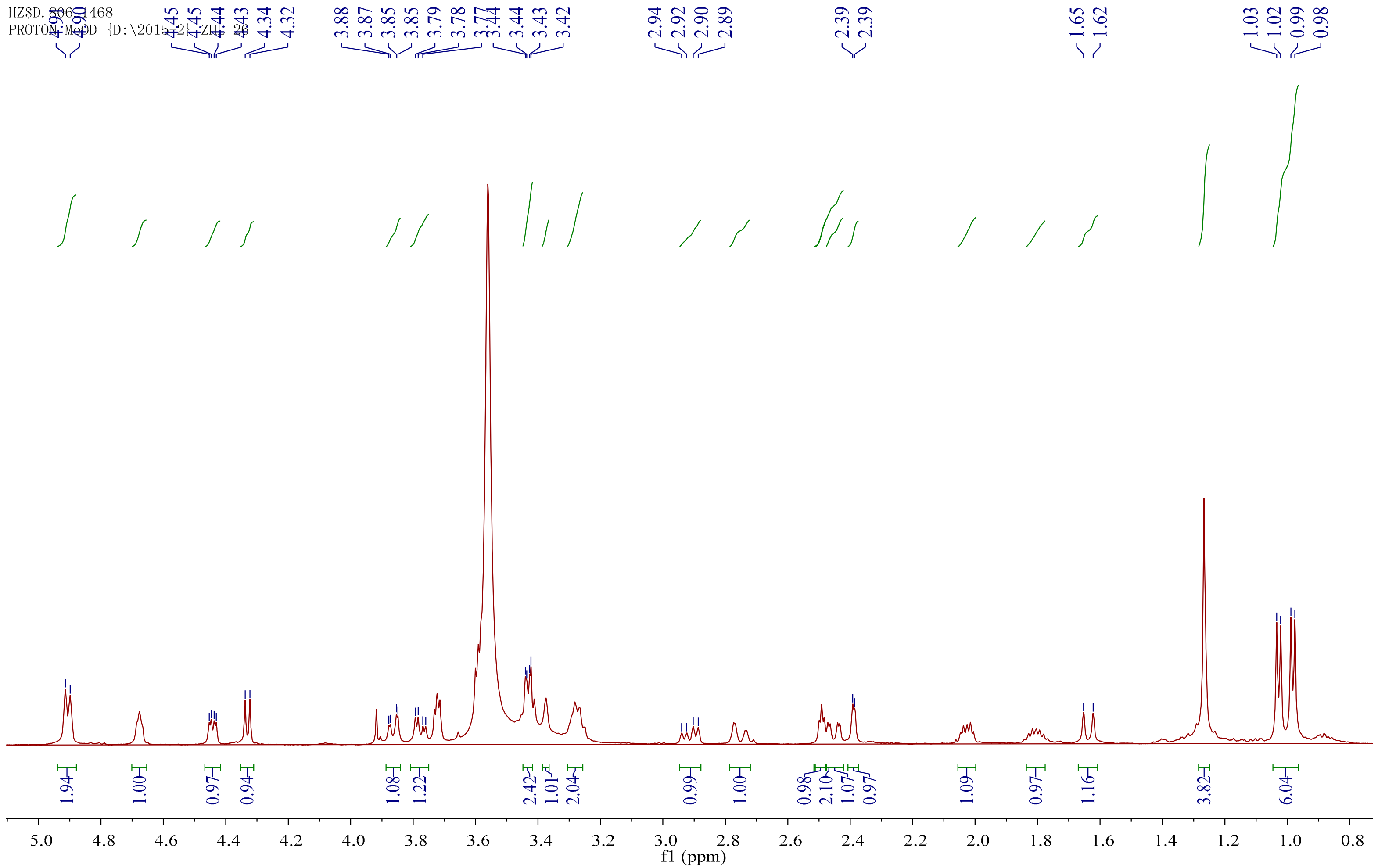

Figure S19. ^{1}H-NMR spectra of compound **2** in CD_3OD.

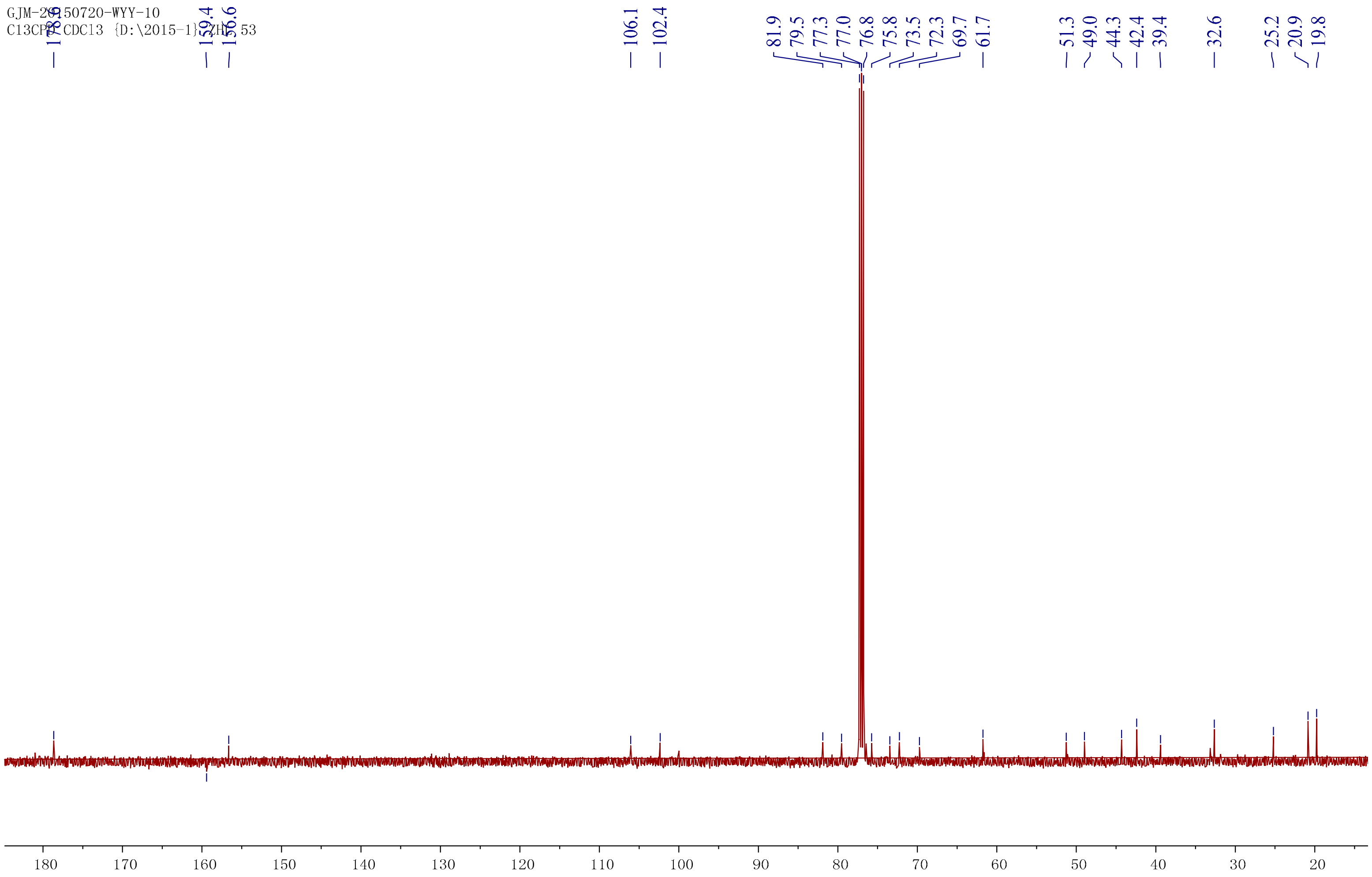

Figure S20. ^{13}C-NMR spectra of compound **2** in CD_3OD.

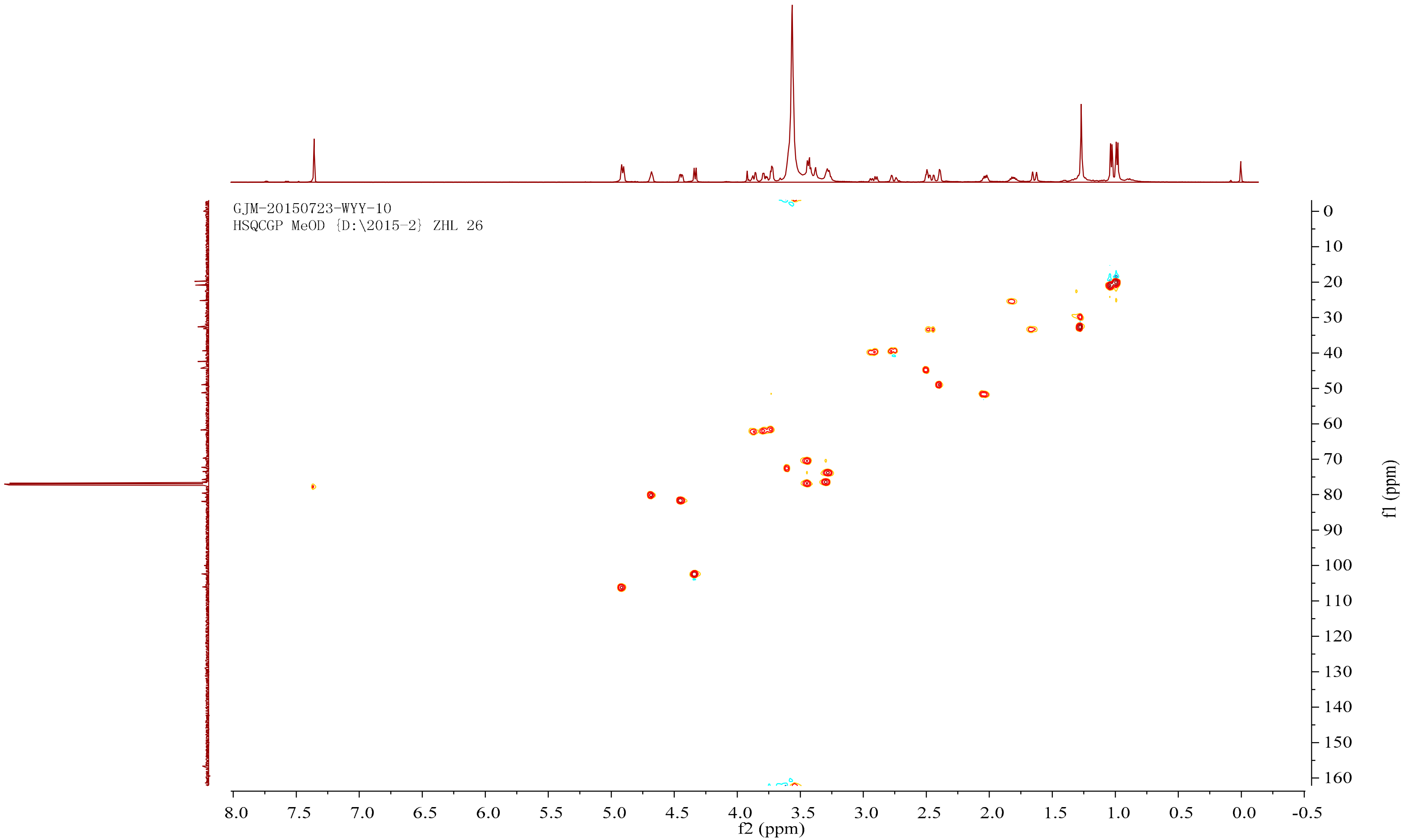

Finger S21. HSQC spectra of compound **2** in CD_3OD.

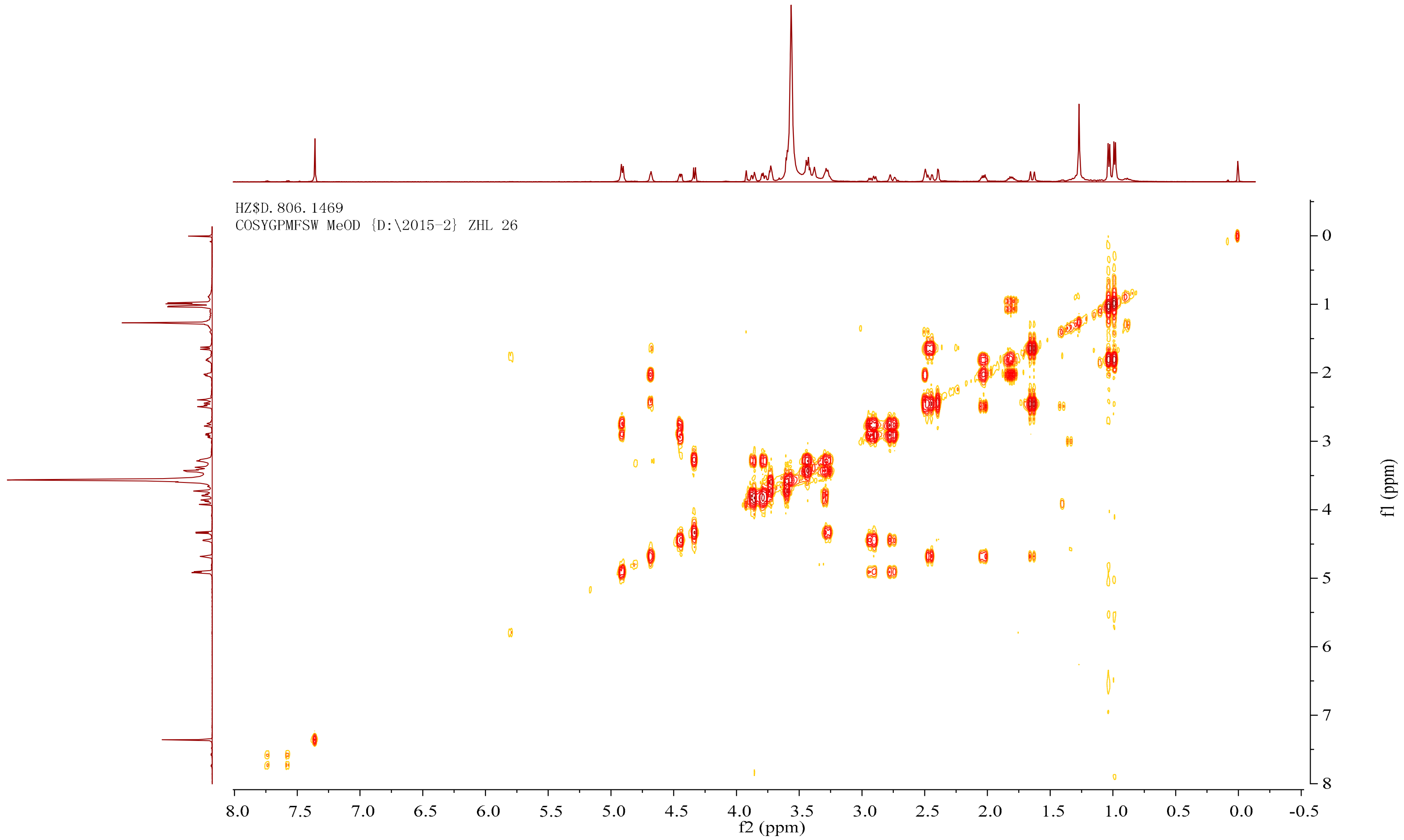

Finger S22. ^{1}H-^{1}H COSY spectra of compound **2** in CD_3OD.

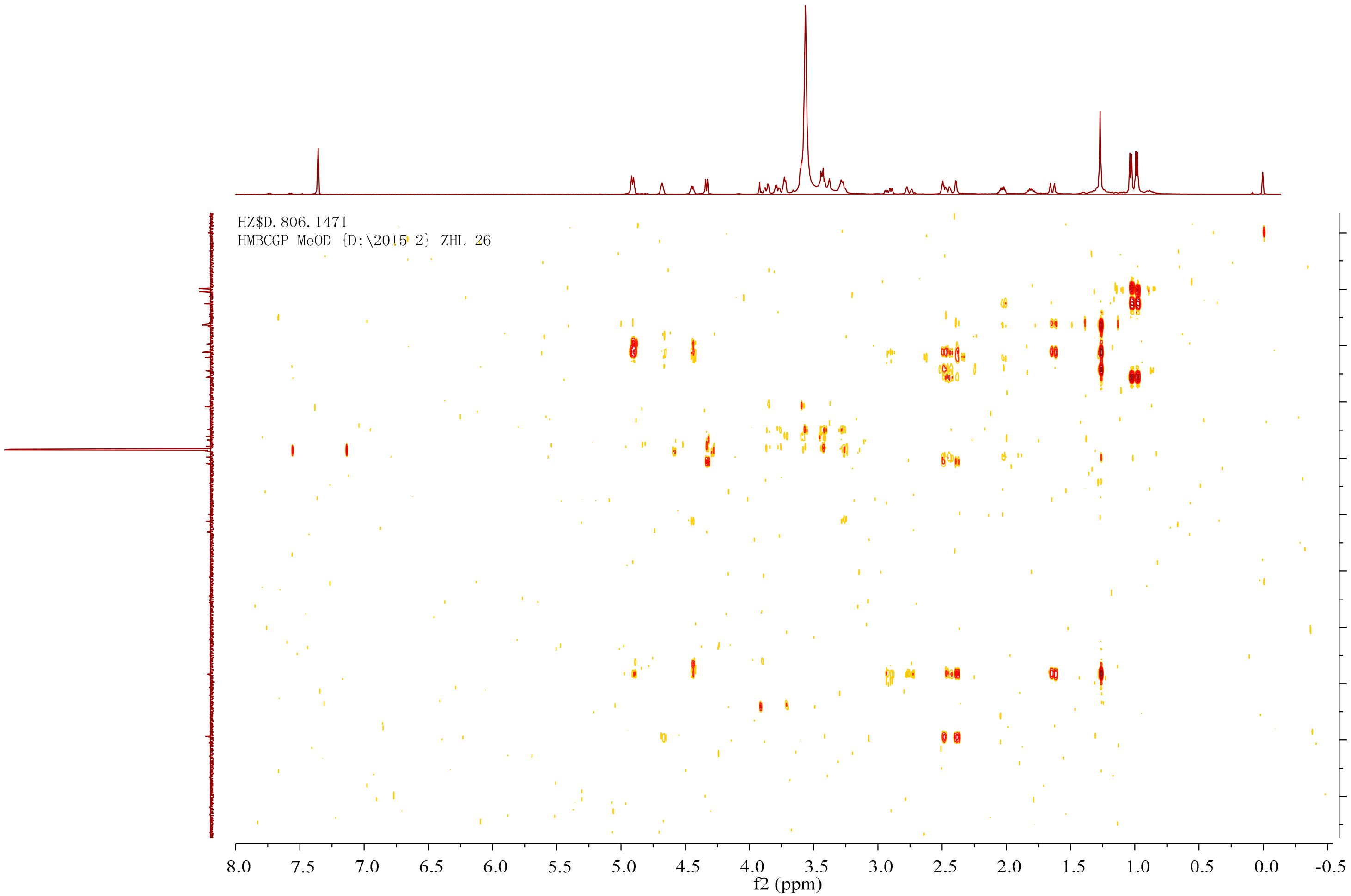

Figure S23. HMBC spectra of compound **2** in CD_3OD.

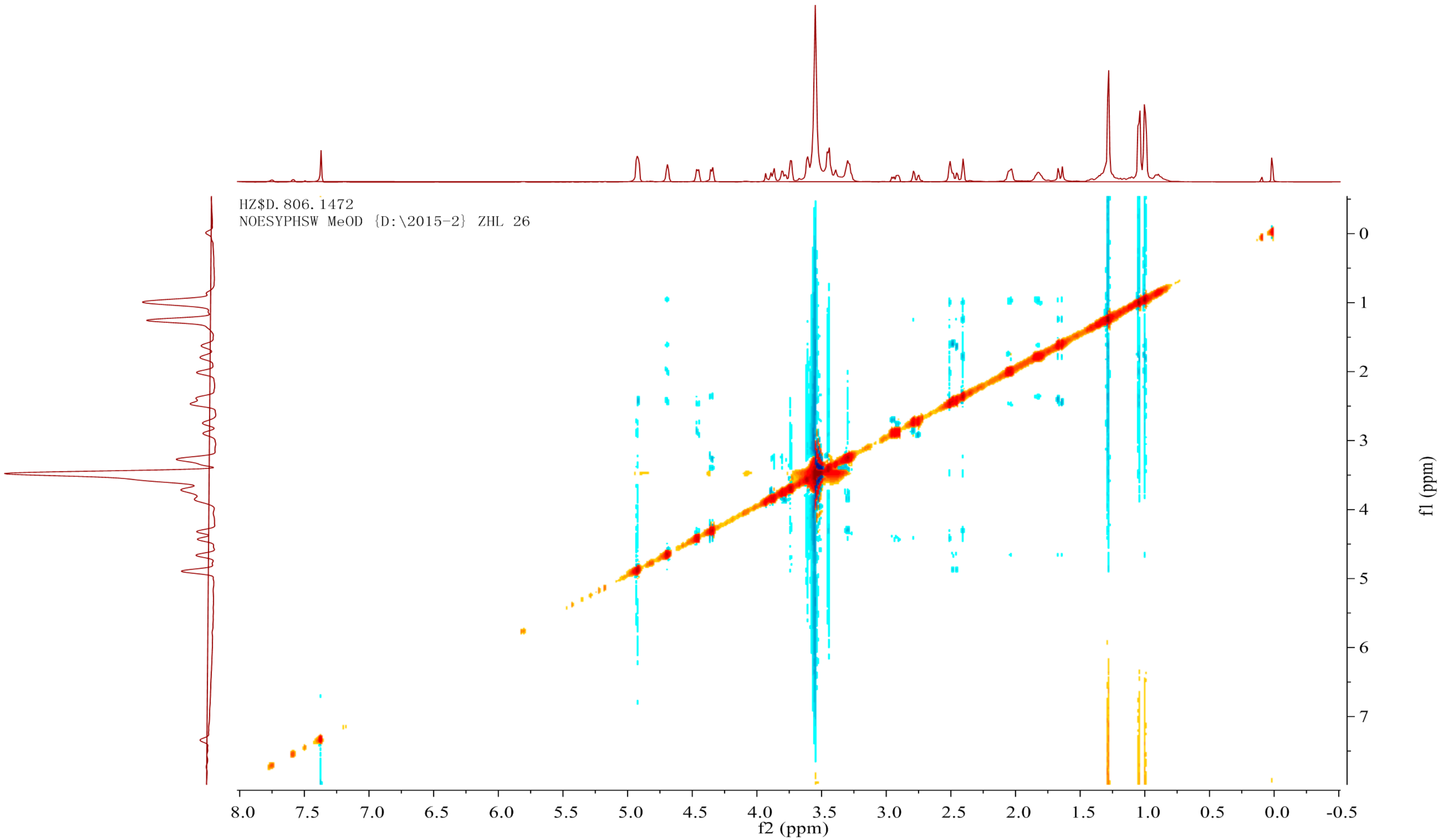

Figure S24. NOESY spectra of compound **2** in CD_3OD.

Elemental Composition Report

Page 1

Single Mass Analysis

Tolerance = 5.0 PPM / DBE: min = -1.5, max = 50.0
Element prediction: Off
Number of isotope peaks used for i-FIT = 3

Monoisotopic Mass, Even Electron Ions
161 formula(e) evaluated with 1 results within limits (up to 50 closest results for each mass)
Elements Used:
C: 5-80 H: 2-120 O: 0-20 Na: 0-1

wyy-10 LCT PXE KE324 14-Oct-2015
14:02:31
wyy-10_1014 40 (0.863) AM2 (Ar,10000.0,0.00,1.00); ABS; Cm (27:40) 1: TOF MS ES+
7.41e+003

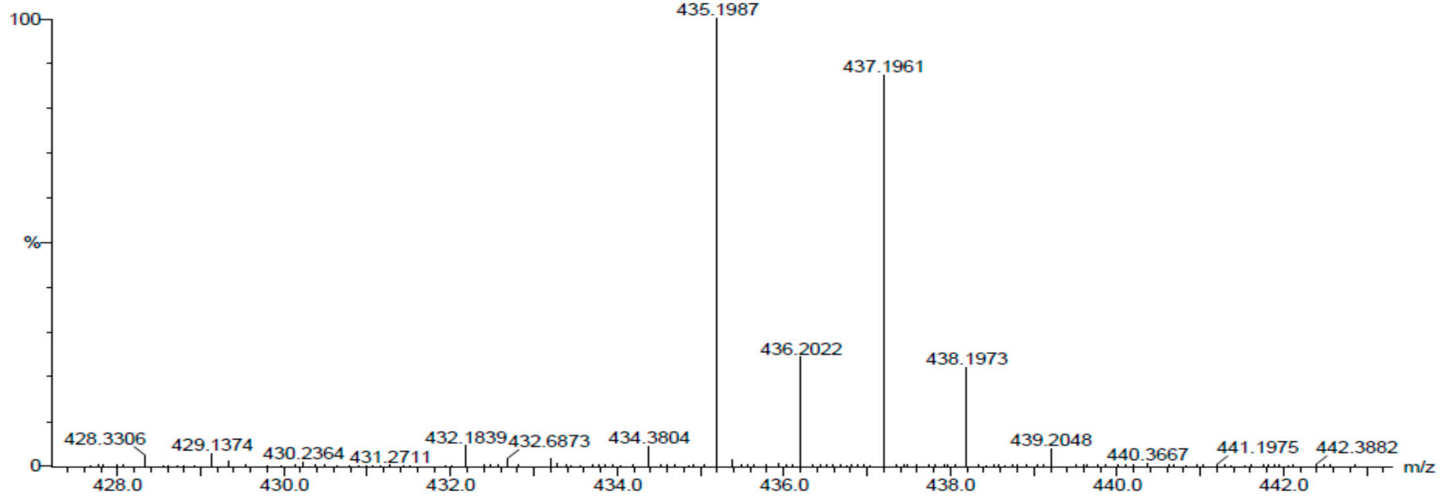

Minimum:				-1.5			
Maximum:		5.0	5.0	50.0			
Mass	Calc. Mass	mDa	PPM	DBE	i-FIT	i-FIT (Norm)	Formula
435.1987	435.1995	-0.8	-1.8	5.5	141.0	0.0	C21 H32 O8 Na

Figure S25. HRESIMS spectrum of compound **2.**

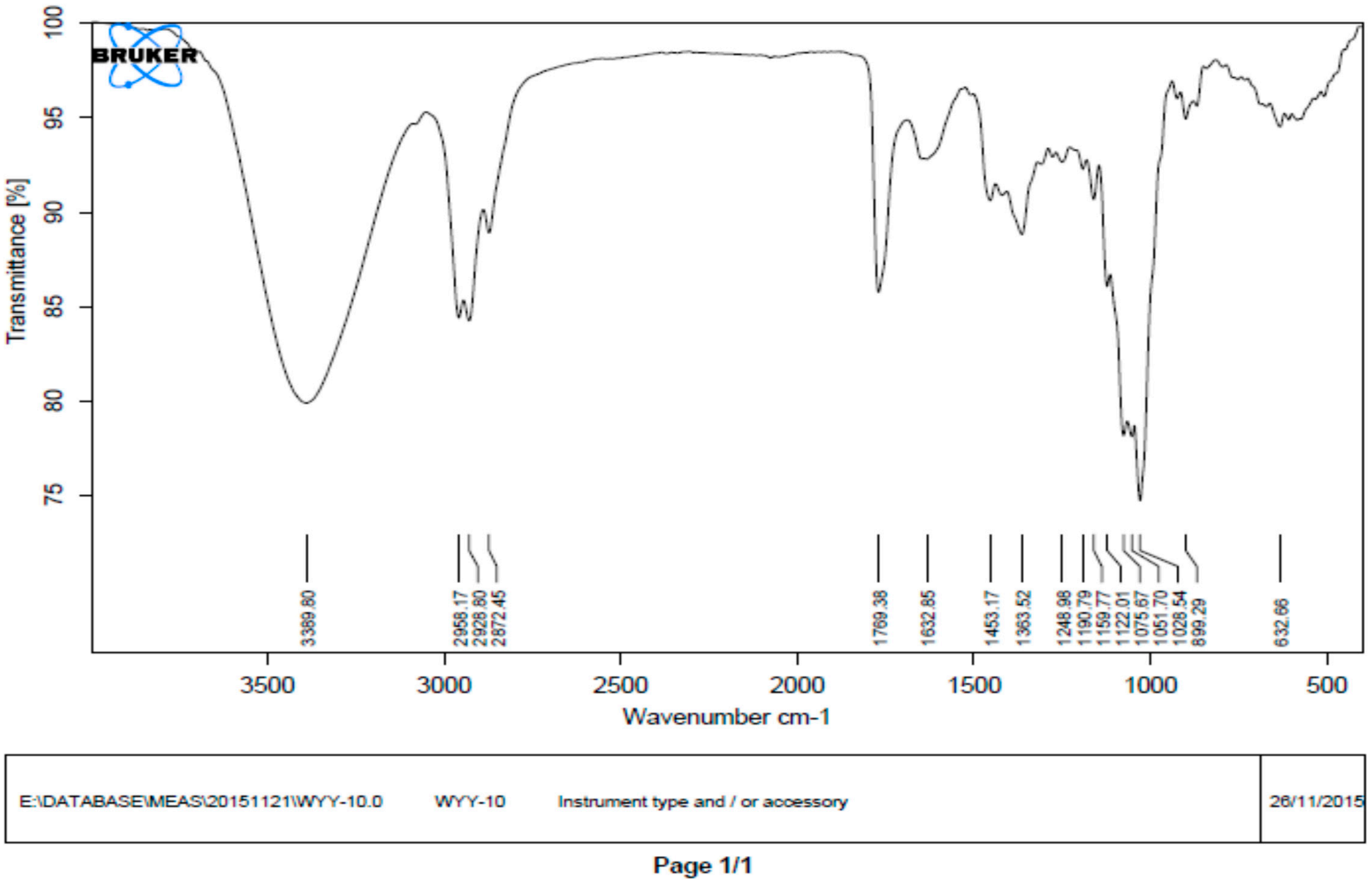

Figure S26. IR spectra of compound **2.**

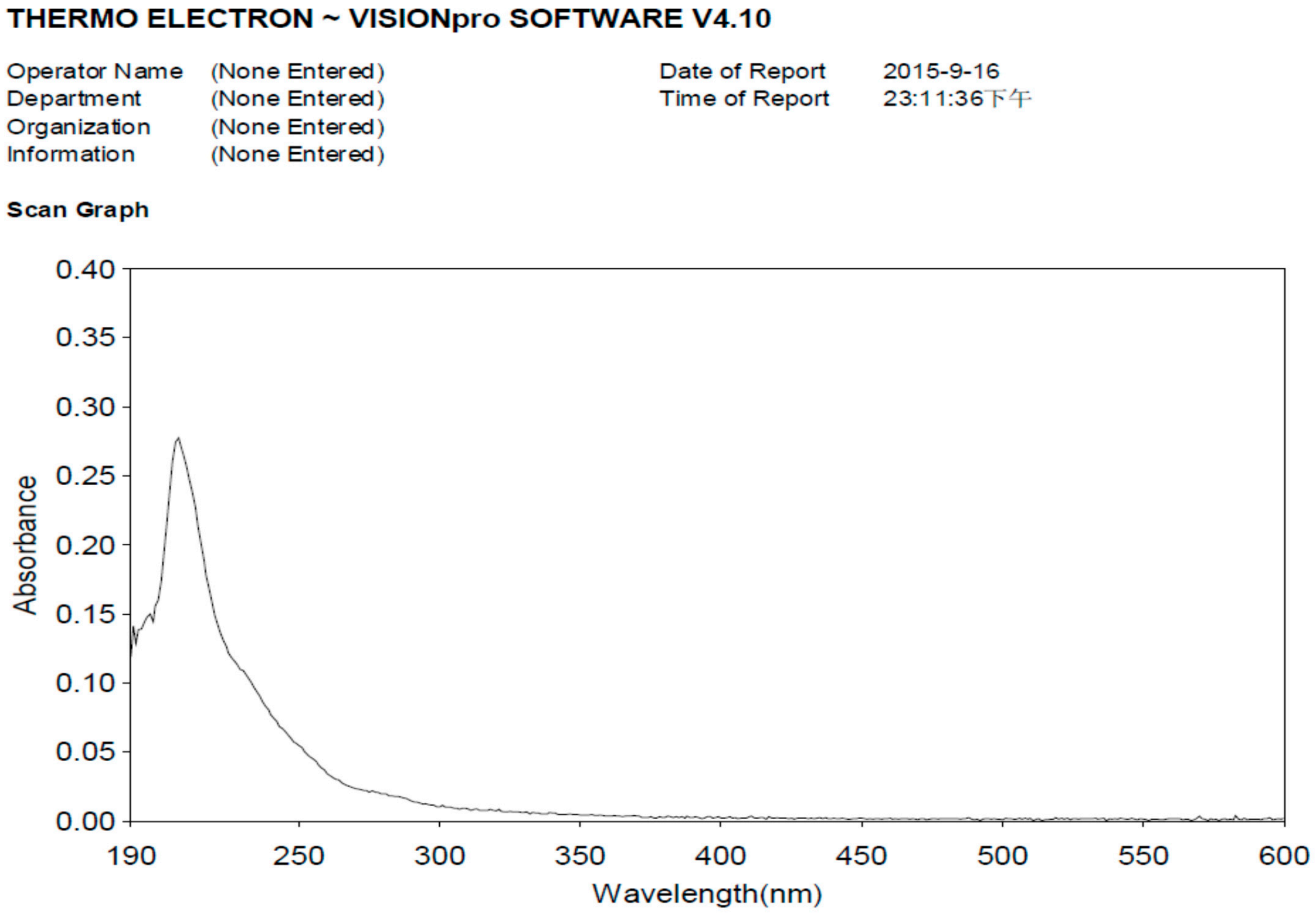

Figure S27. UV spectra of compound **2.**

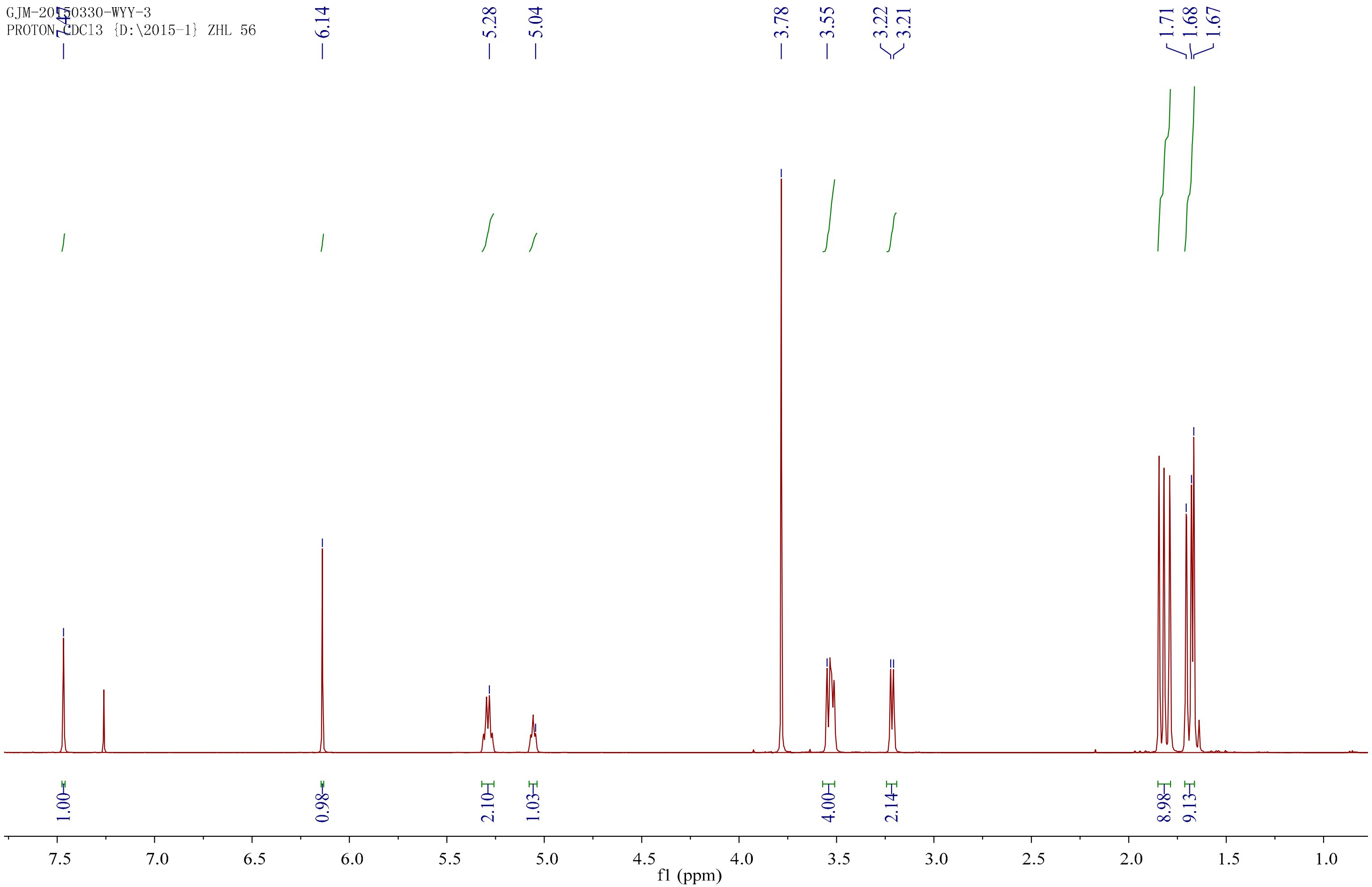

Figure S28. ^{1}H-NMR spectra of compound **3** in $CDCl_3$.

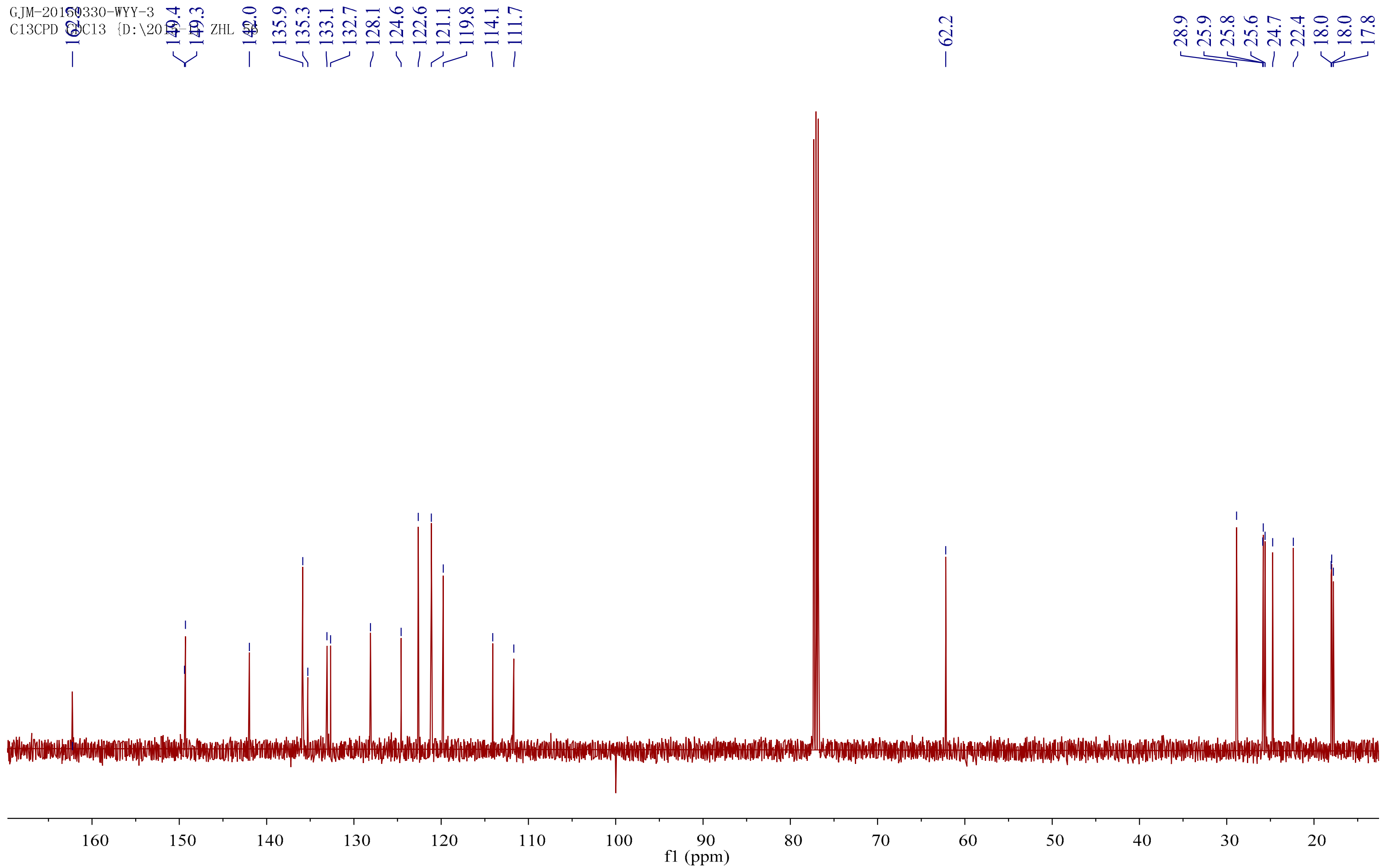

Figure S29. ^{13}C-NMR spectra of compound **3** in $CDCl_3$.

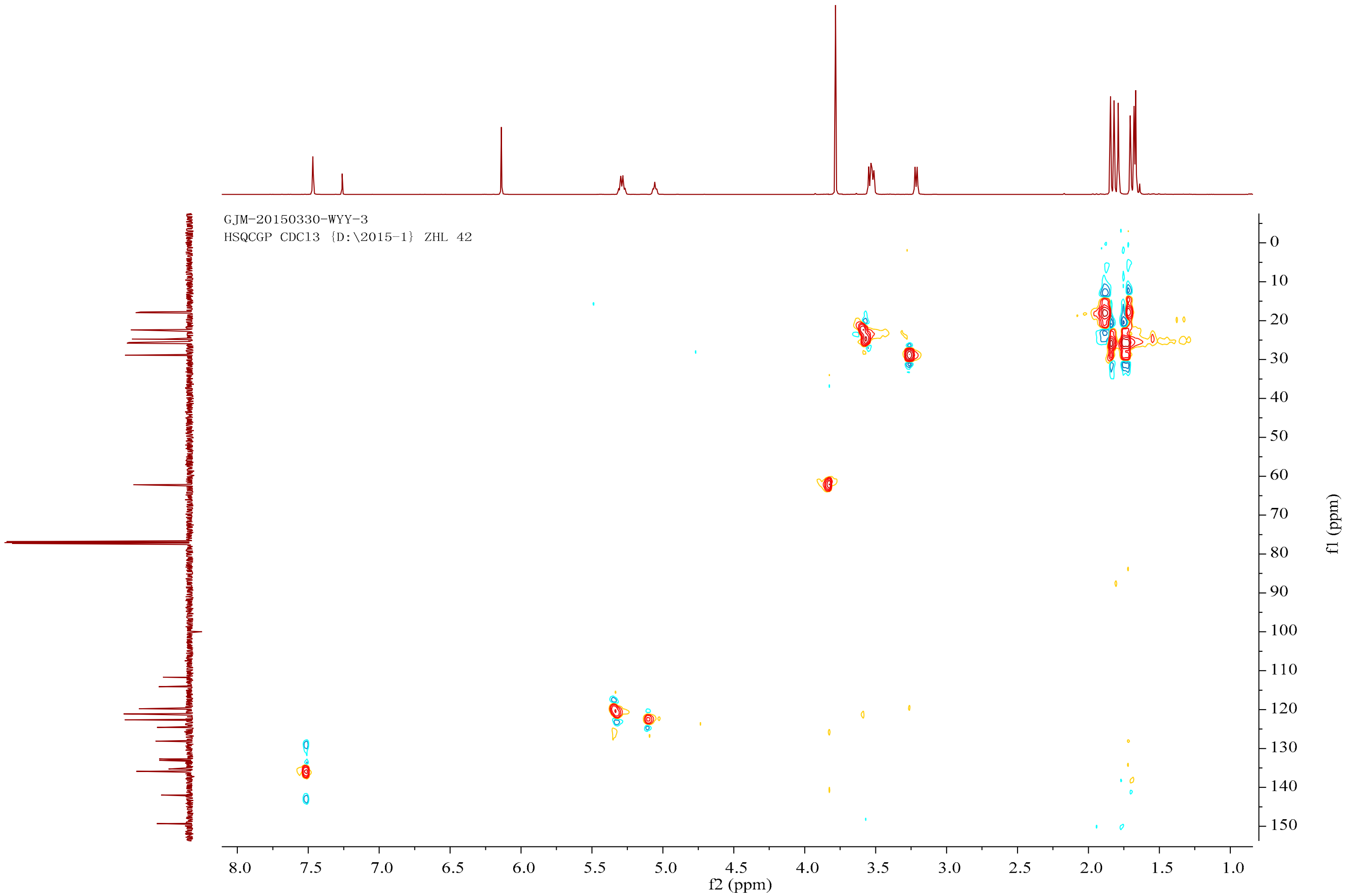

Finger S30. HSQC spectra of compound **3** in $CDCl_3$.

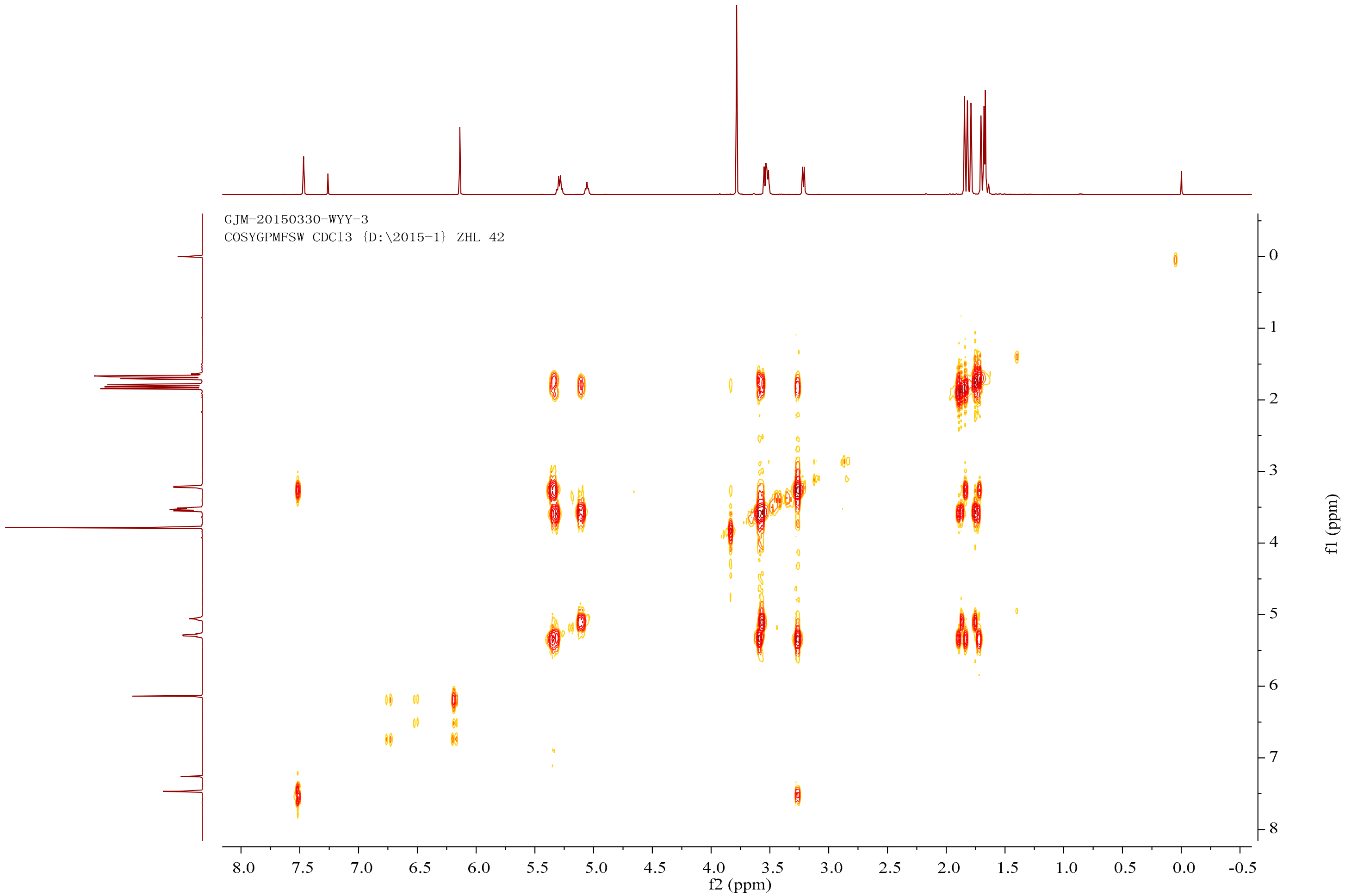

Finger S31. ^1H-^1H COSY spectra of compound **3** in $CDCl_3$.

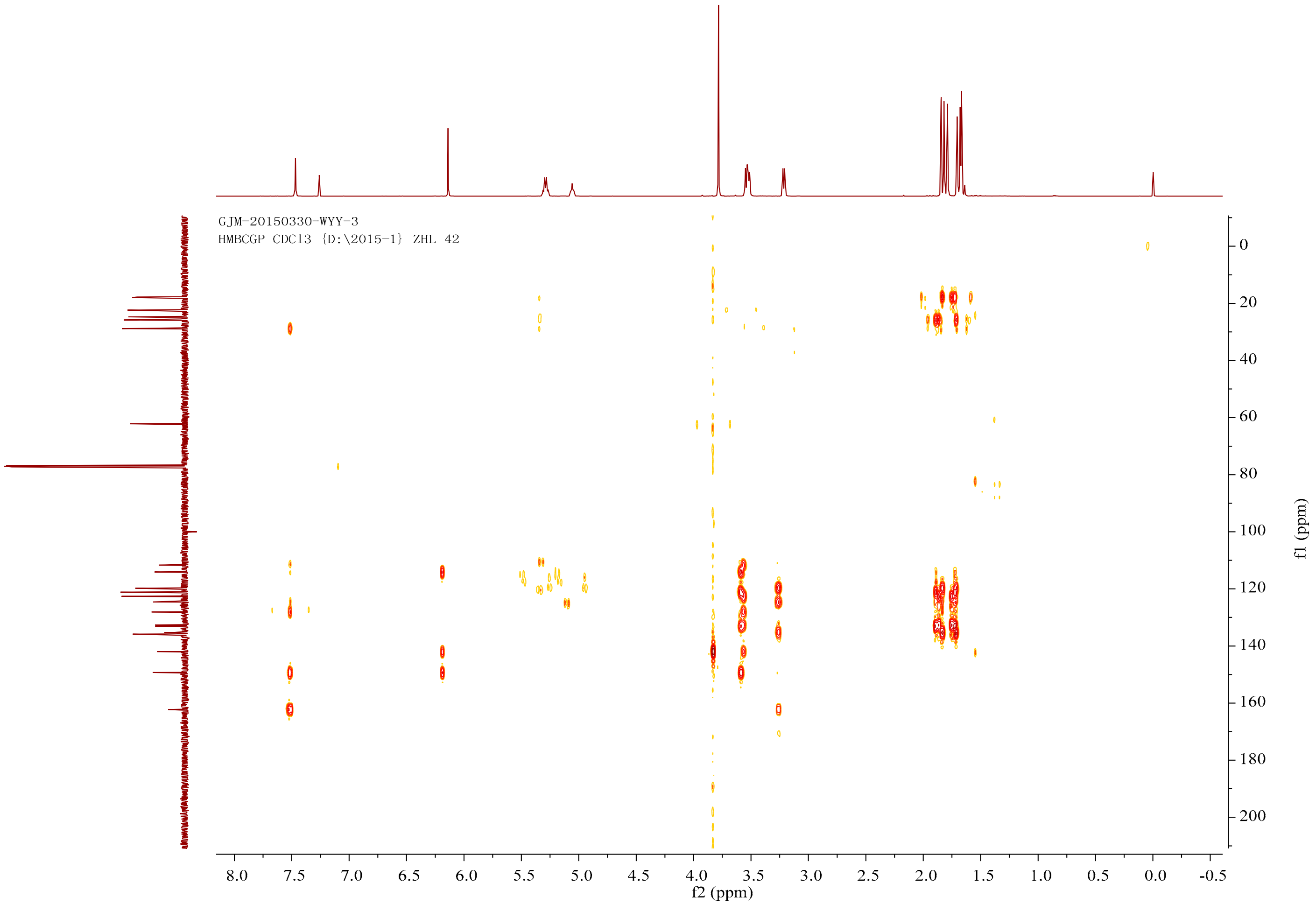

Finger S32. HMBC spectra of compound **3** in $CDCl_3$.

Elemental Composition Report **Page 1**

Single Mass Analysis
Tolerance = 5.0 PPM / DBE: min = -1.5, max = 50.0
Element prediction: Off
Number of isotope peaks used for i-FIT = 3

Monoisotopic Mass, Even Electron Ions
140 formula(e) evaluated with 1 results within limits (up to 50 closest results for each mass)
Elements Used:
C: 5-80 H: 2-120 O: 0-20 Na: 0-1

wyy-3n LCT PXE KE324 14-Oct-2015 13:55:10
wyy-3n_10104 23 (0.495) AM2 (Ar,10000.0,0.00,1.00); ABS; Cm (10:23) 1: TOF MS ES- 7.84e+003

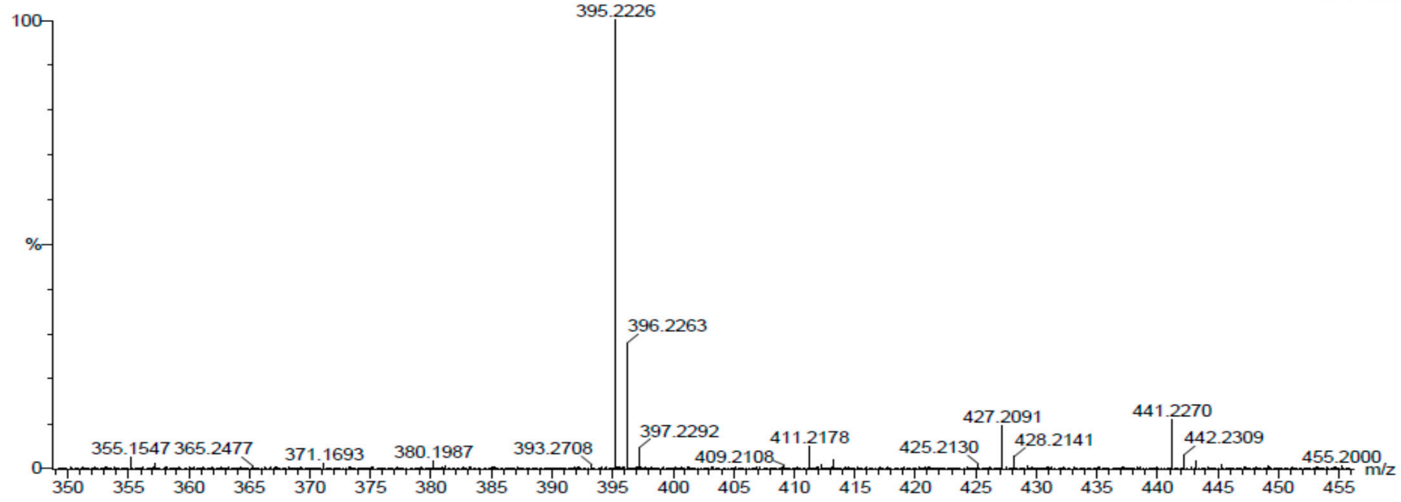

Minimum:				-1.5			
Maximum:		5.0	5.0	50.0			
Mass	Calc. Mass	mDa	PPM	DBE	i-FIT	i-FIT (Norm)	Formula
395.2226	395.2222	0.4	1.0	10.5	79.5	0.0	C25 H31 O4

Figure S33. HRESTMS of compound **3** in CD_3OD.

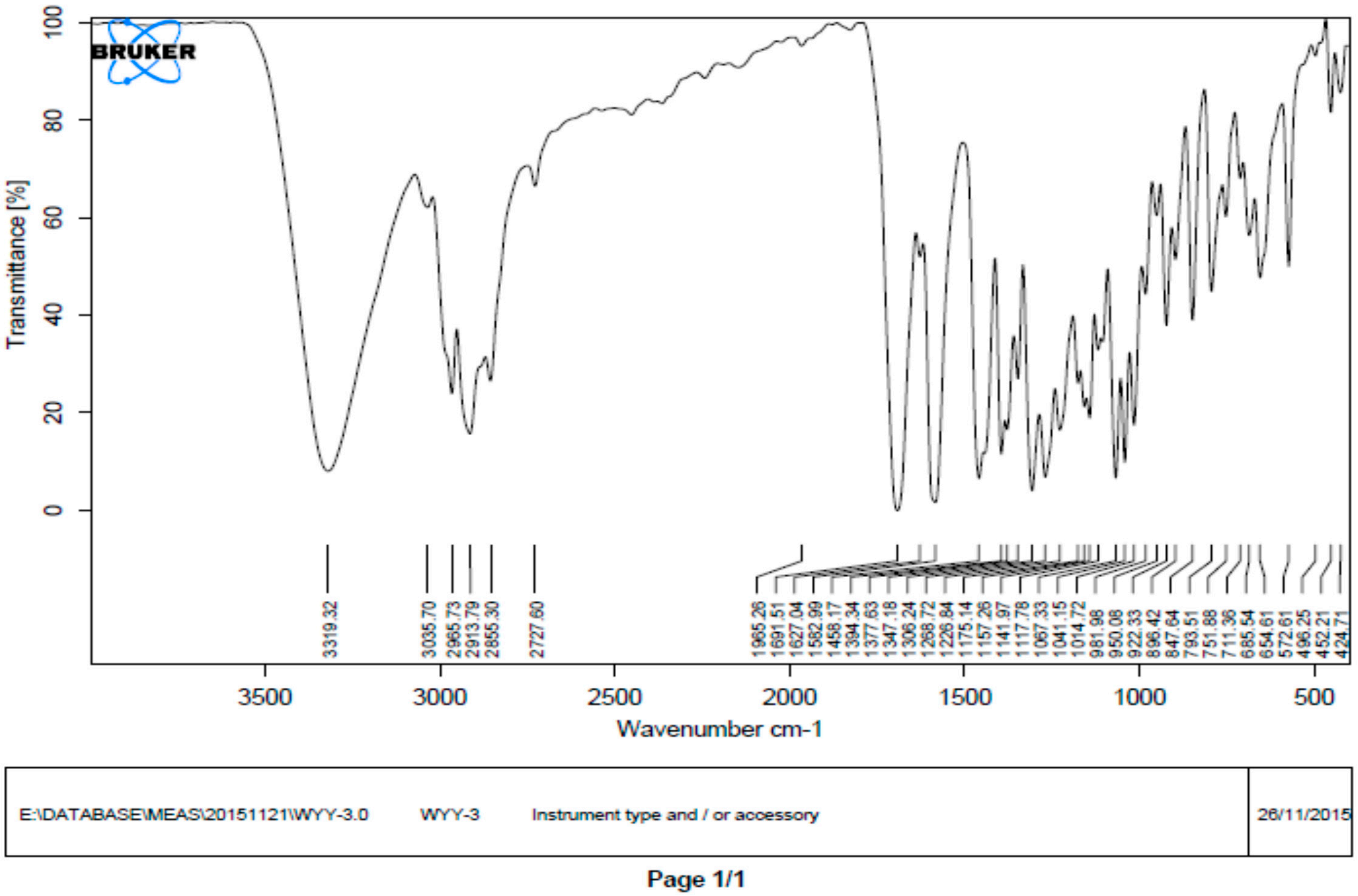

Figure S34. IR spectra of compound **3.**

THERMO ELECTRON ~ VISIONpro SOFTWARE V4.10

Operator Name	(None Entered)	Date of Report	2015-9-23
Department	(None Entered)	Time of Report	12:36:01下午
Organization	(None Entered)		
Information	(None Entered)		

Scan Graph

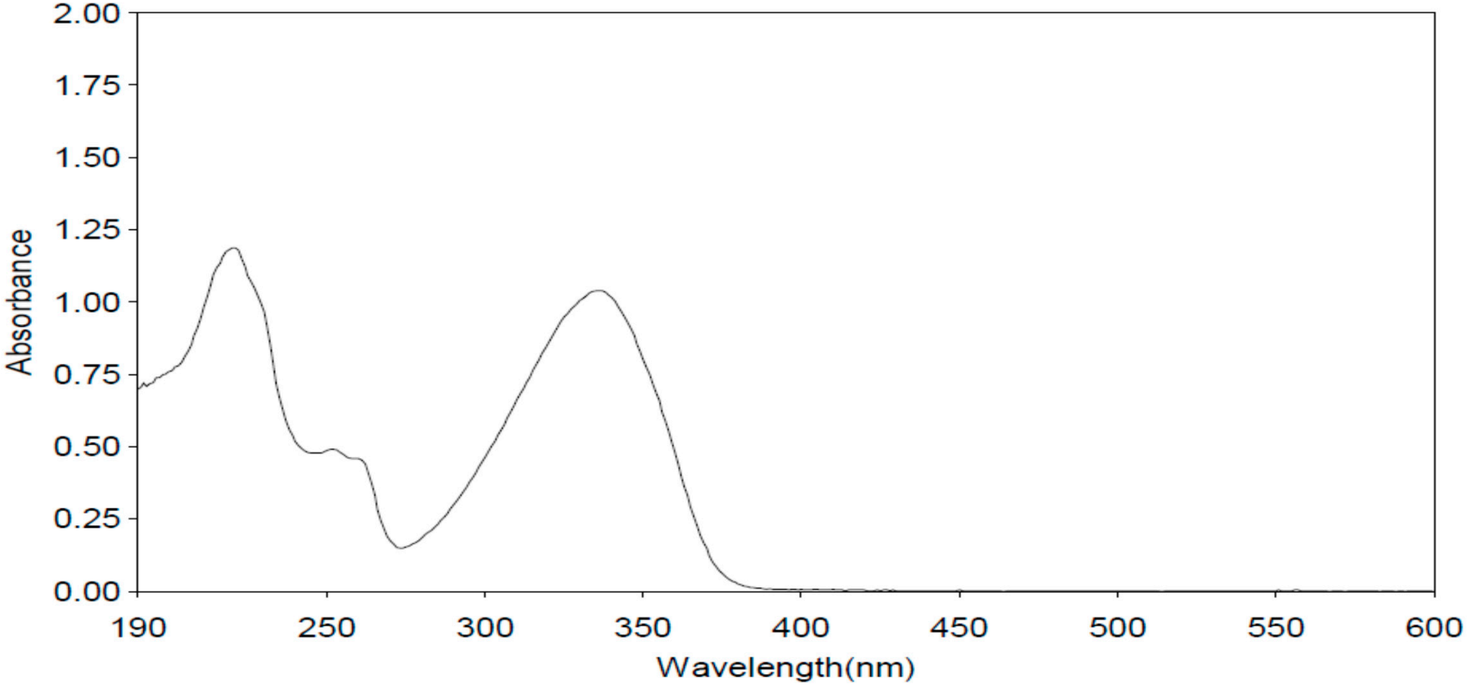

Figure S35. UV spectra of compound **3.**

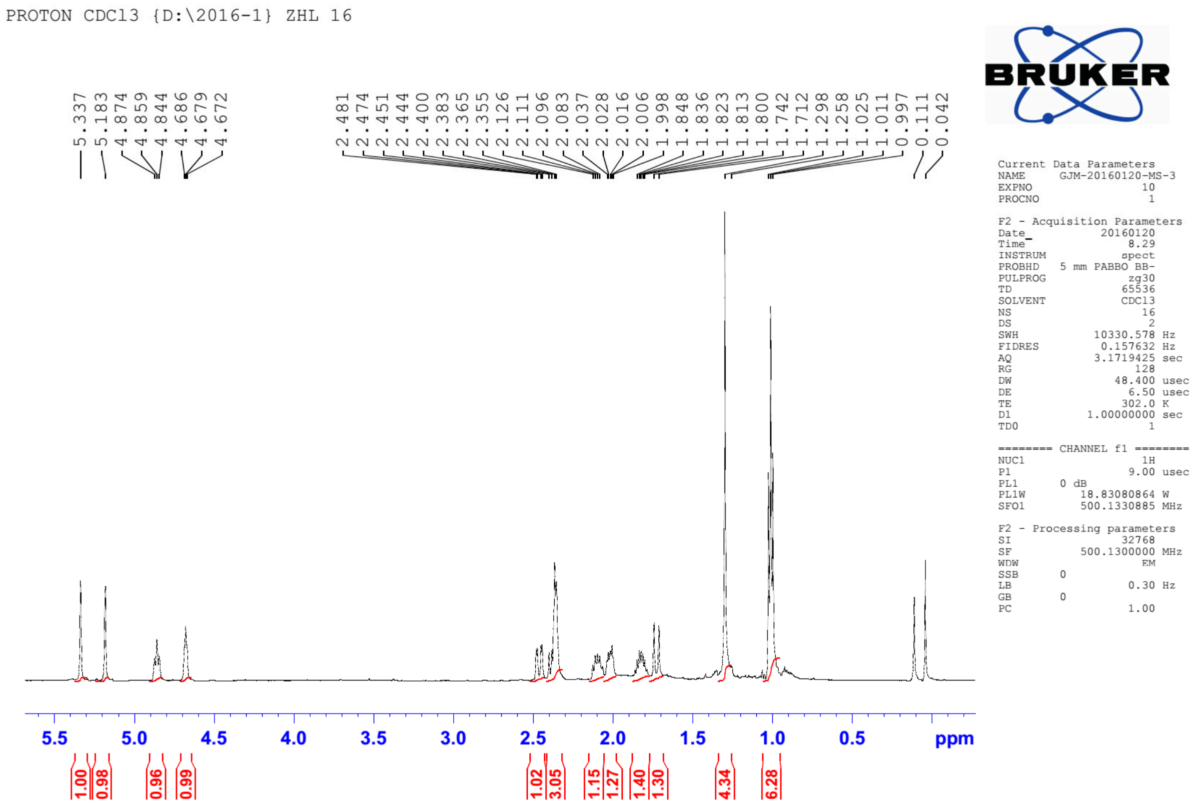

Figure S36. ^{1}H-NMR spectra of compound **1b** in $CDCl_3$.

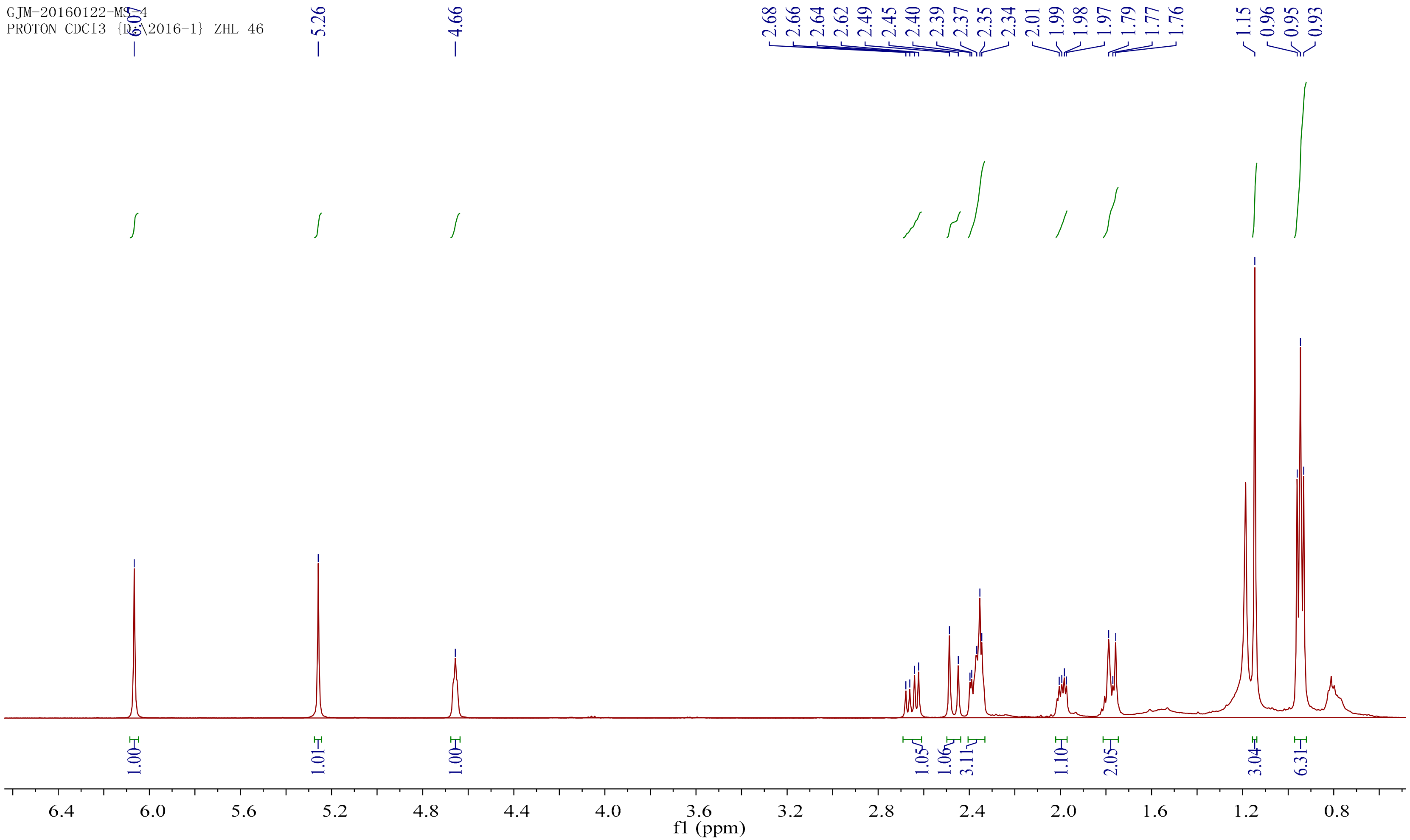

Figure S37. ^{1}H-NMR spectra of compound **1c** in $CDCl_3$.

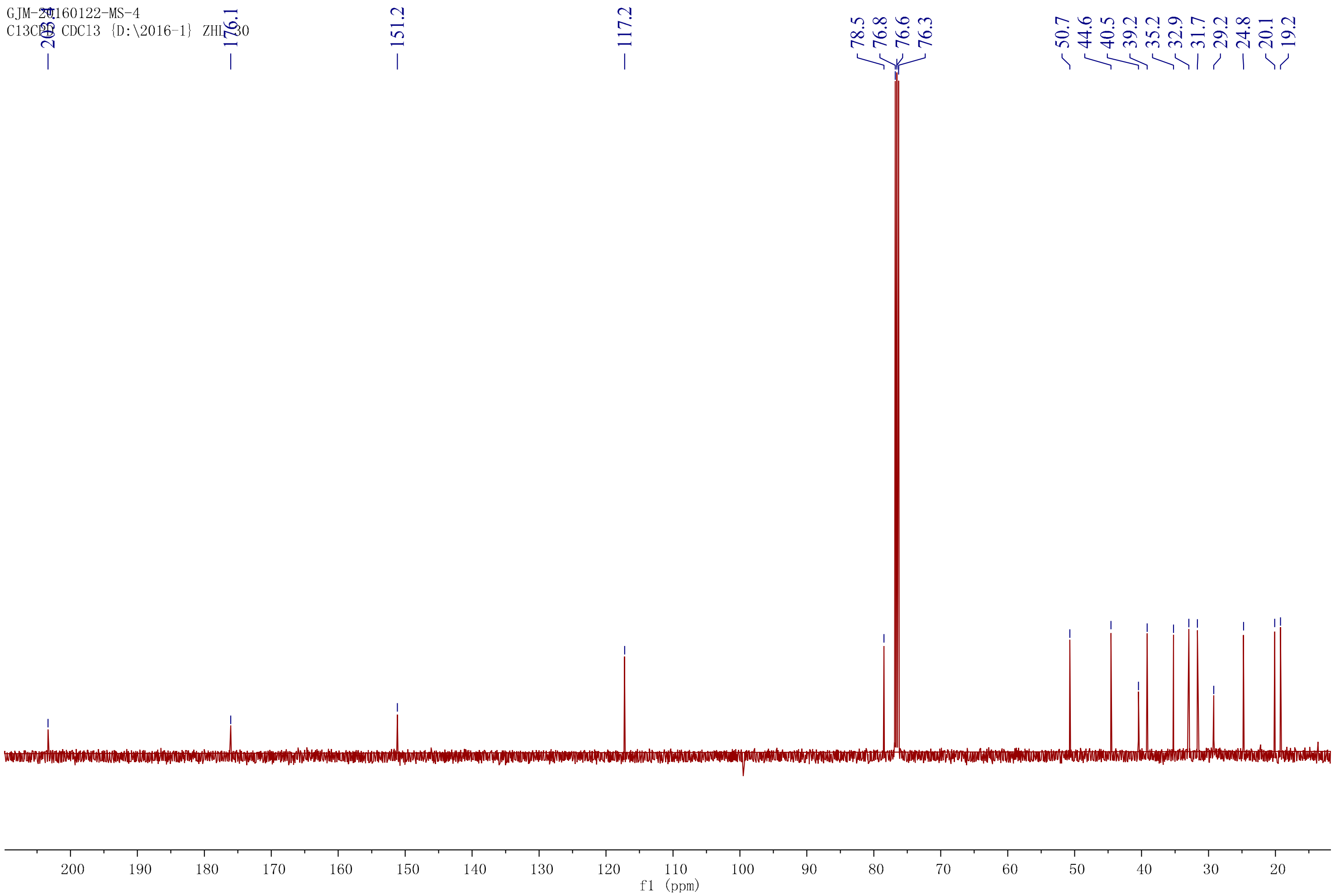

Figure S38. ^{13}C-NMR spectra of compound **1c** in $CDCl_3$.

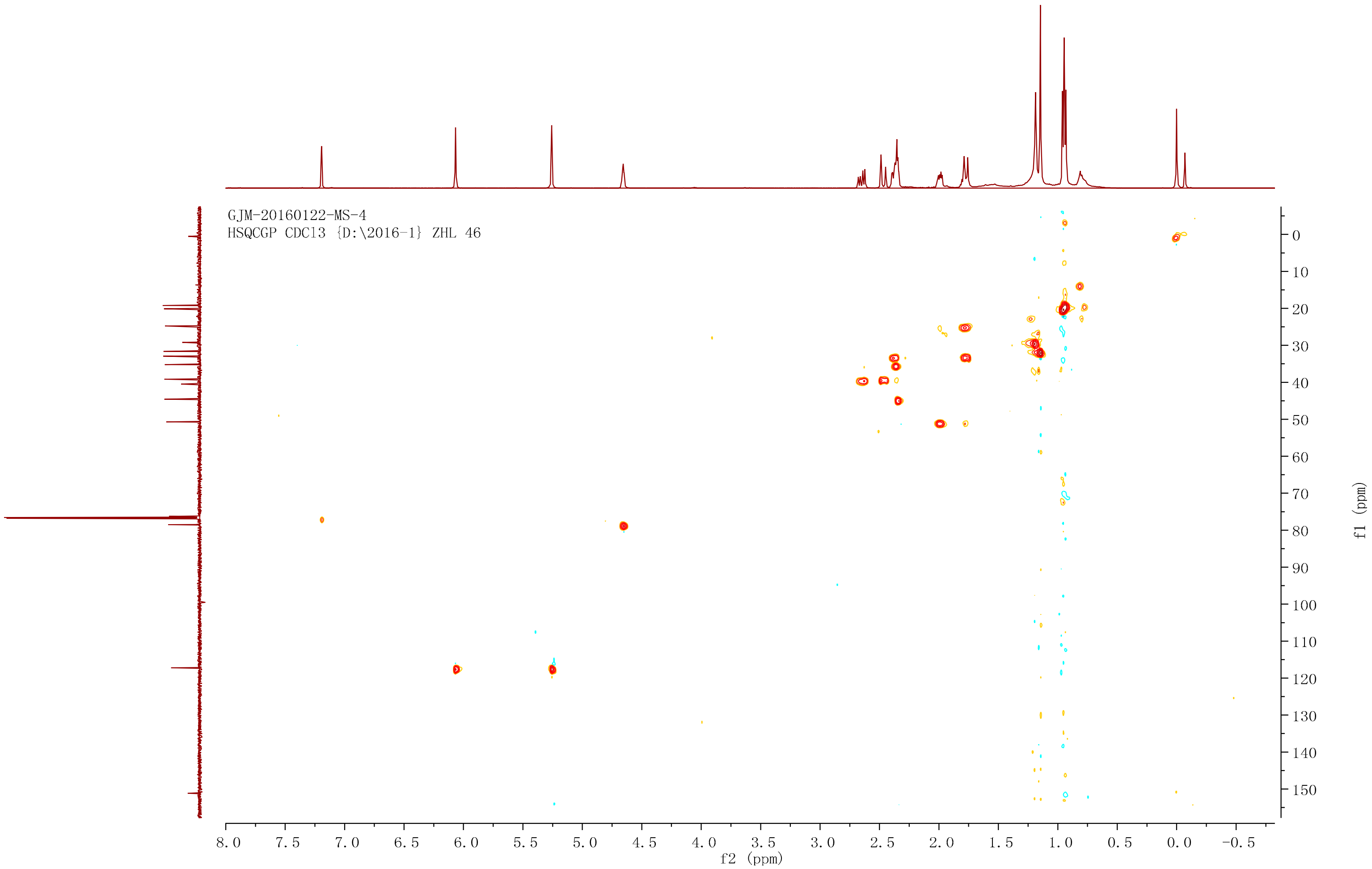

Finger S39. HSQC spectra of compound **1c** in $CDCl_3$.

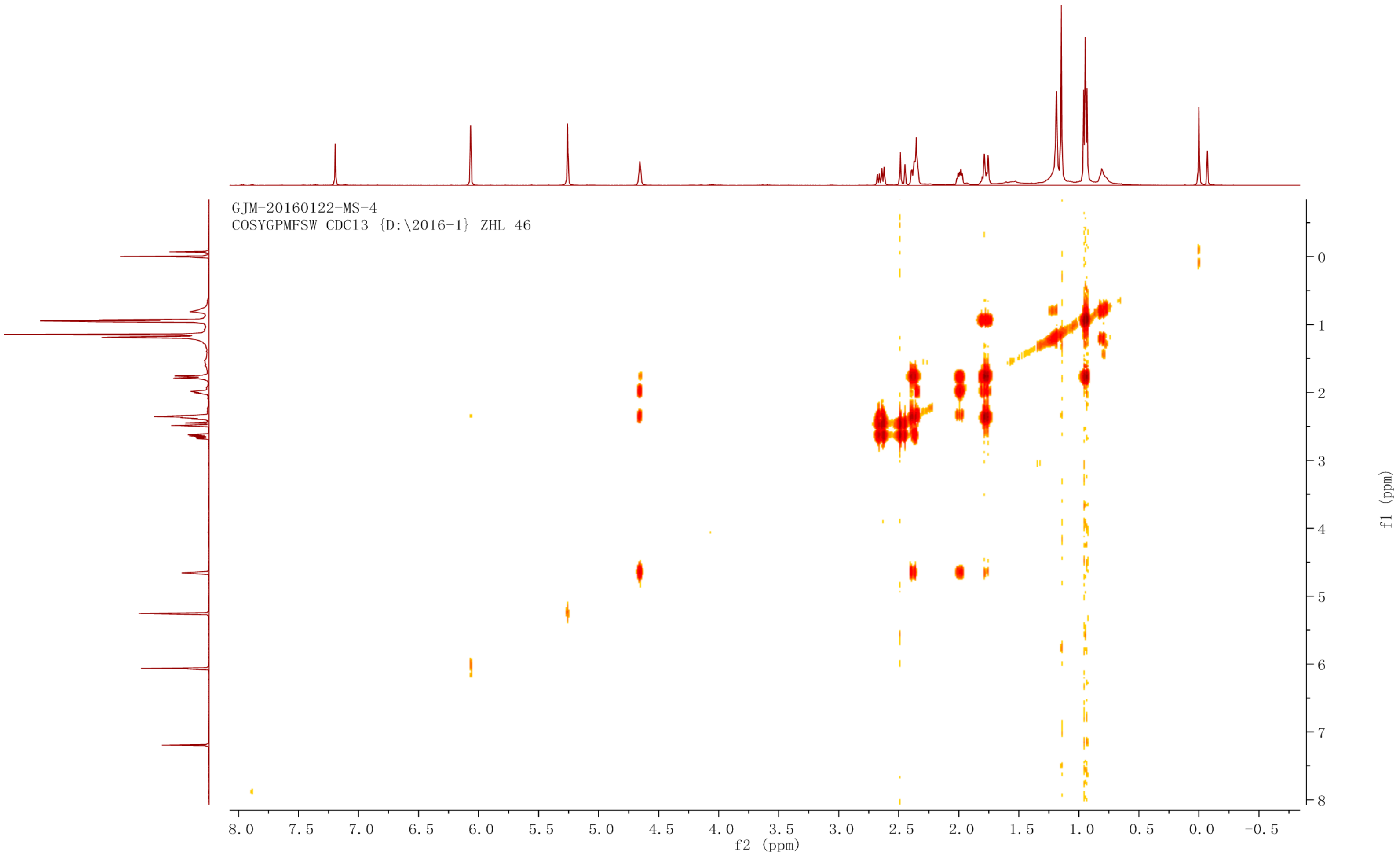

Finger S40. ^1H-^1H COSY spectra of compound **1c** in $CDCl_3$.

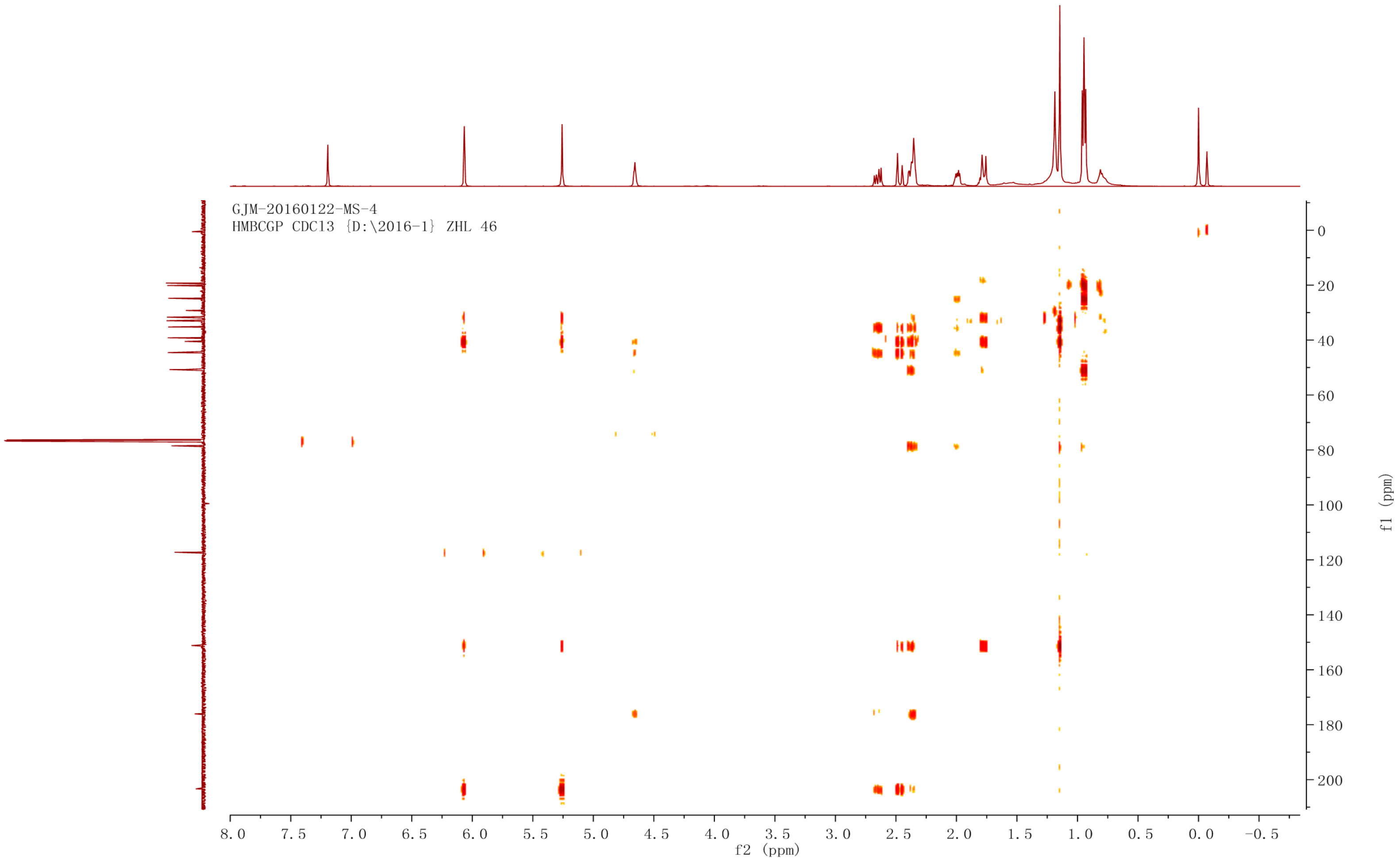

Figure S41. HMBC spectra of compound **1c** in $CDCl_3$.

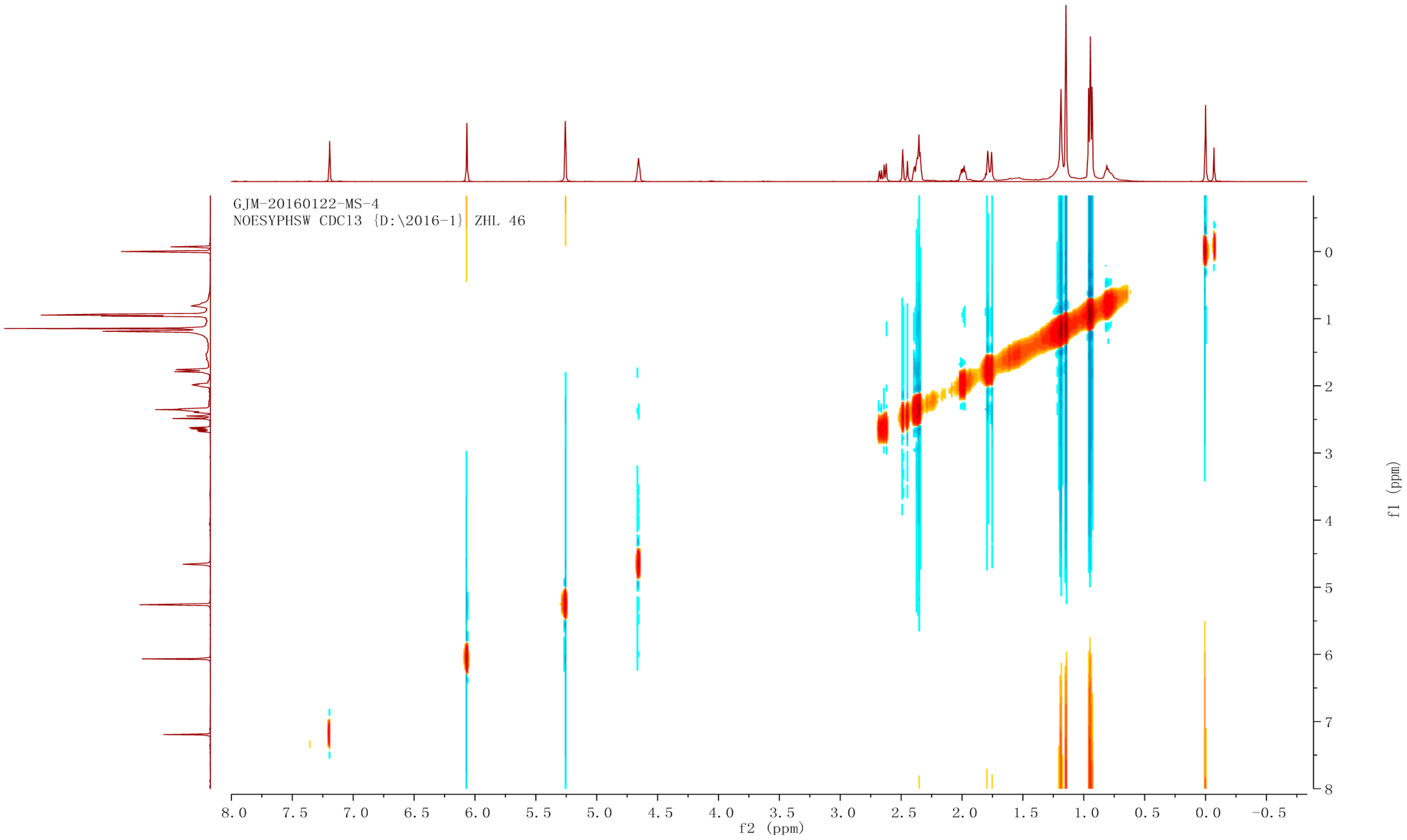

Figure S42. NOESY spectra of compound **1c** in $CDCl_3$.

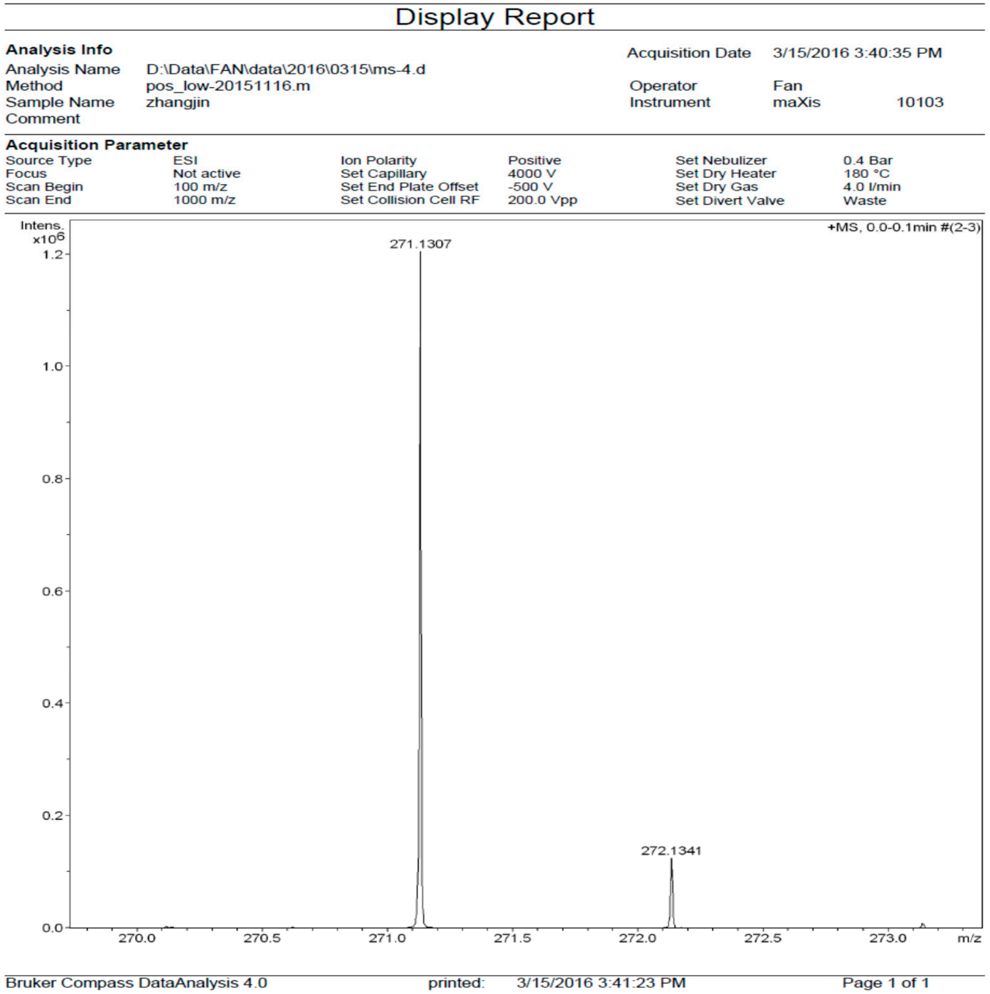

Figure S43. HRESIMS spectrum of compound **1c.**

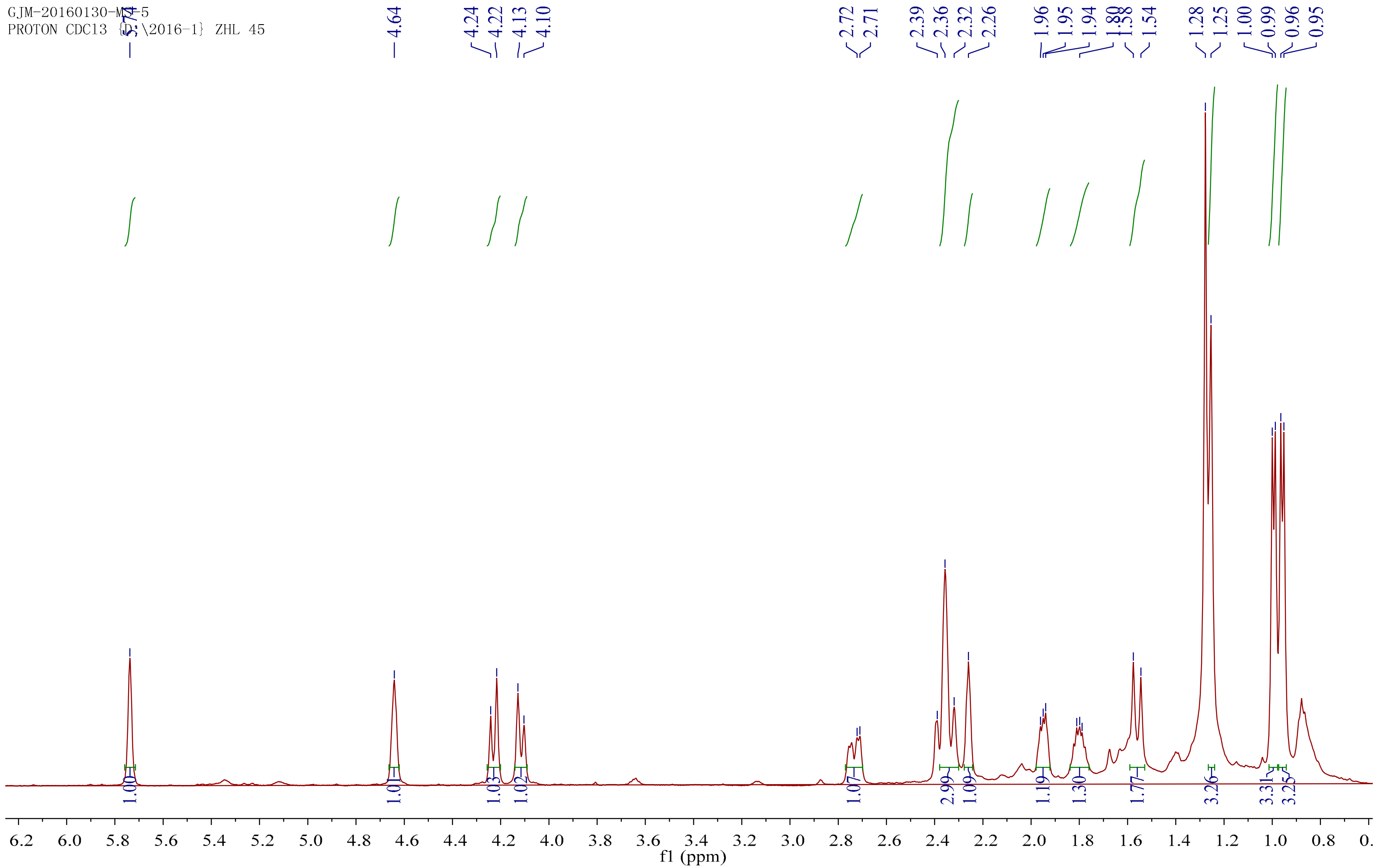

Figure S44. ^{1}H-NMR spectra of compound **1d** in $CDCl_3$.

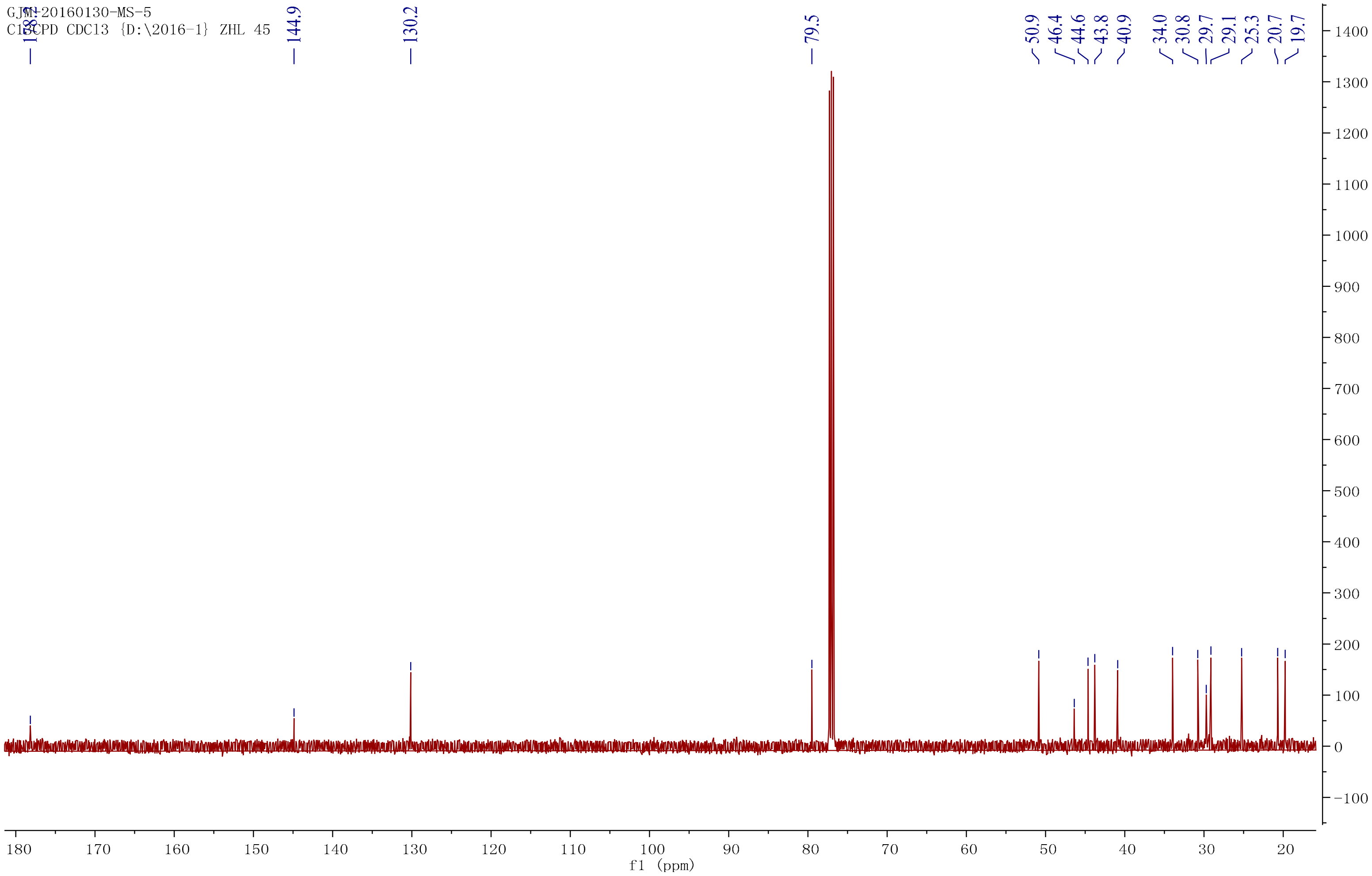

Figure S45. ^{13}C-NMR spectra of compound **1d** in $CDCl_3$.

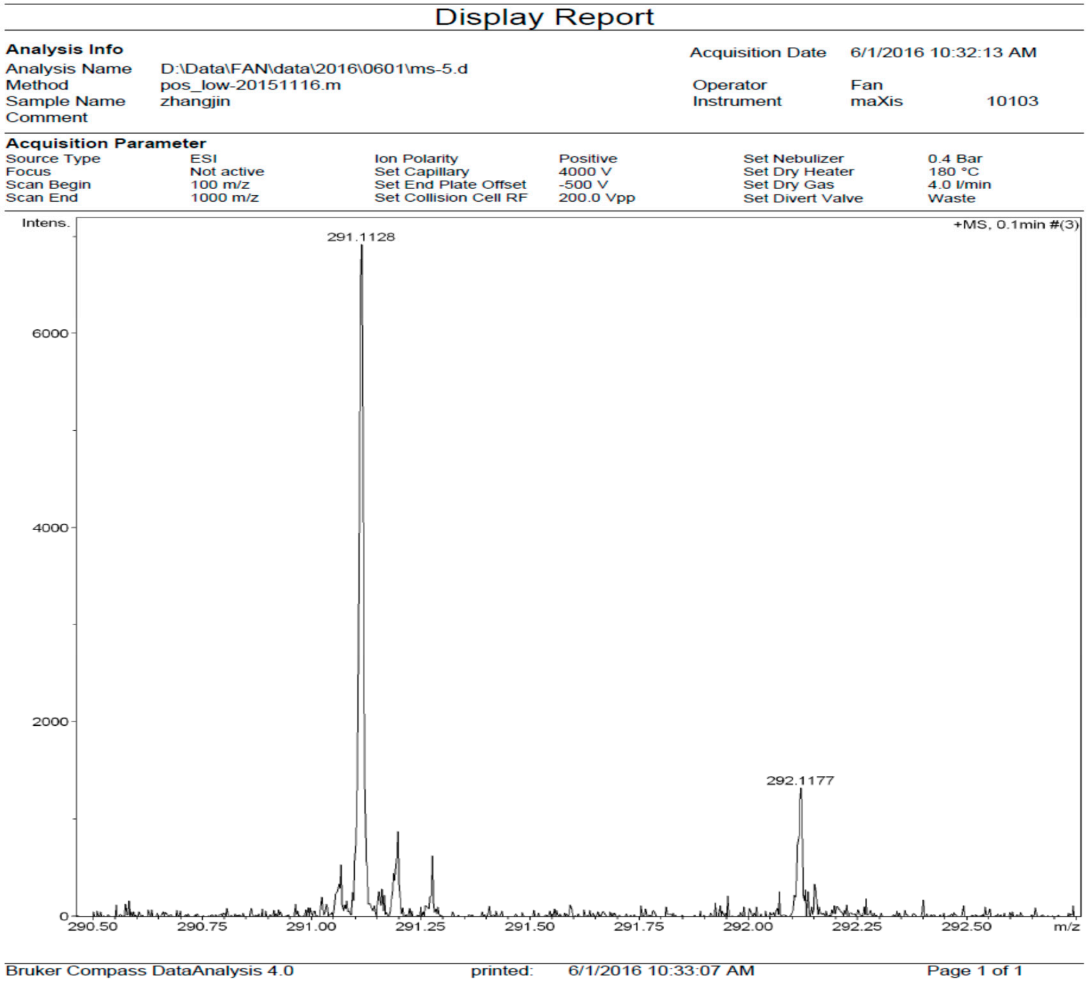

Figure S46. HRESIMS spectrum of compound **1d.**

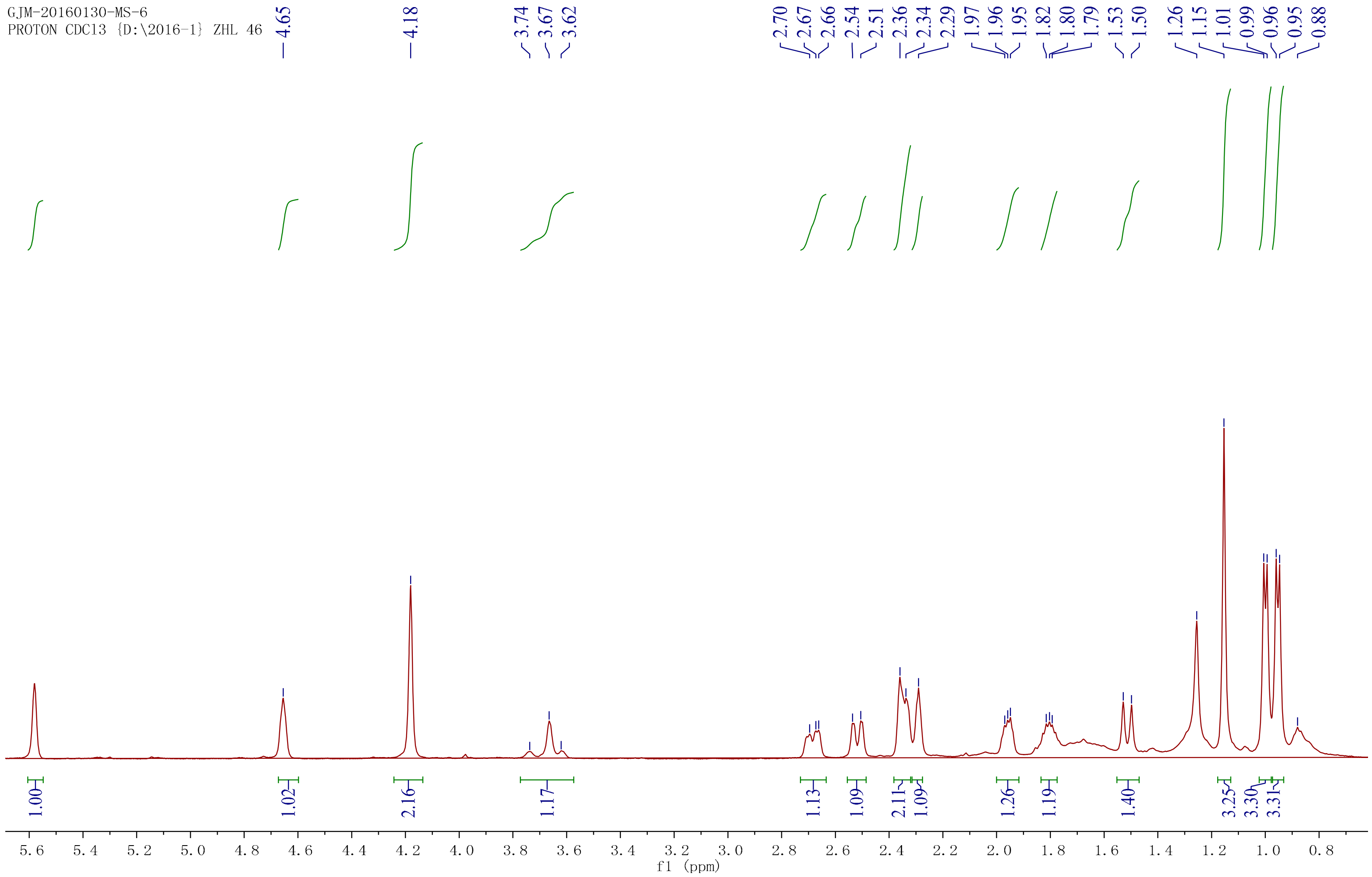

Figure S47. ^{1}H-NMR spectra of compound **1e** in $CDCl_3$.

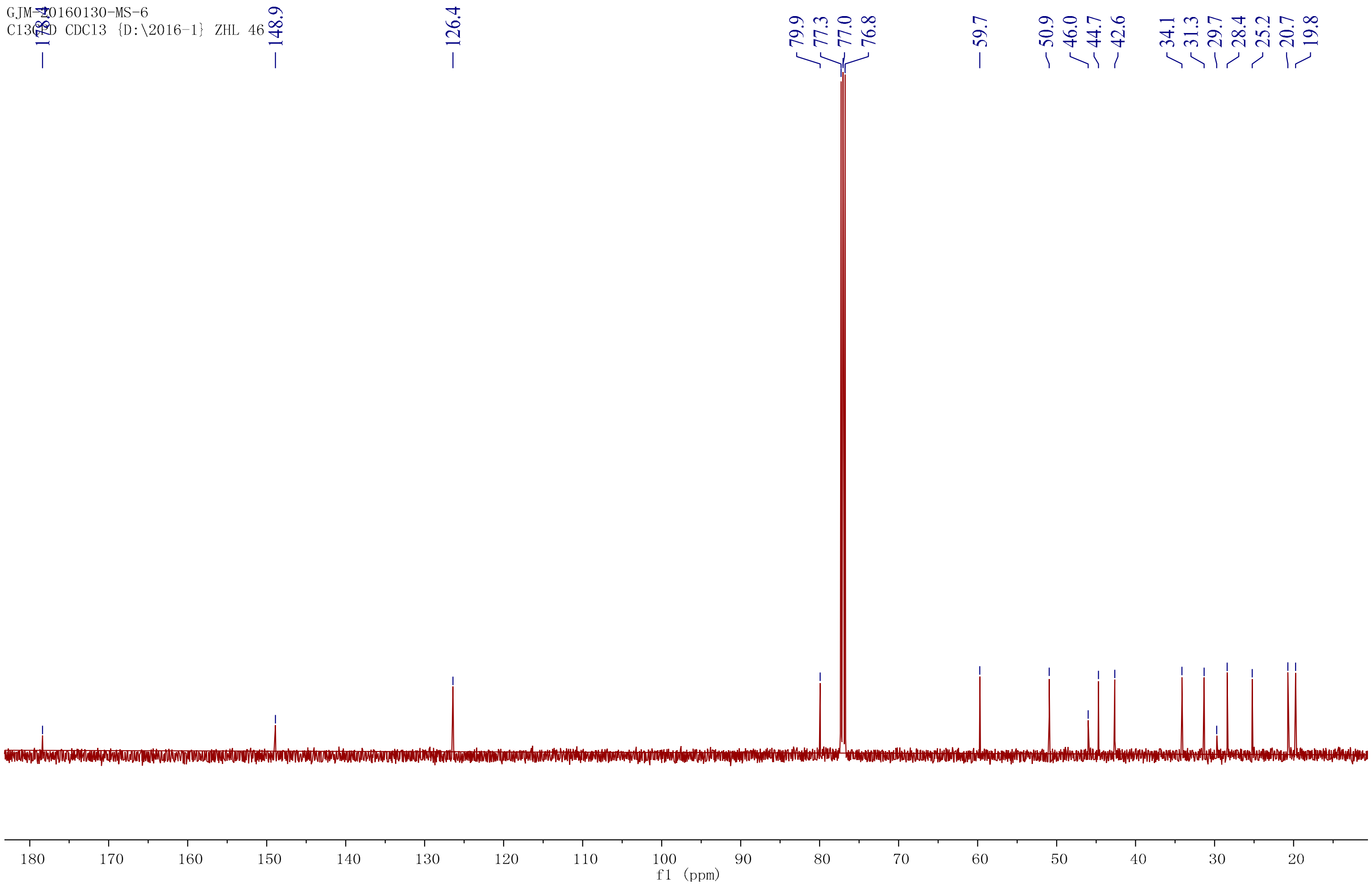

Figure S48. ^{13}C-NMR spectra of compound **1e** in $CDCl_3$.

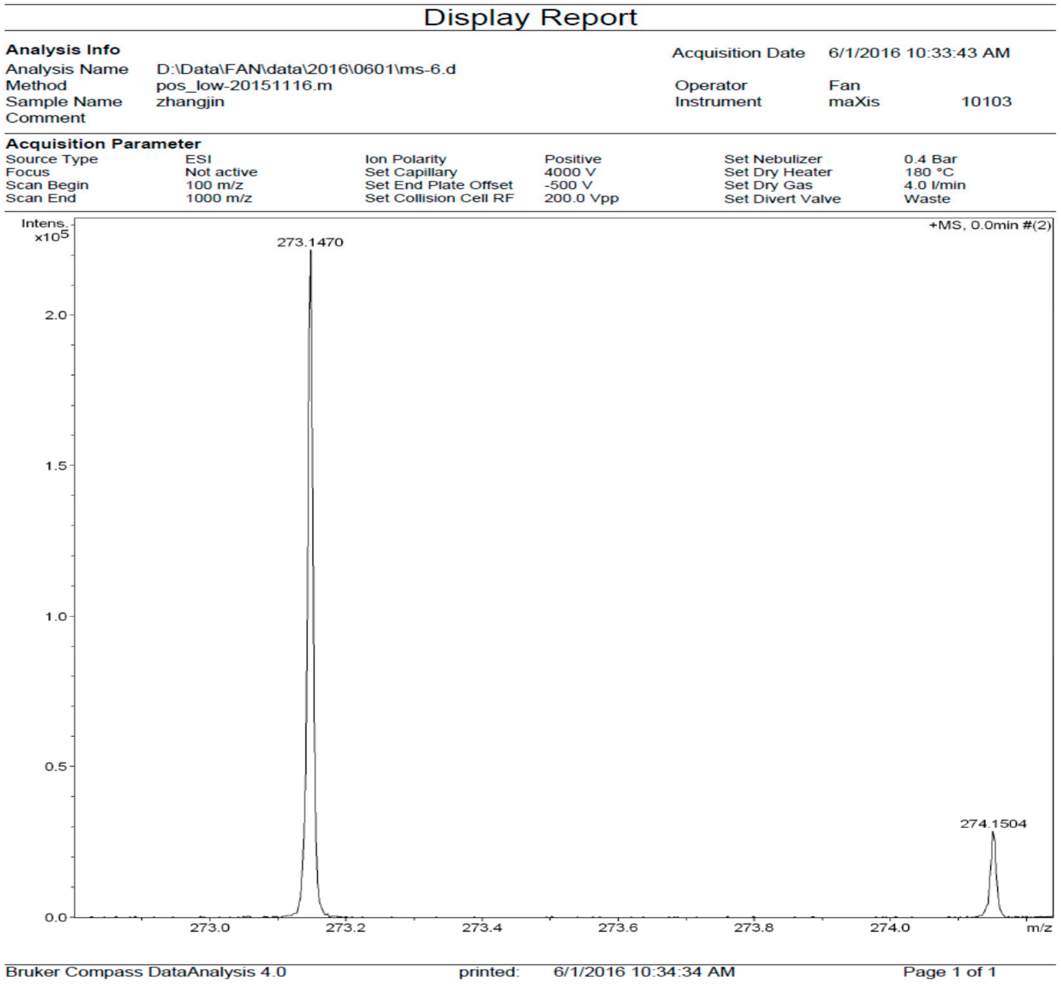

Figure S49. HRESIMS spectrum of compound **1e**.
